# Gray matter atrophy mediates the association between cerebral hypometabolism and cognitive impairment in Alzheimer’s disease

**DOI:** 10.3389/fneur.2026.1919778

**Published:** 2026-09-15

**Authors:** Shiqi Zhao, Jianwen Chen, Peixue Li, Li Zhao, Chunbo Dong, Lei Yu

**Affiliations:** 1 Department of Neurology, The First Affiliated Hospital of Dalian Medical University, Dalian, Liaoning, China; 2 The Center for Gerontology and Geriatrics, Dalian Friendship Hospital, Dalian, Liaoning, China

**Keywords:** Alzheimer’s disease, amyloid-β, APoE ε4, FDG-PET, gray matter atrophy, multimodal imaging

## Abstract

**Objective:**

Alzheimer’s disease (AD) is characterized by amyloid deposition, brain hypometabolism, gray matter atrophy and cognitive decline, yet their causal interactions are poorly defined. This multimodal PET/MRI study explored regional gray matter volume (GMV) loss and glucose hypometabolism in AD patients, and clarified their correlations with distinct cognitive deficits alongside mediating pathways.

**Methods:**

134 probable dementia patients were split into AD and non-AD dementia cohorts based on ^18^F-AV45 PET amyloid-β (Aβ) load, all receiving structural MRI, ^18^F-FDG and ^18^F-AV45 PET scans. We compared intergroup GMV and metabolic discrepancies, analyzed imaging-cognition correlations adjusted for clinical and demographic confounders, and implemented mediation models to test whether GMV bridges cerebral metabolism and cognition.

**Results:**

AD patients exhibited prominent regional GMV shrinkage and FDG hypometabolism in vulnerable brain regions relative to non-AD dementia subjects. Covariate-adjusted analyses linked both imaging biomarkers to domain-specific cognitive performance. Primary mediation analyses showed that regional GMV significantly mediated the associations between FDG metabolism and visuospatial, executive, and language function, accounting for 40.36, 35.60, and 49.99% of the total effects, respectively. Reverse mediation models identified only a weak indirect effect of right superior temporal gyrus metabolism on the association between right inferior parietal lobule GMV and visuospatial ability, accounting for 13.26% of the total effect. Moreover, after multiple-imputation sensitivity analyses, most indirect effects became unstable or nonsignificant, suggesting that the FDG metabolism–cognition association is predominantly direct, with limited evidence for GMV mediation.

**Conclusion:**

Integrated PET/MRI revealed AD-related patterns of regional atrophy and hypometabolism. Overall, the association between cerebral metabolic dysfunction and cognitive impairment appeared to be predominantly direct, with limited evidence supporting a mediating role of regional GMV.

## 1 Introduction

Alzheimer’s disease (AD) is a progressive neurodegenerative disorder characterized by gradual cognitive decline and loss of behavioral competence. With the rapid aging of global population, the prevalence of AD continues to rise, affecting approximately 52 million individuals worldwide and nearly 10 million patients in China (1). This burden poses significant medical and socioeconomic challenges for patients, families and society. To date, the exact etiology and pathogenesis of AD remain incompletely understood. The amyloid-β (Aβ) cascade hypothesis serves as one of core pathogenic theory, proposing that an imbalance between Aβ production and clearance triggers neuronal degeneration and subsequent cognitive deterioration (2). Notably, Aβ plaque deposition occurs prior to the progression of neurodegenerative changes and clinical sign of neurodegeneration, including cerebral atrophy and hypometabolism, ultimately determines the rate of clinical cognitive decline in AD (3).

Advanced neuroimaging approaches are now essential for the early diagnosis, pathological evaluation, and longitudinal monitoring of AD. Among these, magnetic resonance imaging (MRI) and positron emission tomography (PET) represent the most widely applied modalities in both clinical practice and research use. MRI enables multi-dimensional evaluation of early cerebral damage in AD at both structural and functional levels. Structural MRI (sMRI) can be used to quantify anatomical alterations in brain regions, including gray matter volume loss, cortical thinning, and morphological abnormalities, such as hippocampus and entorhinal cortex. Functional MRI reflects neuronal activity based on blood oxygenation level-dependent signals. These abnormalities are closely associated with cognitive decline and underscore the value of MRI for detecting and monitoring AD progression (4–6). PET imaging further complements MRI by evaluating cerebral metabolism and Aβ pathology for AD. ^18^F-fluorodeoxyglucose PET (^18^F-FDG PET) measures regional glucose metabolism and reveals characteristic hypometabolism in temporoparietal, posterior cingulate, and prefrontal regions, which correlates strongly with cognitive impairment and supports early diagnosis of AD (5–10). These observations are consistent with previous reports (9, 10). In addition, ^18^F-florbetapir (^18^F-AV45) PET (^18^F-AV45 PET) (11) allows quantitative assessment of cerebral Aβ plaque burden, which facilitates the differentiation of AD from other dementia subtypes and improving diagnostic for AD.

Genetic factors contribute significantly to the onset, pathological progression and clinical phenotypes of sporadic AD. The apolipoprotein E (ApoE) ε4 genotypes allele is the strongest genetic risk factor for sporadic AD, and its effect is dose-dependent on disease susceptibility, with ε4 homozygotes increased lifetime AD risk than heterozygous carriers (12, 13). Beyond increasing disease risk, ApoE ε4 modulates AD-related pathological changes, cerebral structural atrophy and metabolic impairment. For example, neuroimaging studies have demonstrated distinct genotype-specific patterns: ε4 carriers tend to show temporal atrophy and prominent memory impairment, while non-carriers are more prone to frontoparietal degeneration, non-amnestic cognitive deficits and higher tau burden (14, 15). Furthermore, ApoE ε4 has been associated with accelerated longitudinal metabolic decline and alters cerebral amyloid deposition in MCI and AD patients (16). Despite accumulating evidence linking ApoE ε4 to AD-related neurobiological alterations, a multimodal PET-MRI study found no significant differences in regional gray matter volume, Aβ deposition, or glucose metabolism across ApoE ε4 genotypes (17). The authors proposed that neuronal compensation and subtle edema mask early genotype-related brain changes, making them difficult to detect cross-sectional but longitudinal effects. Thus, the precise mechanisms by which ApoE ε4 influence the complex relationship among Aβ pathology, brain structural, metabolic dysfunction, and cognitive deterioration across the AD continuum remain incompletely clarified.

AD induces multi-domain cognitive deficits driven by distinct regional cerebral structural and functional impairments. As the hallmark early manifestation of AD, memory impairment is primarily caused by hippocampal and medial temporal atrophy, which disrupts memory encoding and storage and leads to prominent episodic and semantic memory decline (18). Prefrontal and frontostriatal damage accounts for AD-related executive dysfunction, characterized by defective reasoning, impaired complex-task performance, and impulse dysregulation (19). Left temporal and angular gyrus degeneration disrupts language networks, resulting in progressive anomic and semantic aphasia in AD patients (20). Parieto-occipital atrophy underlies advanced visuospatial deficits, including spatial disorientation and prosopagnosia, while angular gyrus lesions are predominantly responsible for acalculia (21, 22). Advanced AD is also accompanied by progressive attentional, affective, and behavioral abnormalities. Growing neuroimaging and genetic evidence demonstrate that cognitive decline in AD is mediated by both cerebral structural atrophy and glucose metabolic dysfunction, with metabolic dysfunction emerging before detectable atrophy, predicting subsequent cognitive decline, and potentially playing a causal role in cognitive deterioration (23–26). However, the extent to which cerebral hypometabolism and structural atrophy mediate the relationship between AD pathology and cognitive decline remains unclear.

Although previous studies have linked the independent associations of brain structural atrophy and cerebral metabolic decline with cognitive impairment in AD, the interactions among Aβ pathology, structural neurodegeneration, metabolic dysfunction, and cognitive decline remain incompletely understood. In particular, it remains unclear whether region-specific brain atrophy serves as a key mediator linking cerebral hypometabolism to progressive cognitive deterioration. To address this gap, we compared gray matter volume, cerebral glucose metabolism, and ApoE ε4 status between patients with AD and non-AD dementia and examined their associations with cognitive performance. Through this multimodal approach, we aimed at clarifythe associations between cognitive impairment and neuroimaging abnormalities and investigated the mediating role of structural brain atrophy in the relationship between cerebral hypometabolism and AD-related cognitive deterioration.

## 2 Methods

### 2.1 Study subjects

From 2022 to 2025, a total of 182 patients with dementia were initially recruited from the Department of Neurology, the First Affiliated Hospital of Dalian Medical University. After strict screening in accordance with standardized inclusion and exclusion criteria, we enrolled 134 patients meeting all entry criteria with a diagnosis of probable AD for the final analysis. According to the average SUVR of the whole-brain cortex measured by ^18^F-AV45 PET, patients were divided into the Aβ-positive (AD) group (*n* = 92) and the Aβ-negative (non-AD dementia) group (*n* = 42). Of the 134 participants, 71 (53.0%) underwent ApoE genotyping, while 63 (47.0%) did not. Specifically, among the 63 patients without ApoE genotyping, 5 were lost to follow-up, and 58 declined blood sampling for ApoE testing and therefore did not provide additional informed consent for genetic analysis. Based on AV45-PET findings, these 71 patients with ApoE genotyping data were divided into the AD subgroup (*n* = 53) and non-AD subgroup (*n* = 18). The AD subgroup patients were stratified into ApoE ε4 homozygote group (*n* = 9), ApoE ε4 heterozygote group (*n* = 20), and ApoE ε4 non-carrier group (*n* = 24) (Figure 1).

**Figure 1:**
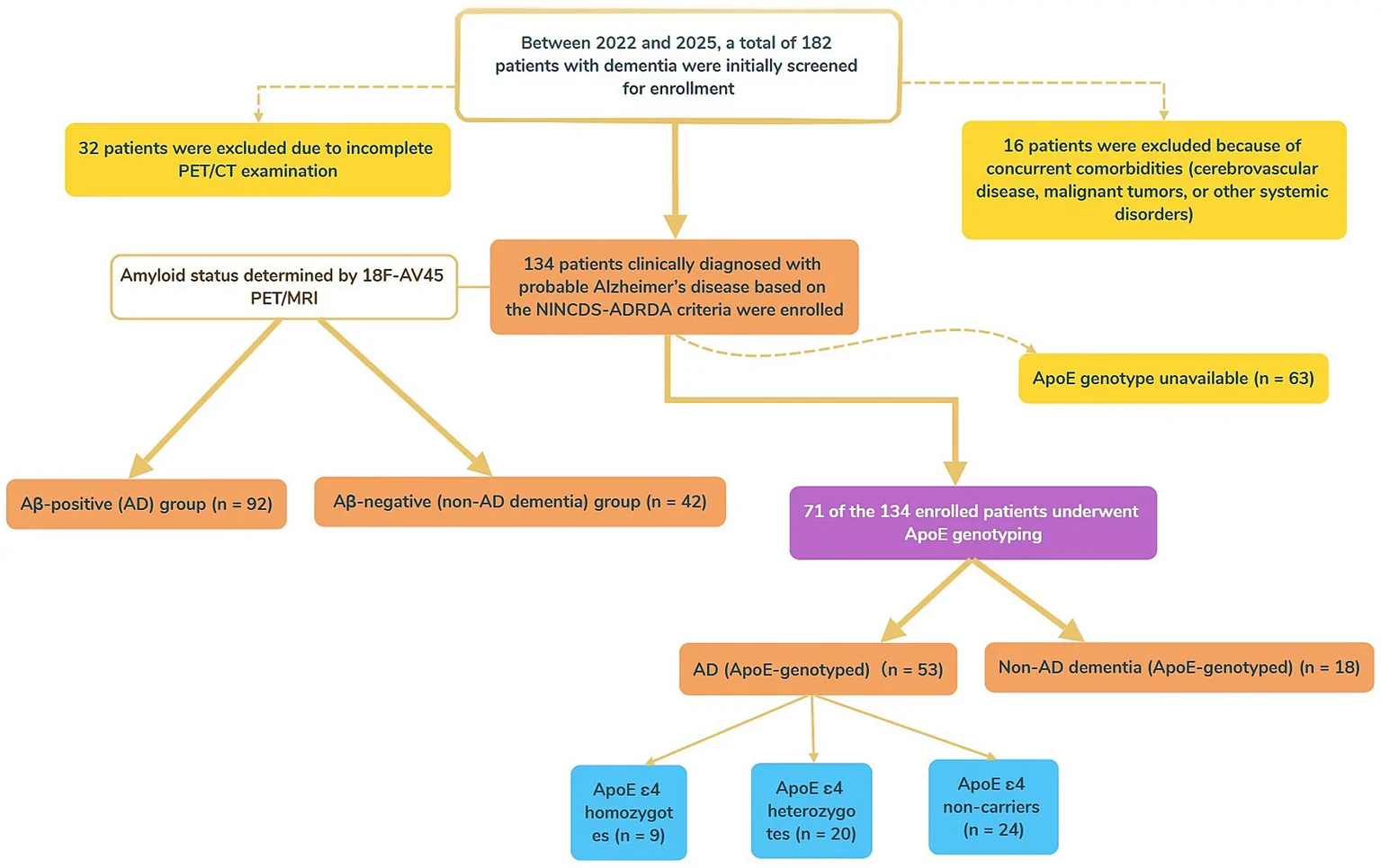
Flowchart of patient enrollment and grouping methods.

#### 2.1.1 Inclusion criteria

(1) Meeting the DSM-IV criteria for dementia (27); (2) Meeting the revised NINCDS-ADRDA criteria for probable AD in 2011 (28); (3) Completing ^18^F-FDG-PET/MRI and ^18^F-AV45-PET/MRI examinations in the hospital.

#### 2.1.2 Exclusion criteria

(1) Inability to cooperate with clinical physical examination and cognitive assessment; (2) Presence of other neurological diseases (e.g., acute cerebrovascular disease, brain tumor), systemic diseases (e.g., HIV infection, syphilis), long-term use of cognitive-impairing drugs or alcoholism, vitamin B deficiency; (3) Severe neurobehavioral abnormalities or mental diseases (e.g., schizophrenia); (4) Severe internal diseases and tumors (e.g., cardiac insufficiency, chronic obstructive pulmonary disease, hepatic and renal insufficiency). All patients provided informed consent for relevant examinations and tests.

### 2.2 Ethics statement

This study was conducted in accordance with the Declaration of Helsinki. The research protocol was reviewed and approved by the Ethics Committee of the First Affiliated Hospital of Dalian Medical University (Approval number: PJ-KS-KY-2025-481). All participants or their legal guardians provided written informed consent prior to enrollment in the study.

### 2.3 Neuropsychological assessment

Five scales were used for neuropsychological assessment, all completed by two neurologists with the title of attending physician or above. Discrepancies were resolved by a chief physician.

#### 2.3.1 MMSE

Developed by Folstein et al. (29), it evaluates orientation, memory, attention, and language, with a total score of 30 points. Scores ≥27 indicate normal cognition, and dementia criteria vary by educational level.

#### 2.3.2 MoCA

Referencing MMSE, it assesses executive function, language, visuospatial ability, etc. (30) Cognitive Function Score: (1) Memory ability: including forward digit span, delayed recall, and orientation scores, with a total score of 12 points. (2) Visuospatial ability: including cube copying and clock drawing test, with a total score of 4 points. (3) Language ability: including animal picture naming and sentence repetition, with a full score of 5 points. (4) Attention: including serial 7 subtraction and digit tapping test, with a total score of 4 points. (5) Executive function: including the trail-making test, backward digit span, word similarity, and fluency, with a total score of 5 points (31).

#### 2.3.3 ADL

A core indicator for measuring independent living quality and functional status, with Katz Index, Barthel Index, and Modified Barthel Index being the most widely used versions in clinical practice (32).

#### 2.3.4 HAMD

Developed in 1959, it assesses depressive symptoms with 24 items. Scores range from normal (0–7) to severe depression (≥35) (33).

#### 2.3.5 HAMA

Also developed in 1959, it evaluates anxiety symptoms with 14 items, scoring from normal (<7) to extremely severe anxiety (≥29) (33).

### 2.4 PET/MRI examinations

All patients underwent PET/MRI examinations (PET/MR model: PMR790, United Imaging Healthcare Co., Ltd., Shanghai), including ^18^F-FDG-PET and ^18^F-AV45-PET examinations, which were used to detect cerebral glucose metabolism and Aβ deposition, respectively. For the ^18^F-FDG-PET examination, patients were required to have a light diet on the day before the examination, fast for 4–6 h before the examination, suspend taking any drugs on the examination day, and have fasting blood glucose was required to be <140 mg/dL. Patients rested for 60 min in a quiet and dark environment, and the contrast agent was injected at a dose of 0.15 mCi/kg. There was no special preparation for the ^18^F-AV45-PET examination, and 10 mCi of the contrast agent was injected. The interval between the two PET/MRI examinations was more than 3 days. PET scanning was performed using a 3D acquisition mode. During PET image acquisition, 3D T1-weighted imaging (3D T1WI) sequence acquisition was obtained synchronously. The MR scanning parameters were as follows: 3D gradient-echo rapid spin preparation sequence (3D-GER-FSP), flip angle = 10°, echo time (TE)/repetition time (TR) = 3.0/7.9 ms, bandwidth = 250 KHz, field of view (FOV) = 256 mm × 230 mm, matrix = 256 × 230. The scanning thickness was 1 mm.

#### 2.4.1 Processing and analysis of MR structural images

Image quality control was implemented for all scans. DICOM files were transformed into NIfTI format, then preprocessing pipelines including skull stripping, gray/white matter tissue segmentation, cortical parcellation using the Desikan-Killiany surface atlas (34), and volumetric subcortical segmentation relying on FreeSurfer’s ASEG probabilistic atlas were conducted sequentially (35). The study included 68 bilateral cortical regions defined and 12 bilateral subcortical regions by the Desikan-Killiany atlas. Bilateral subcortical structures including thalamus, Caudate, Putamen, Pallidum, Hippocampus, and Amygdala as the regions of interest.

#### 2.4.2 Processing and analysis of PET images

PET volumes were rigidly coregistered to individual T1-weighted MRI using FreeSurfer spmregister, followed by spatial normalization of native T1 images to the MNI standard template space (36). ROIs were defined and PET uptake was quantified in standard MNI space based on the Desikan-Killiany cortical atlas and ASEG parcellation. Geometric transfer matrix-based partial volume correction was performed using PETSurfer (37). SUVRs were calculated by dividing mean uptake in each target ROI by mean uptake in a reference region, using the whole cerebellum for ^18^F-AV45 PET (11) and the pons for ^18^F-FDG PET (38).

The average SUVR of the whole-brain cortex measured by AV45-PET was calculated using the formula: SUVRmean = ∑(SUVRi*Volumei)/∑(Volumei). Whole-brain cortex average SUVR >1.10 was classified as amyloid-positive (39).

### 2.5 ApoE genotyping

Peripheral blood (3-5 mL) was collected from 71 patients, stored at 2–8 °C, and sent for testing within 8 h. The experimental steps were as follows: (1) DNA extraction: Using a whole blood DNA extraction kit. (2) PCR reaction preparation: Thawing reagents (2*Vazyme Lamp master mix, ddH2O, primers, DNA), preparing the reaction system with ApoE primer working solution (final concentration 5 μM), and setting up control and blank control groups. (3) PCR amplification: Oscillating, centrifuging, and running the amplification program on a PCR instrument. Products were stored at 2–8 °C if not electrophoresed immediately. (4) Electrophoresis: Preparing 2% agarose gel with Gold View nucleic acid stain, loading 3-5 μL of PCR products, and performing electrophoresis at 150-200 V for 8-15 min. (5) Imaging and sequencing: Imaging with a Bio-Rad ChemiDoc XRS + gel imaging system, followed by enzymatic digestion of PCR products, sequencing with M13-F and M13-R primers, magnetic bead purification, and capillary electrophoresis detection with a 3,730 Genetic Analyzer. (6) Genotype analysis: Using Mutation Surveyor software to compare with the ApoE gene sequence in the NCBI database, classifying genotypes as E2/E2, E3/E3, E4/E4, E2/E3, E2/E4, and E3/E4.

### 2.6 Statistical analysis

Analysis of covariance (ANCOVA) was applied to compare regional GMV and FDG metabolism between AD and non-AD group. Group was set as the main independent variable, while age, sex, education level, disease duration, and estimated total intracranial volume (eTIV) were included as covariates to eliminate the confounding effects of individual cranial size differences on GMV measurements. The group main effect was evaluated via the *F*-test, and partial eta squared was calculated to quantify the effect size. All statistical analyses were performed with multiple comparison corrections using the Benjamini–Hochberg false discovery rate (FDR) method, and an FDR-adjusted *p* < 0.05 was defined as the threshold for statistical significance unless otherwise specified.

Separated multiple linear regression analyses were conducted in the AD and non-AD groups to investigate the associations of Aβ deposition with cognitive performance, regional GMV, and FDG uptake. In each model, global Aβ deposition served as the independent variable, while cognitive scores, GMV, and regional FDG uptake were separately treated as dependent variables. All regression models were adjusted for age, sex, education, and other predefined covariates. Model outcomes were reported as standardized coefficient (*β*), *t*-value, 95% confidence interval (CI), *p* value, FDR-P value, coefficient of determination (*R*^2^), and adjusted *R*^2^.

Among 53 patients with AD, subgroup analyses based on ApoE genotypes (homozygotes, heterozygotes, and non-carriers) were performed to compare cognitive scores, regional GMV, FDG SUVR, and AV45 SUVR. Covariate-adjusted linear models were constructed with ApoE genotype as the categorical independent variable, adjusting for age, sex, education, and other predefined confounding factors. Results were summarized using *F*-statistic, *p* value, FDR-P value.

Within the AD group, partial correlation analyses (adjusted for age, sex, disease duration, education, and eTIV) were used to explore the correlations between five domain-specific cognitive scores and regional GMV as well as FDG metabolism. The adjusted partial correlation coefficient (rₚ), *p* value and FDR-P value were calculated for all correlation analyses.

#### 2.6.1 Mediation analyses

Regression-based mediation analyses were conducted in 92 patients with Alzheimer’s disease (AD) using R. Separate mediation models were fitted to examine whether regional GMV mediated the associations between regional FDG metabolism and visuospatial ability, executive function, and language ability. In each model, regional FDG metabolism was specified as the independent variable (X), the corresponding regional GMV as the mediator (M), and the relevant cognitive measure as the outcome (Y). All models were adjusted for age, sex, disease duration, years of education, and eTIV. The mediator model estimated the association between regional FDG metabolism and regional GMV (X → M). The outcome model estimated the association between regional GMV and cognitive performance (M → Y), while also estimating the direct effect of regional FDG metabolism on cognition. The indirect effect was calculated as the product of the X → M and M → Y path coefficients. Indirect effects were estimated using 5,000 bootstrap resamples, with HC3 heteroscedasticity-consistent standard errors used for inference. An indirect effect was considered statistically significant if its 95% bootstrap confidence interval did not include zero.

To assess the robustness of the hypothesized model direction, reverse mediation analyses were conducted by specifying regional GMV as the independent variable, regional FDG metabolism as the mediator, and the corresponding cognitive measure as the outcome. The same covariates and statistical procedures were applied. These reverse mediation analyses were treated as sensitivity analyses and were not intended to establish temporal or causal direction. Given the cross-sectional study design, all mediation effects were interpreted as statistical associations rather than causal effects. Therefore, the temporal sequence from altered FDG metabolism to reduced regional GMV and cognitive impairment could not be determined.

#### 2.6.2 ApoE-adjusted mediation analyses

To further evaluate the robustness of the mediation models, ApoE-adjusted mediation analyses were conducted in a subgroup of 53 patients with AD with complete ApoE genotyping data. These analyses examined the three hypothesized mediation models involving the pathway from regional GMV to regional FDG metabolism to cognitive performance. ApoE ε4 carrier status was included as an additional covariate, together with age, sex, disease duration, years of education, and eTIV. ApoE ε4 carrier status was coded as 1 for carriers and 0 for non-carriers. The same regression-based mediation framework, 5,000 bootstrap resamples, HC3 heteroscedasticity-consistent standard errors, and 95% bootstrap confidence intervals were used.

#### 2.6.3 Multiple-imputation mediation analyses

Because ApoE genotyping was not performed in 39 of the 92 patients with AD, multiple imputation was used to reduce potential bias and loss of statistical power due to missing data. Fifty imputed datasets were generated, and the same mediation models were fitted separately in each dataset, with age, sex, disease duration, years of education, eTIV, and ApoE ε4 carrier status included as covariates. Indirect effects were estimated using 5,000 bootstrap resamples, with HC3 heteroscedasticity-consistent standard errors. Regression coefficients, standard errors, and mediation effect estimates were pooled across the imputed datasets according to Rubin’s rules. The statistical significance of the indirect effects was assessed using the pooled 95% confidence intervals; an indirect effect was considered statistically significant when the corresponding confidence interval did not include zero.

All primary and reverse mediation models included age, sex, disease duration, years of education, and eTIV as covariates. These covariates were selected *a priori* because they may be associated with neuroimaging measures and cognitive performance, while eTIV was included to account for individual differences in intracranial size in analyses involving regional GMV. The ApoE-adjusted mediation models additionally included ApoE ε4 carrier status, given its established role as a genetic risk factor for AD and its potential associations with neurodegeneration, cerebral glucose metabolism, and cognitive impairment. The same covariate structure was used in the multiple-imputation analyses.

## 3 Results

### 3.1 Clinical and neuropsychological characteristics

The AD group consisted of 92 patients (35 males and 57 females) with a mean age of 69.78 ± 8.01 years, a median disease duration of 3 years [interquartile range (IQR), 2.0–5.0], and a median education level of 9 years (IQR, 9.0–13.5).

**Figure 2:**
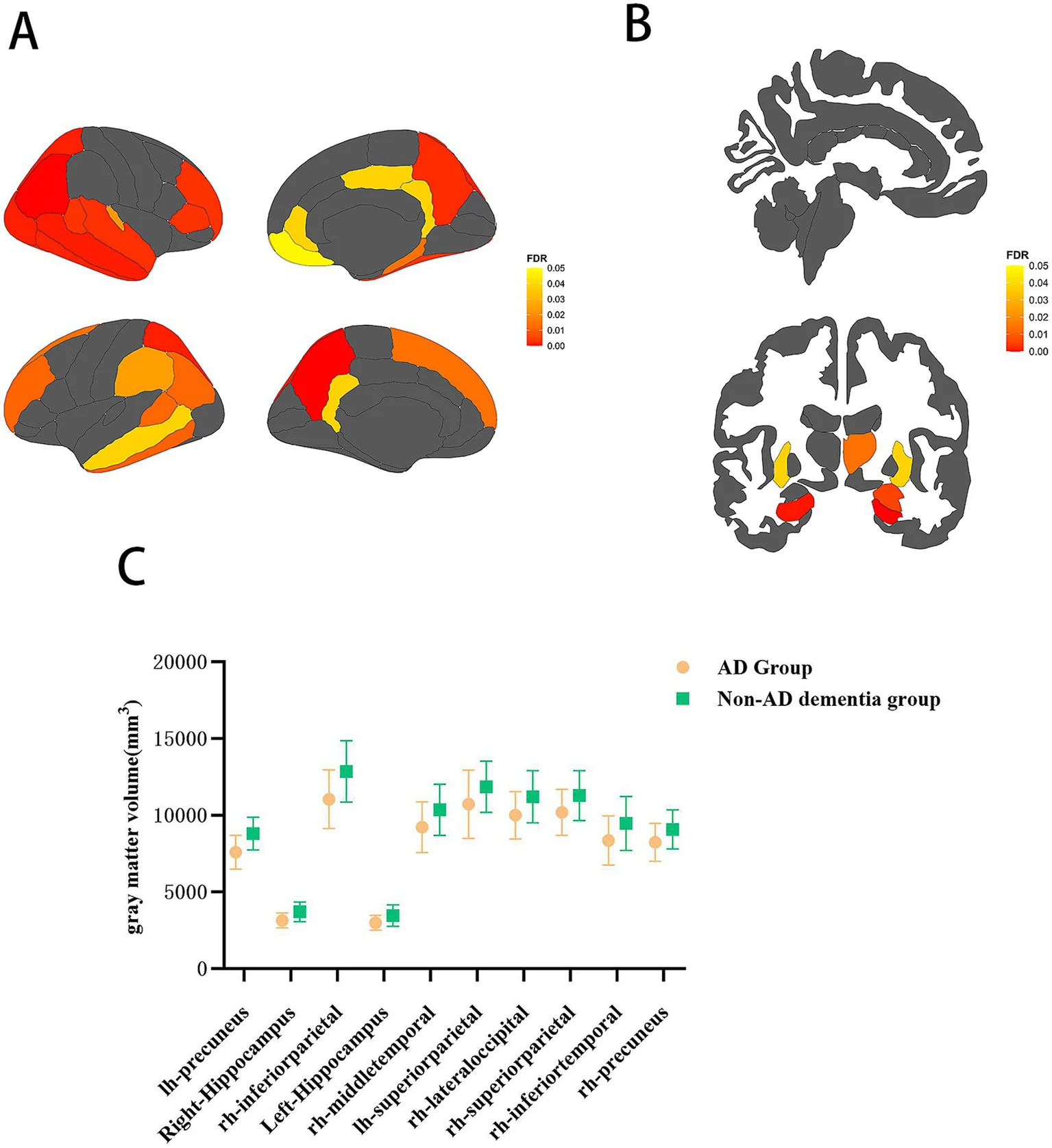
Gray matter volume differences between AD and non-AD dementia groups. **(A,B)** Cortical **(A)** and subcortical **(B)** regions with significant volumetric differences (FDR-P < 0.05); Warmer colors denote smaller FDR-P (more significant), regions with no color (gray) show no significant difference between groups. The nucleus accumbens cannot be visualized in 2D representations. **(C)** Boxplots of top 10 brain regions showing the most significant group differences, ranked by FDR-corrected *p* values.

The non-AD dementia group included 42 patients (19 males and 23 females) with a mean age of 68.62 ± 6.10 years, a median disease duration of 4.0 years (IQR, 2.0–5.0), and a median educational level of 12 years (IQR, 9.0–15.0). After adjustment for age and sex, the two groups differed significantly in years of education, with the AD group having significantly fewer years of education than the non-AD dementia group (*p* = 0.023). After adjustment for age, sex, years of education, and disease duration, no significant between-group differences were observed in MMSE, MoCA, ADL, HAMA, HAMD, or ApoE ε4 carrier status (all *p* > 0.05; Table 1).

**Table 1:** Comparison of clinical data and neuropsychological scale scores between the AD group and the non-AD dementia group.

Indicators	AD group (*n* = 92)	Non-AD dementia group (*n* = 42)	*p* value
Age (years, x ± s)	69.78 ± 8.01	68.62 ± 6.10	0.404
Male [*n* (%)]	35 (38.04)	14 (33.33)	0.433
Disease duration (years)	3.0(2.0, 5.0)	4.00 (2.00, 5.00)	0.388
Educational years (years)	9.00 (9.00, 13.50)	12.00 (9.00, 15.00)	0.023*
MMSE (points)	17.50 (13.00, 22.00)	20.5 (11.00, 25.00)	0.088
MoCA (points, x ± s)	12.21 ± 0.61	13.86 ± 0.99	0.088
HAMA (points)	10.00(6.00, 14.00)	10.50(6.00, 15.00)	0.522
HAMD (points)	9.00 (6.00, 14.00)	11.00 (7.00, 14.00)	0.536
ADL (points)	24 (18.00, 34.00)	3.34 ± 1.28	0.537
ApoE ε3/ε4 or ε2/ε4[n]	20	5	0.182
ApoE ε4/ε4[n]	9	1	-
ApoE ε3/ε3 or ε2/ε3[n]	24	12	-

MMSE, Mini-Mental State Examination; MoCA, Montreal Cognitive Assessment; ADL, Activities of Daily Living Scale; HAMA, Hamilton Anxiety Scale; HAMD, Hamilton Depression Scale. *p < 0.05 indicates a statistically significant difference.

### 3.2 Regional gray matter volume differences between AD and non-AD dementia

After adjusting for covariates including age, gender, disease duration, years of education, and eTIV, compared with the non-AD dementia group, patients with AD showed significantly lower volume in 34 brain regions (FDR-P < 0.05) (Table 2; Figures 2A,B). Based on FDR corrected *p* values, The top 10 brain GMV with the most significant differences are left precuneus, right hippocampus, right inferior parietal lobule (rIPL), left hippocampus, right middle temporal gyrus, left superior parietal lobule, right lateral occipital lobe, right superior parietal lobule, right inferior temporal gyrus, right precuneus, with left precuneus and right hippocampus showing the most significant alterations (Figure 2C). These findings suggested more widespread structural brain atrophy in AD relative to non-AD dementia. Full results of the group comparisons across all brain regions are presented in Supplementary Table S1.

**Table 2:** Brain regions with differences in gray matter volume between the AD group and the Non-AD dementia group.

Gray matter volume (mm^3^)	AD group (*n* = 92)	Non-AD dementia group (*n* = 42)	*F* value	ηₚ^2^	*p* value	FDR-P value
lh-precuneus	8240.20 ± 1237.32	8803.12 ± 1069.21	35.398	0.217	2.409e-08	2.072e-06***
Right-Hippocampus	3144.16 ± 482.13	3703.22 ± 641.56	28.498	0.182	4.134e-07	1.777e-05***
rh-inferiorparietal	11045.87 ± 1905.20	12850.55 ± 2000.25	26.572	0.172	9.377e-07	2.688e-05***
Left-Hippocampus	2991.29 ± 482.76	3461.05 ± 696.58	18.064	0.124	4.083e-05	0.001***
rh-middletemporal	9232.10 ± 1649.16	10360.83 ± 1665.00	16.847	0.116	0.0001	0.001***
lh-superiorparietal	10719.41 ± 2227.94	11857.86 ± 1661.64	15.736	0.109	0.0001	0.001***
rh-lateraloccipital	10002.79 ± 1542.85	11204.50 ± 1703.33	15.509	0.108	0.0001	0.001***
rh-superiorparietal	10185.04 ± 1498.96	11284.60 ± 1627.79	15.809	0.110	0.0002	0.001***
rh-inferiortemporal	8360.45 ± 1598.35	9467.33 ± 1763.85	14.714	0.103	0.0003	0.001***
rh-precuneus	8240.20 ± 1237.36	9080.73 ± 1272.55	13.886	0.098	0.0003	0.002**
rh-superiortemporal	9539.17 ± 1289.37	10503.93 ± 1495.77	13.289	0.094	0.0004	0.003**
rh-rostralmiddlefrontal	12281.45 ± 2046.96	13726.31 ± 2160.58	13.004	0.092	0.0005	0.003**
rh-parstriangularis	3469.17 ± 584.30	3871.51 ± 608.40	12.789	0.091	0.0006	0.003**
rh-bankssts	1547.30 ± 298.98	1747.31 ± 351.88	12.551	0.089	0.0007	0.003**
rh-fusiform	7718.84 ± 1413.71	8700.71 ± 1583.81	12.156	0.087	0.0010	0.004**
Right-Amygdala	1398.72 ± 310.53	1619.04 ± 381.37	11.346	0.081	0.002	0.005**
lh-inferiortemporal	8614.19 ± 1481.91	9540.19 ± 2177.07	9.924	0.072	0.002	0.010**
lh-rostralmiddlefrontal	11762.30 ± 1765.97	12768.88 ± 1621.67	9.621	0.070	0.003	0.011*
lh-bankssts	1726.60 ± 373.95	1942.57 ± 394.25	9.492	0.068	0.003	0.012*
lh-inferiorparietal	9615.00 ± 1756.53	10662.53 ± 1687.78	9.357	0.068	0.004	0.012*
lh-superiorfrontal	18279.40 ± 2227.94	19489.19 ± 2653.24	9.283	0.064	0.004	0.014*
rh-parahippocampal	1560.90 ± 260.22	1719.24 ± 335.43	8.687	0.064	0.004	0.014*
Right-Thalamus	6292.74 ± 830.84	6782.86 ± 881.62	8.717	0.065	0.007	0.014*
rh-transversetemporal	758.53 ± 147.57	844.24 ± 168.10	8.842	0.056	0.008	0.022*
lh-supramarginal	8763.58 ± 1730.05	9511.83 ± 1363.41	7.780	0.054	0.012	0.025*
rh-isthmuscingulate	2105.63 ± 352.81	2286.83 ± 417.89	7.583	0.048	0.016	0.038*
lh-isthmuscingulate	2258.72 ± 454.96	2492.93 ± 471.49	7.331	0.045	0.015	0.038*
lh-middletemporal	8537.28 ± 1511.17	9269.64 ± 2016.78	6.435	0.045	0.015	0.038*
rh-posteriorcingulate	2543.78 ± 531.10	2796.26 ± 508.96	6.021	0.046	0.014	0.038*
rh-rostralanteriorcingulate	1565.28 ± 437.92	1761.95 ± 373.95	6.032	0.046	0.016	0.038*
rh-frontalpole	1013.83 ± 186.54	1106.88 ± 193.07	6.108	0.045	0.013	0.038*
Left-Putamen	4093.37 ± 620.26	4422.58 ± 833.11	6.183	0.047	0.014	0.038*
Right-Putamen	4320.25 ± 756.59	4675.07 ± 796.18	6.020	0.047	0.013	0.038
rh-medialorbitofrontal	4695.65 ± 565.62	4961.60 ± 647.80	6.352	0.042	0.020	0.047

*FDR-P ≤ 0.05, statistically significant difference; **FDR-P ≤ 0.01; ***FDR-P ≤ 0.001, highly statistically significant difference.

**Figure 3:**
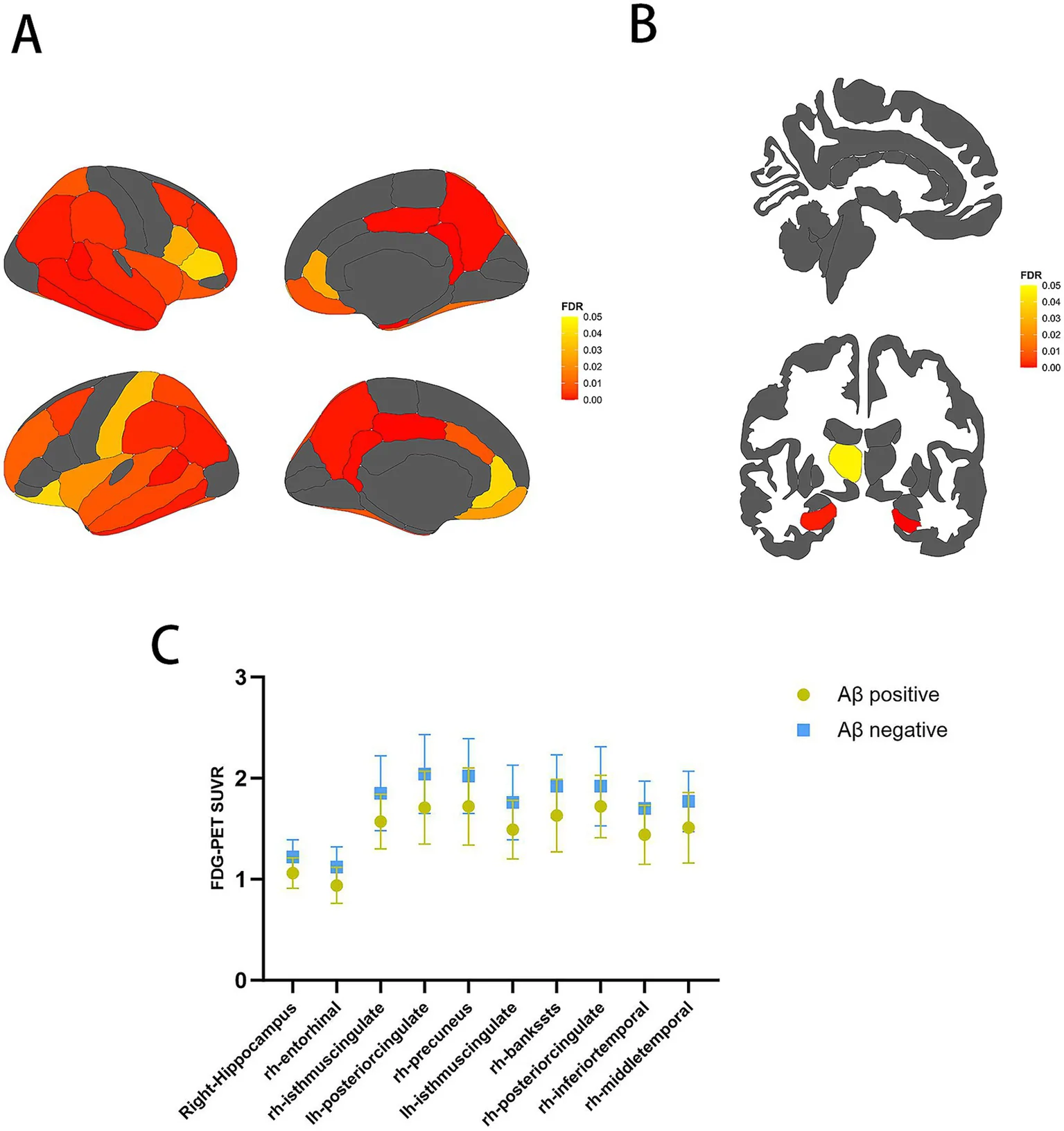
Regional cerebral glucose metabolism differences between AD and non-AD dementia groups. **(A,B)** Significant cortical **(A)** and subcortical **(B)** metabolic alterations (FDR-P < 0.05). Warmer colors denote lower FDR-P (higher significance); Gray indicates no intergroup difference. The nucleus accumbens cannot be visualized in 2D representations. **(C)** Boxplots of top 10 brain regions showing the most significant group differences, ranked by FDR-corrected *p* values.

### 3.3 Regional ^18^F-FDG metabolism differences between AD and non-AD dementia

After adjusting for covariates including age, gender, disease duration, years of education, comparison of ^18^F-FDG-PET SUVR values revealed significantly lower glucose metabolism in 43 brain regions between AD and non-AD dementia patients (FDR-*p* < 0.05) (Table 3; Figures 3A,B). Ranked by FDR corrected *p* values, the top 10 brain regions with the most significant differences are right hippocampus, right entorhinal cortex, right isthmus cingulate, left posterior cingulate, right precuneus, left isthmus cingulate, right banks of the superior temporal sulcus, right posterior cingulate, right inferior temporal gyrus, right middle temporal gyrus (Figure 3C). Full results of the group comparisons across all brain regions are presented in Supplementary Table S2.

**Table 3:** Brain regions with differences in ^18^F-FDG-PET SUVR values between the AD Group and the non-AD dementia group.

Brain region	AD group (*n* = 92)	Non-AD dementia group (*n* = 42)	*F* value	ηₚ^2^	*p* value	FDR-P value
Right-Hippocampus	1.06 ± 0.15	1.22 ± 0.17	27.853	0.179	5.431e-07	4.997e-05***
rh-entorhinal	0.94 ± 0.18	1.12 ± 0.20	25.574	0.167	1.440e-06	6.625e-05***
rh-isthmuscingulate	1.57 ± 0.27	1.85 ± 0.37	22.550	0.150	5.401e-06	0.0002***
lh-posteriorcingulate	1.70 ± 0.36	2.04 ± 0.39	21.573	0.144	8.334e-06	0.0002***
rh-precuneus	1.72 ± 0.38	2.02 ± 0.37	19.269	0.131	2.352e-05	0.0004***
lh-isthmuscingulate	1.49 ± 0.29	1.76 ± 0.37	18.469	0.126	3.389e-05	0.0005***
rh-bankssts	1.63 ± 0.36	1.92 ± 0.31	18.289	0.125	3.682e-05	0.0005***
rh-posteriorcingulate	1.72 ± 0.31	1.94 ± 0.39	18.133	0.124	3.956e-05	0.0005***
rh-inferiortemporal	1.44 ± 0.29	1.70 ± 0.27	17.415	0.120	5.508e-05	0.0006***
rh-middletemporal	1.51 ± 0.35	1.77 ± 0.30	16.747	0.116	7.511e-05	0.0007***
lh-bankssts	1.62 ± 0.38	1.91 ± 0.32	16.501	0.114	8.424e-05	0.0007***
lh-precuneus	1.72 ± 0.38	2.02 ± 0.37	15.557	0.108	0.0001	0.001***
Left-Hippocampus	1.01 ± 0.15	1.13 ± 0.17	14.627	0.103	0.0002	0.001***
lh-inferiorparietal	1.59 ± 0.41	1.90 ± 0.42	14.687	0.103	0.0002	0.001***
lh-inferiortemporal	1.41 ± 0.31	1.67 ± 0.36	14.800	0.104	0.0002	0.001***
rh-inferiorparietal	1.57 ± 0.38	1.87 ± 0.42	14.869	0.104	0.0002	0.001***
rh-superiortemporal	1.50 ± 0.26	1.69 ± 0.32	13.266	0.094	0.0004	0.002**
lh-supramarginal	1.59 ± 0.36	1.84 ± 0.33	13.078	0.093	0.0004	0.002**
rh-rostralmiddlefrontal	1.81 ± 0.89	1.92 ± 0.25	12.801	0.091	0.0005	0.002**
rh-caudalmiddlefrontal	1.81 ± 0.39	2.09 ± 0.43	12.384	0.088	0.0006	0.003**
rh-supramarginal	1.67 ± 0.31	1.83 ± 0.35	12.126	0.087	0.0007	0.003**
lh-caudalmiddlefrontal	1.83 ± 0.37	2.08 ± 0.41	11.432	0.082	0.001	0.004**
lh-superiorparietal	1.67 ± 0.31	1.93 ± 0.37	10.705	0.078	0.001	0.005**
lh-middletemporal	1.49 ± 0.34	1.69 ± 0.32	10.439	0.077	0.002	0.006**
rh-insula	1.36 ± 0.30	1.57 ± 0.39	10.315	0.075	0.002	0.006**
rh-medialorbitofrontal	1.55 ± 0.25	1.71 ± 0.27	9.706	0.075	0.002	0.008**
lh-caudalanteriorcingulate	1.40 ± 0.19	1.53 ± 0.23	9.415	0.070	0.003	0.008**
lh-fusiform	1.50 ± 0.29	1.69 ± 0.34	9.324	0.069	0.003	0.008**
rh-lateralorbitofrontal	1.64 ± 0.26	1.80 ± 0.28	9.438	0.068	0.003	0.008**
rh-superiorparietal	1.66 ± 0.35	1.89 ± 0.38	9.352	0.069	0.003	0.008**
lh-superiortemporal	1.49 ± 0.69	1.66 ± 0.31	9.208	0.068	0.003	0.008**
lh-rostralmiddlefrontal	1.86 ± 0.40	2.08 ± 0.78	8.800	0.067	0.004	0.010*
rh-fusiform	1.53 ± 0.27	1.70 ± 0.32	8.404	0.064	0.004	0.012*
lh-insula	1.38 ± 0.30	1.57 ± 0.47	7.205	0.062	0.008	0.021*
lh-medialorbitofrontal	1.53 ± 0.24	1.67 ± 0.33	6.906	0.061	0.010	0.024*
rh-rostralanteriorcingulate	1.40 ± 0.25	1.54 ± 0.33	6.662	0.053	0.011	0.027*
rh-parsopercularis	1.76 ± 0.32	1.92 ± 0.36	6.511	0.051	0.012	0.028*
lh-postcentral	1.78 ± 0.32	1.93 ± 0.27	6.271	0.049	0.014	0.031*
rh-frontalpole	1.79 ± 0.38	1.96 ± 0.41	6.232	0.048	0.014	0.031*
lh-lateralorbitofrontal	1.68 ± 0.26	1.80 ± 0.33	5.806	0.047	0.017	0.038*
lh-rostralanteriorcingulate	1.40 ± 0.25	1.54 ± 0.33	5.693	0.046	0.018	0.039*
rh-parstriangularis	1.83 ± 0.36	1.99 ± 0.35	5.722	0.043	0.018	0.039*
Left-Thalamus	1.48 ± 0.18	1.57 ± 0.19	5.272	0.043	0.023	0.047*

*FDR-P ≤ 0.05, statistically significant difference; **FDR-P ≤ 0.01; ***FDR-P ≤ 0.001; ****FDR-P ≤ 0.0001, highly statistically significant difference.

Among the significant FDG-PET findings, 23 regions also exhibited significant gray matter volume reduction. These overlapping regions primarily included the bilateral hippocampus, bilateral precuneus, multiple temporal subregions, various cingulate cortex subareas, parietal lobe regions, and prefrontal cortex subregions.

### 3.4 Correlation between whole-brain aβ deposition and regional gray matter volume, metabolism, cognitive function and ApoE ε4 gene between AD and non-AD dementia

Following adjustment for age, sex, disease duration and eTIV, we did not observe significant associations between global Aβ deposition and regional GMV or ^18^F-FDG metabolism in either the AD or non-AD dementia group in this cross-sectional study (all FDR-corrected *p* > 0.05). After further adjustment for age, sex, education, and disease duration, no significant associations were observed between global Aβ burden and cognitive or emotional scale scores within the AD group. In contrast, in the non-AD dementia group, we observed positive associations between global Aβ burden and MMSE scores (*β* = 0.459, *p* = 0.042) and MoCA scores (*β* = 0.423, *p* = 0.043) (Supplementary Tables S3–S7).

### 3.5 Exploratory analysis of ApoE ε4-related differences in cognitive performance and brain imaging biomarkers in AD

ApoE genotype-related analyses of cognitive performance and brain imaging biomarkers are presented in the Supplementary materials. These analyses were exploratory and hypothesis-generating; given the small subgroup sizes, particularly the ε4 homozygote group (*n* = 9), the results are not suitable for formal statistical comparisons or inference (The full results are presented in the Supplementary Tables S8–S11).

### 3.6 Correlation between cognitive domains and regional gray matter volume/metabolism

#### 3.6.1 Correlation with gray matter volume

Attention was associated with 33 brain regions, predominantly within the frontal and parietal lobes, with the strongest correlation in the right supramarginal gyrus (*r* = 0.38, FDR-P = 0.006) and precentral gyrus (*r* = 0.38, FDR-P = 0.006). Visuospatial ability showed strongest overall correlation with GMV, with the rIPL as the core region (*r* = 0.54, FDR-P < 0.0001), involving the parietal lobe and occipitotemporal junction. Executive function showed strong correlations with brain regions primarily located in the limbic system. The right amygdala (rAmyg) (*r* = 0.47, FDR-P = 0.004) and left amygdala (*r* = 0.44, FDR-P = 0.001) served as the core associated regions, followed by the left precuneus (*r* = 0.40, FDR-P = 0.003). Language ability exhibited strongest correlation with the left lateral orbitofrontal cortex (*r* = 0.39, FDR-P = 0.007), followed by right caudal middle frontal (*r* = 0.37, FDR-P = 0.006) and left superior temporal cortex (lSTG) (*r* = 0.36, FDR-P = 0.007). No regional gray matter volumes were significantly correlated with memory performance (Supplementary Table S16).

#### 3.6.2 Correlation with glucose metabolism

Executive function was positively correlated with glucose metabolic levels in the left inferior parietal lobule (lIPL) (*r* = 0.35, FDR-P = 0.015), left transverse temporal cortex (*r* = 0.34, FDR-P = 0.017). Brain regions associated with visuospatial ability were predominantly distributed in bilateral temporal cortices, alongside bilateral inferior parietal cortices and the left pars opercularis, the region with the strongest correlation was the right posterior superior temporal gyrus (rSTG) (*r* = 0.443, FDR-P < 0.0001). Language ability was significantly correlated with metabolic activity in the left superior temporal gyrus (lSTG) (*r* = 0.41, FDR-P = 0.003) and left pars opercularis cortex (*r* = 0.40, FDR-P = 0.009). Memory was associated with metabolism in two regions, including the right posterior cingulate cortex (*r* = 0.319, FDR-P = 0.045) and left posterior cingulate cortex (*r* = 0.329, FDR-P = 0.042). No significant correlations were observed between attention and regional cerebral glucose metabolism (Supplementary Table S17).

### 3.7 Mediation effect models of cognitive function

#### 3.7.1 Screening of target brain regions

Based on the three core prerequisites of mediation effect (significant X → M, M → Y, and X → Y correlations), target brain regions for each cognitive domain were screened. Visuospatial ability, executive function, and language ability met the prerequisites, while memory and attention did not (Supplementary Table S20).

#### 3.7.2 Primary mediation analyses in AD

Mediation analyses were conducted in 92 patients with AD to investigate the potential mediating role of GMV in the association between FDG metabolism and cognitive function. In terms of visuospatial ability, rSTG FDG metabolism positively predicted rIPL GMV (*β* = 0.4023, *p* < 0.001). After adjusting for rSTG metabolism, increased rIPL GMV further independently promoted visuospatial performance (*β* = 2.8783, *p* = 0.0012). Both the total effect and direct effect of rSTG FDG metabolism on visuospatial ability were significant, and rIPL GMV exhibited a significant partial mediating effect, accounting for 40.36% of the total effect (Figure 4A; Table 4).

**Figure 4:**
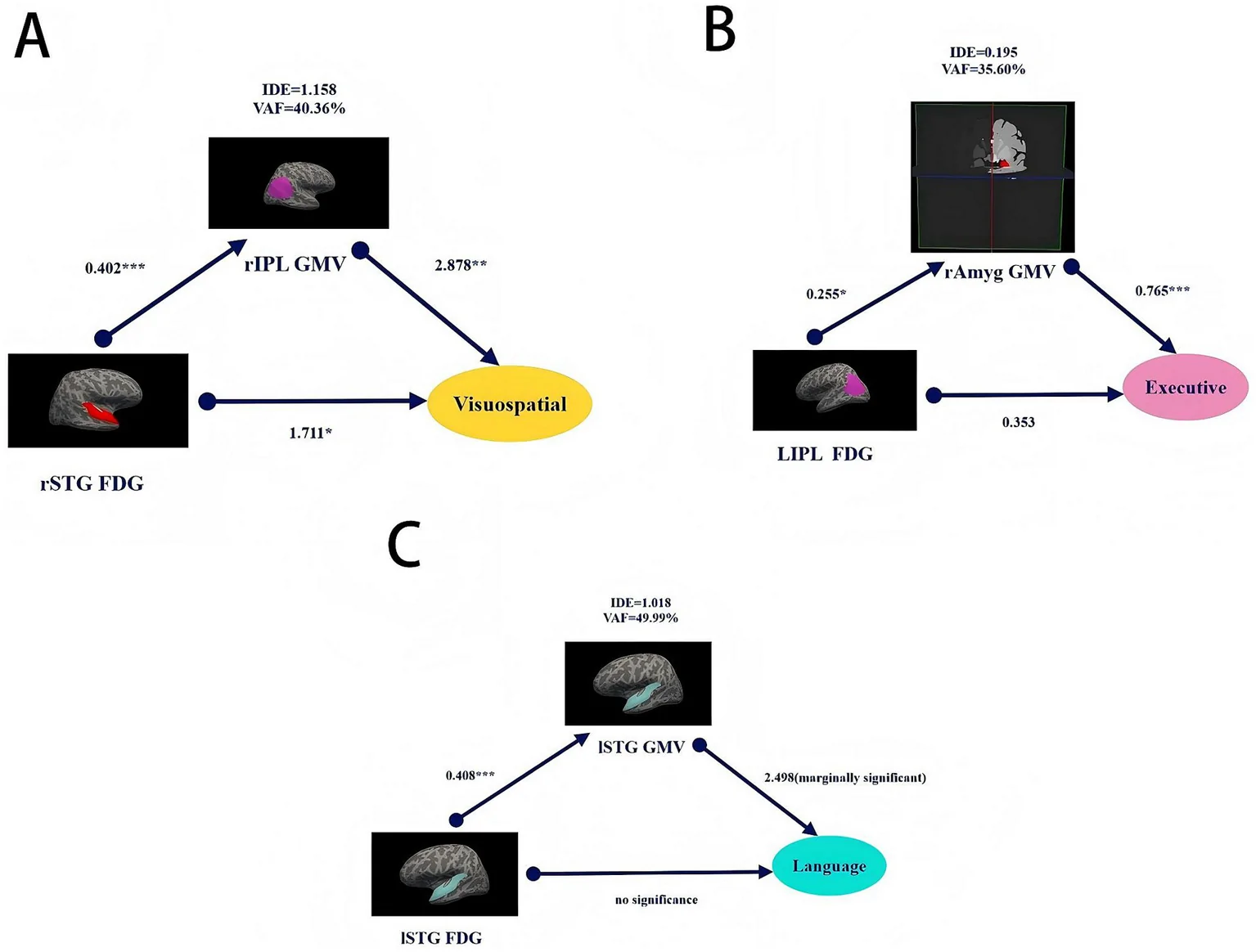
Mediation models of cognitive function mediated by regional cerebral metabolism and gray matter volume (GMV). **(A)** Visuospatial cognitive function: *X* = rSTG ^18^F-FDG metabolism; M = rIPL GMV; Y = visuospatial score. X → M: *β* = 0.402, *p* < 0.001; M → Y: *β* = 2.878, *p* < 0.01; direct X → Y: *β* = 1.711, *p* < 0.05. **(B)** Executive cognitive function: X = lIPL ^18^F-FDG metabolism; M = rAmyg GMV; Y = executive score. X → M: *β* = 0.255, *p* < 0.05; M → Y: *β* = 0.765, *p* < 0.001; direct X → Y: *β* = 0.353, *p* > 0.05 (n.s.). **(C)** Language cognitive function: X = lSTG ^18^F-FDG metabolism; M = lSTG GMV; Y = language score. *p < 0.05, **p < 0.01, ***p < 0.001.

**Table 4:** Primary mediation analyses in AD.

Cognitive function	Path relationship	X → M path (*β*, *p* value)	M → Y path (*β*, *p* value)	Total effect (*β*, *p* value)	Direct effect (*β*, *p* value)	Mediation effect (effect value, 95% CI)	Proportion of mediation effect	Model fit (*R*^2^, *p* value)
Visuospatial ability	Right posterior superior temporal gyrus FDG (X) → right inferior parietal lobule volume (M) → visuospatial score (Y)	0.519, *p* < 0.001	0.396, *p* < 0.001	0.509, *p* < 0.001	0.304, *p* = 0.030	1.158,(0.520, 1.788)	40.36%	0.397, *p* < 0.001
Executive function	Left inferior parietal lobule FDG (X) → right amygdala volume (M) → executive score (Y)	0.255, *p* = 0.045	0.429, *p* < 0.001	0.548, *p* = 0.016	0.353, *p* = 0.096	0.110,(0.015, 0.221)	35.60%	0.282, *p* < 0.001
Language ability	Left superior temporal gyrus FDG (X) → left superior temporal gyrus volume (M) → language score (Y)	0.500, *p* < 0.001	0.263, *p* = 0.005	0.262, *p* = 0.012	Not significant	0.132,(0.005, 0.299)	49.99%	0.135, *p* = 0.014

*β* is the standardized coefficient; CI is the Bootstrap 95% confidence interval; FDG is fluorodeoxyglucose; X is the independent variable (*Z*-score of brain region FDG metabolic value); M is the mediating variable (*Z*-score of brain region gray matter volume); Y is the dependent variable (cognitive function score).

For executive function, lIPL FDG metabolism was positively associated with rAmyg GMV (*β* = 0.2553, *p* = 0.0454). Elevated rAmyg GMV significantly predicted better executive function after controlling for lIPL metabolism (*β* = 0.7646, *p* < 0.001). The total effect of lIPL FDG metabolism on executive function was significant, while the direct effect showed marginal significance. rAmyg GMV played a significant partial mediating role, explaining 35.6% of the total effect (Figure 4B; Table 4).

Regarding language ability, lSTG FDG metabolism positively predicted lSTG GMV (*β* = 0.4075, *p* = 0.0001). lSTG GMV showed a marginally positive predictive effect on language ability after adjustment. The total effect of lSTG FDG metabolism on language ability was significant, whereas the direct effect was not significant. lSTG GMV exerted a significant mediating effect, accounting for 49.99% of the total effect and suggesting a marginal partial mediating mechanism underlying language function (Figure 4C; Table 4).

#### 3.7.3 Reverse mediation sensitivity analyses in AD

To assess the robustness of the hypothesized direction, reverse mediation models were conducted using regional GMV as the predictor, regional FDG metabolism as the mediator, and the corresponding cognitive measure as the outcome. The same covariates and analytical procedures were applied. These models were interpreted as sensitivity analyses and were not intended to establish causal direction.

For visuospatial function, rSTG FDG metabolism was positively associated with rIPL GMV (*β* = 0.283, *p* = 0.007). rIPL GMV significantly predicted better visuospatial performance after adjustment for rSTG FDG metabolism (*β* = 0.510, *p* < 0.001). The total effect of rIPL GMV on visuospatial function was significant, as was the direct effect. rSTG FDG metabolism exerted a significant partial mediating effect, accounting for 13.26% of the total effect and revealing a partial mediating mechanism underlying visuospatial function.

For executive function, lIPL FDG metabolism was positively associated with rAmyg GMV (*β* = 0.256, *p* = 0.025). Elevated rAmyg GMV significantly predicted better executive function after adjusting for lIPL FDG metabolism (*β* = 0.417, *p* < 0.001). The total effect of rAmyg GMV on executive function was significant (*β* = 0.499, *p* < 0.001), and the direct effect also achieved statistical significance (*β* = 0.449, *p* < 0.001). The bootstrap 95% CI for the indirect effect through lIPL FDG included zero [indirect effect: *β* = 0.048, 95% CI (−0.003, 0.129)], demonstrating that lIPL FDG metabolism did not exert a significant mediating effect between rAmyg GMV and executive function.

Regarding language ability, lSTG GMV positively predicted lSTG FDG metabolism (β = 0.540, *p* < 0.001). lSTG FDG metabolism showed no significant predictive effect on language ability after adjustment. The total and direct effects of lSTG GMV on language ability were both significant. The indirect effect of lSTG FDG metabolism was non-significant (indirect β = 0.058, 95% CI: −0.084 to 0.202), indicating no significant mediating mechanism of lSTG FDG metabolism underlying the association between lSTG GMV and language ability (Table 5).

**Table 5:** Reverse mediation sensitivity analyses in AD.

Cognitive function	Path relationship	X → M path (*β*, *p* value)	M → Y path (*β*, *p* value)	Total effect (*β*, *p* v alue)	Direct effect (*β*, *p* value)	Mediation effect (effect value, 95%CI)	Proportion of mediation effect	Model fit (*R*^2^, *p* value)
Visuospatial ability	Right inferior temporal volume (X) → right superior temporal gyrus FDG (M) → visuospatial score (Y)	0.283, *p* = 0.007	0.510, *p* < 0.001	0.578, *p* < 0.001	0.491, *p* < 0.001	0.087, (0.008, 0.199)	13.26%	0.373, *p* < 0.001
Executive function	Right amygdala GMV (X) → left inferior parietal lobule FDG metabolism (M) → executive score (Y)	0.256, *p* = 0.025	0.417, *p*<0.001	0.499, *p*<0.001	0.449, *p*<0.001	0.048, (−0.003, 0.129)	—	0.282, *p* < 0.001
Language ability	Left superior temporal gyrus GMV (X) → left superior temporal gyrus FDG metabolism (M) → language score (Y)	0.540, *p* < 0.001	0.083, *p* = 0.441	0.341, *p* = 0.004	0.289, *p* = 0.045	0.058, (−0.084, 0.202)	—	0.124, *p* = 0.045

#### 3.7.4 ApoE-adjusted mediation analysis and multiple-imputation sensitivity analysis

In AD patients, three separate FDG metabolism—GMV—cognitive function mediation models were tested using primary mediation analyses and multiple-imputation sensitivity analyses, with adjustment for sex, age, disease duration, years of education, eTIV, and ApoE ε4 carrier status.

For visuospatial ability, significant positive association between rSTG FDG and rIPL GMV and a significant total effect of rSTG FDG on visuospatial ability were observed in both complete-case and multiple imputation analyses. The M → Y path was non-significant in both models. The indirect mediating effect of rIPL GMV achieved statistical significance in the complete-case analysis, accounting for 15.59% of the total effect. Nevertheless, this mediation effect became non-significant after multiple imputation.

For executive function, both analyses showed significant total and direct effects, indicating that the association between lIPL FDG and executive function was primarily direct. In the complete-case analysis, neither the X → M path nor the indirect effect was significant. After multiple imputation, both the X → M and M → Y paths became significant; however, the indirect effect remained nonsignificant because its 95% CI included zero. Thus, neither analysis supported a significant mediating role of right amygdala GMV. After multiple imputation, the estimated mediation effect and proportion mediated decreased from 14.75 to 11.04%, but the overall conclusion remained unchanged.

For language ability, both analyses yielded significant X → M paths and total effects, demonstrating positive associations between lSTG FDG and lSTG GMV, as well as between lSTG FDG and language ability. The M → Y path, direct and indirect effects were non-significant in complete-case analysis. After multiple imputation, the M → Y path became significant, but the direct effect stayed non-significant and the indirect effect remained insignificant (95% CI [−0.013, 0.259], *p* = 0.076). Although the mediation proportion increased from 25.2 to 51.2%, no significant mediating effect of lSTG GMV was identified in either analysis (Supplementary Tables S20–S23).

## 4 Discussion

The 2018 NIA-AA biological framework conceptualizes AD as a dynamic Aβ–tau–neurodegeneration cascade, highlighting the central role of multimodal biomarkers for accurate dementia subtyping and early intervention (40). This study systematically integrated amyloid PET, sMRI, ^18^F-FDG PET, neuropsychological assessment, and ApoE genotyping to map cerebral structural and metabolic alterations in AD versus non-AD dementia, as well as their relationships with cognitive impairment, providing mechanistic insights and clinical implications for AD pathogenesis.

Compared with individuals with non-AD dementia, patients with Alzheimer’s disease exhibited more extensive neurodegeneration, characterized by significant gray matter atrophy in 34 regions and widespread cerebral glucose hypometabolism involving 43 regions. Notably, 23 regions demonstrated concurrent structural and metabolic abnormalities, predominantly involving the bilateral hippocampus, bilateral precuneus, multiple temporal subregions, various cingulate cortex subareas, parietal lobe regions, and prefrontal cortex subregions. Many of these overlapping regions are components of the default mode network (DMN), which is known to be particularly vulnerable to AD pathology (41). The most severely affected areas included the hippocampus, entorhinal cortex, posterior cingulate cortex, precuneus and parietotemporal cortices, consistent with canonical neuroimaging signatures of AD (42, 43). According to Braak pathological staging framework (43), tau pathology initially accumulates in medial temporal structures before spreading to association cortices during disease progression. As central hubs of the DMN, the precuneus and posterior cingulate cortex are especially susceptible to both gray matter loss and FDG hypometabolism (44), and dysfunction within these regions may further accelerate network disintegration and cognitive decline (41). The extensive overlap between structural and metabolic abnormalities observed in our study supports a close coupling between neuronal dysfunction and neurodegeneration and is consistent with previous multimodal imaging studies showing that combined MRI and FDG-PET provide complementary measures of disease severity.

In this cross-sectional cohort, global Aβ burden was not significantly correlated with regional atrophy or glucose hypometabolism. This observation should not be interpreted as evidence that these imaging abnormalities are independent of Aβ pathology. Several factors may have contributed to the absence of significant associations, including the nonlinear trajectory of Aβ accumulation, in which deposition may increase rapidly during the early stages and subsequently reach a relative plateau (40). In addition, global SUVR may not adequately capture regional or network-specific associations between Aβ burden and downstream abnormalities. Because the cohort primarily comprised patients with moderate-to-advanced AD and did not include CSF p-tau or other downstream biomarkers, the relationship between Aβ pathology, neurodegeneration, and metabolic alterations cannot be fully characterized. In the non-AD dementia group, higher global Aβ burden was cross-sectionally associated with higher MMSE and MoCA scores. These findings should not be interpreted causally or as evidence of a protective effect of Aβ. They may reflect disease heterogeneity, differences in disease stage, or residual confounding, and require confirmation in larger longitudinal studies with additional biomarkers.

In this exploratory analysis, no significant differences were observed among ApoE genotype groups after correction for multiple comparisons, consistent with previous reports (17). However, these null findings should be interpreted cautiously because of the small subgroup sizes, particularly the limited number of ε4 homozygotes, which may have reduced statistical power. Moreover, ApoE data may not have been missing completely at random, as most patients who declined genotyping had advanced AD. This may have led to underrepresentation of advanced-stage patients, potentially biasing genotype-related differences in gray matter volume, Aβ burden, and cerebral glucose metabolism and limiting the generalizability of the findings across disease stages. Larger longitudinal studies with pathologically homogeneous cohorts and multimodal biomarkers are warranted to clarify the stage-dependent effects of ApoE ε4 across the AD continuum.

Based on the correlation analyses of regional GMV and cerebral glucose metabolism across five cognitive domains, the current mediation screening confirmed that only visuospatial, executive, and language functions met the complete prerequisites for mediation modeling, whereas memory and attention domains were excluded due to insufficient structural and metabolic correlation robustness. This domain-specific difference reflects the heterogeneous neural damage patterns of AD cognitive impairment: memory dysfunction mainly manifests as functional neural activity decline rather than obvious structural atrophy (45), whereas no robust structure–metabolism relationships were identified for attention in our cohort. In contrast, impairments in visuospatial, executive, and language functions appear to arise from the combined effects of cerebral hypometabolism and gray matter atrophy (24), providing a basis for subsequent mediation analyses.

Further analyses revealed distinct metabolism–structure mediation effects across cognitive domains. For visuospatial function, right inferior parietal lobule volume mediated 40.36% of the association between right superior temporal gyrus metabolism and cognition. These temporoparietal regions are key components of the dorsal visual network involved in spatial perception and integration (46, 47). Consistent with evidence that metabolic and structural abnormalities may occur at different stages of AD (48), our findings suggest that metabolic dysfunction and gray matter loss within temporoparietal regions may jointly contribute to disruptions in neural systems supporting visuospatial processing. This interpretation is further supported by the fact that these temporoparietal regions overlap with key hubs of the DMN, one of the earliest and most vulnerable networks affected by Aβ and tau pathology in AD (49). Dysfunction within this network may contribute to the disorientation and impaired spatial processing frequently observed in affected individuals (41, 50).

For executive function, right amygdala GMV statistically accounted for 35.6% of the association between left inferior parietal metabolism and executive performance. The inferior parietal cortex is involved in attentional allocation and higher-order cognitive control, whereas the amygdala contributes to emotional processing, behavioral regulation, and motivational responses (51–53). Previous studies have linked amygdala atrophy with executive dysfunction and neuropsychiatric manifestations in AD, including apathy, agitation, and impaired behavioral control (54). In the present study, the observed indirect effect suggests that the association between left inferior parietal glucose metabolism and executive performance was partly represented by individual differences in right amygdala GMV. However, this result does not demonstrate that parietal hypometabolism causes secondary limbic degeneration. The association may instead reflect coordinated abnormalities across cortical and limbic regions, reciprocal metabolism–structure relationships, or shared pathological influences affecting both regions (55–57).

For language function, left superior temporal gyrus volume mediated approximately 50% of the association between left superior temporal gyrus glucose metabolism and language performance. The superior temporal gyrus is a key component of the language network involved in speech comprehension and semantic processing (58). Given the significant association between FDG metabolism and gray matter volume, our findings suggest that metabolic dysfunction and structural alterations within this region may jointly contribute to language decline. The mediation effect further suggests that structural integrity of this region may represent an important link between regional metabolic abnormalities and language impairment in AD. Given the central role of the superior temporal cortex in language processing, concurrent metabolic and structural abnormalities within this region may contribute to impaired naming, comprehension, and verbal expression (59, 60).

Reverse mediation analyses provided only limited support for a consistent alternative pathway. Although rSTG FDG metabolism partially mediated the association between rIPL GMV and visuospatial function, this finding should not be interpreted as evidence of reverse causality. No significant indirect effects were observed for executive or language function, suggesting that the relationships among regional metabolism, brain structure, and cognition may be domain-specific rather than uniform across cognitive domains. Given the cross-sectional design, neither temporal ordering nor causal direction can be established. After adjustment for ApoE ε4 status and multiple imputation for missing genotype data, the total and direct effects were broadly consistent with the primary analyses, whereas the indirect effects were not robust. In particular, the visuospatial mediation effect lost statistical significance after imputation, and no significant mediation was observed for executive or language function. These findings suggest that the overall associations among FDG metabolism, GMV, and cognition may be more reliable than the proposed mediation pathways. The weaker robustness of the indirect effects may reflect the greater sensitivity of mediation models to missing data, limited sample size, measurement error, and model specification. Because indirect effects are estimated as the product of multiple paths, they are typically smaller and statistically less stable than total or direct effects, and may therefore be more vulnerable to attenuation after imputation or covariate adjustment. This issue may be particularly relevant in the present study given the substantial proportion of missing ApoE genotyping data, which may have further reduced the precision of the mediation estimates. Overall, these findings do not establish that atrophy leads to reduced metabolism, and the observed associations should not be interpreted causally. All mediation models examined here were exploratory and were not designed to determine causal directionality; confirmation of any specific causal pathway will require longitudinal data.

Several interconnected mechanisms may underlie the associations among reduced FDG metabolism, lower GMV, and cognitive impairment in AD (40, 61). Synaptic dysfunction may represent a shared pathway, as impaired synaptic activity reduces glucose utilization, while synaptic loss and dendritic remodeling contribute to neuronal loss and cognitive decline (62–64). Aβ and tau pathology, neuroinflammation, cerebrovascular dysfunction, and disrupted network connectivity may further impair neuronal function, glucose delivery, synaptic integrity, and tissue maintenance (65–71). Collectively, our domain-specific mediation models suggest that metabolic, structural, and cognitive abnormalities in AD are interrelated but do not necessarily follow a unidirectional sequence. Instead, cognitive impairment may reflect the combined effects of metabolic and structural alterations across specialized brain networks. Integrating these measures may improve individualized assessment of disease burden, monitoring of cognitive decline, and identification of targets for network-based interventions.

## 5 Limitations

Several limitations should be acknowledged. First, the cross-sectional design precludes determination of the temporal sequence underlying the associations examined in the mediation models. In addition, the analyses did not account for potentially important confounders, such as concomitant medications, baseline cognitive reserve, or white matter hyperintensity burden. These factors may influence brain metabolism, GMV, and cognitive performance, potentially attenuating or exaggerating the observed associations and mediation effects. Longitudinal studies with repeated assessments and more comprehensive confounder adjustment are needed to clarify these temporal relationships. Second, our cohort primarily comprised patients with moderate-to-advanced AD, limiting the generalizability of the findings to individuals with preclinical or mild AD and to the full AD continuum. Third, the etiologies of Aβ-negative dementia were not systematically subtyped. These cases may include heterogeneous pathologies, such as α-synucleinopathies, cerebrovascular disease, frontotemporal lobar degeneration or mixed pathologies. Such etiological heterogeneity and concomitant lesions may obscure subtle imaging differences associated with ApoE genotype and potentially attenuate or confound its observed effects.

## 6 Conclusion

In this cross-sectional study, patients with AD showed more extensive reductions in regional gray matter volume and cerebral glucose metabolism than patients with non-AD dementia, with substantial overlap across hippocampal, temporoparietal, cingulate, and precuneus regions. Associations between regional brain abnormalities and cognitive performance were domain-specific: visuospatial function was most closely related to the right inferior parietal lobule and right superior temporal gyrus, whereas executive and language functions were associated with limbic, parietal, and temporal regions.

Primary mediation analyses demonstrated that regional GMV significantly mediated the effects of FDG metabolism on visuospatial, executive, and language function, accounting for 40.36, 35.60, and 49.99% of the total effects, respectively. Reverse mediation models identified only a weak indirect effect of rSTG FDG on the association between rIPL GMV and visuospatial ability, accounting for 13.26% of the total effect. However, these effects were not consistently supported by ApoE ε4-adjusted multiple-imputation sensitivity analyses.

Therefore, our results reveal interconnections among regional brain metabolism, grey matter morphology and cognitive function, yet they cannot verify a unidirectional causal pathway where grey matter atrophy triggers metabolic reduction or cognitive decline. Taken together, combining metabolic, structural and cognitive measurements helps delineate the multidimensional disease burden of AD and pinpoint domain-specific neural substrates. Longitudinal studies with comprehensive confounding adjustment and aetiological subgrouping are required to further elucidate the temporal dynamics and causal relationships underlying these associations.

## Data Availability

The original contributions presented in the study are included in the article/Supplementary material, further inquiries can be directed to the corresponding authors.
