# Supplementary material for: Gray matter atrophy mediates the association between cerebral hypometabolism and cognitive impairment in Alzheimer’s disease
Source: Front Neurol. 2026 Sep 15;17:1919778. doi: 10.3389/fneur.2026.1919778 (PMC13621415; doi:10.3389/fneur.2026.1919778)
Supplement: Supplementary file 1 [file Table_1.docx]

Supplementary Material

# Supplementary Tables

## **List of Supplementary Tables**

Table S1. Brain Regions with Differences in Gray Matter Volume between the AD Group and the Non-AD Dementia Group ............................................................................................................................................................................ 3

Table S2. Brain Regions with Differences in ^18^F-FDG-PET SUVR Values between the AD Group and the Non-AD Dementia Group ........................................................................................................................................................... 9

Table S3. **Correlation between Whole-Brain Aβ Deposition and Neuropsychological Scale Scores in AD /non-AD Patients ......................................**................................................................................................................................. 18

Table S4. Correlation between Whole-Brain Aβ Deposition and Regional Gray Matter Volume in AD group ........................................................................................................................................................................... 19

Table S5. Correlation between Whole-Brain Aβ Deposition and Regional FDG Metabolism in AD group............................................................................................................................................................................ 26

Table S6. Correlation between Whole-Brain Aβ Deposition and Regional Gray Matter Volume in non-AD dementia group ........................................................................................................................................................................... 32

Table S7. Correlation between Whole-Brain Aβ Deposition and Regional FDG Metabolism in non-AD dementia group ........................................................................................................................................................................... 39

Table S8. Comparison of Clinical Data and Neuropsychological Scale Scores among ApoE ε4 Homozygous, Heterozygous, and Non-Carrier in AD group...............................................................................................................47

Table S9. **Comparison of Gray Matter Volume among ApoE ε4 Homozygous, Heterozygous, and Non-Carrier in AD group** ........................................................................................................................................................................... 47

Table S10. **Comparison of AV45-PET SUVR value among ApoE ε4 Homozygous, Heterozygous, and Non-Carrier in AD group .................................................................................................................................................................**53

Table S11. Comparison of ^18^F-FDG PET SUVR Values among ApoE ε4 Homozygous, Heterozygous, and Non-Carrier in AD Groups .................................................................................................................................................. 57

Table S12. Comparison of clinical features and cognitive scale scores between genotyped and non-genotyped subgroups ................................................................................................................................................................... 62

Table S13. Comparison of regional **Gray Matter Volume** between genotyped and non-genotyped subgroup .............................................................................................................................................................62 Table S14 Comparison of regional FDG Metabolism between genotyped and non-genotyped subgroups..................................................................................................................................................................... 69

Table S15. Comparison of regional AV45 SUVR between genotyped and non-genotyped subgroups .....................................................................................................................................................................75

Table S16. Correlation between Cognitive Domains and Regional Gray Matter Volume in AD group .......................................................................................................................................................................... 81

### Table S17. Correlation between Cognitive Domains and Regional FDG Metabolism in AD group .......................................................................................................................................................................... 93

Table S18.Correlation between Cognitive Domains and Regional Gray Matter Volume in non-AD group............................................................................................................................................................................96

Table S19. Correlation between Cognitive Domains and Regional FDG Metabolism in non-AD group..........................................................................................................................................................................115

**Table S20. Screening Results of Target Brain Regions for Cognitive Domains.......................................................134**

Table S21. ApoE-Adjusted Mediation Analysis and Multiple Imputation Sensitivity Analysis for Visuospatial Ability........................................................................................................................................................................137

Table S22. ApoE-Adjusted Mediation Analysis and Multiple Imputation Sensitivity Analysis for Executive function .....................................................................................................................................................................137

Table S23. ApoE-Adjusted Mediation Analysis and Multiple Imputation Sensitivity Analysis for Language ability..........................................................................................................................................................................138

Table S24. Sex-related differences in regional Aβ deposition in the non-AD group................................................139

**Table S1. Brain Regions with Differences in Gray Matter Volume between the AD Group and the Non-AD Dementia Group**

| **Brain region** | **AD group**  **(n=92)** | **Non-AD dementia group**  **(n=42)** | **F value** | **P value** | **ηₚ²** | **FDR-P** |
| --- | --- | --- | --- | --- | --- | --- |
| lh-precuneus | 7763.50 (6905.00, 8310.75) | 8874.50 (8329.25, 8874.50) | 35.398 | 2.41E-08 | 0.217 | 2.07E-06* |
| Right-Hippocampus | 3144.16 ± 482.13 | 3703.22 ± 641.56 | 28.498 | 4.13E-07 | 0.182 | 1.78E-05* |
| rh-inferiorparietal | 11045.87 ± 1905.20 | 12850.55 ± 2000.25 | 26.572 | 9.38E-07 | 0.172 | 2.69E-05* |
| Left-Hippocampus | 3012.25 (2777.50, 3238.38) | 3551.80 (2835.98, 4052.15) | 18.064 | 4.08E-05 | 0.124 | 0.001* |
| rh-middletemporal | 9232.10 ± 1649.16 | 10360.83 ± 1665.00 | 16.847 | 7.17E-05 | 0.116 | 0.001* |
| lh-superiorparietal | 11857.86 ± 1661.64 | 18279.40 ± 2227.94 | 15.736 | 0.000 | 0.109 | 0.001* |
| rh-lateraloccipital | 10002.79 ± 1542.85 | 11204.50 ± 1703.33 | 15.509 | 0.000 | 0.108 | 0.001* |
| rh-superiorparietal | 10185.04 ± 1498.96 | 11284.60 ± 1627.79 | 15.809 | 0.000 | 0.110 | 0.001* |
| rh-inferiortemporal | 8360.45 ± 1598.35 | 9467.33 ± 1763.85 | 14.714 | 0.000 | 0.103 | 0.002* |
| rh-precuneus | 8240.20 ± 1237.00 | 9080.71 ± 1272.55 | 13.886 | 0.000 | 0.098 | 0.002* |
| rh-superiortemporal | 9539.17 ± 1289.37 | 10503.93 ± 1495.77 | 13.289 | 0.000 | 0.094 | 0.003* |
| rh-rostralmiddlefrontal | 12272.50 (10948.25, 13613.25) | 13489.50 (12556.75, 14475.25) | 13.004 | 0.000 | 0.092 | 0.003* |
| rh-parstriangularis | 3469.17 ± 584.30 | 3871.51 ± 608.40 | 12.789 | 0.000 | 0.091 | 0.003* |
| rh-bankssts | 1547.30 ± 298.98 | 1747.31 ± 351.88 | 12.551 | 0.001 | 0.089 | 0.003* |
| rh-fusiform | 7552.50 (6871.50, 8771.25) | 8510.50 (7843.75, 9527.50) | 12.156 | 0.001 | 0.087 | 0.004* |
| Right-Amygdala | 1398.72 ± 310.53 | 1619.04 ± 381.37 | 11.346 | 0.001 | 0.081 | 0.005* |
| lh-inferiortemporal | 8735.00 (7438.75, 9521.00) | 9583.50 (8264.00, 11186.00) | 9.924 | 0.002 | 0.072 | 0.010* |
| lh-rostralmiddlefrontal | 11762.30 ± 1765.97 | 12768.88 ± 1621.67 | 9.621 | 0.002 | 0.070 | 0.011* |
| lh-bankssts | 1547.30 ± 298.98 | 1747.31 ± 351.88 | 9.357 | 0.003 | 0.068 | 0.012* |
| lh-inferiorparietal | 9615.00 ± 1756.53 | 10662.53 ± 1687.78 | 9.283 | 0.003 | 0.068 | 0.012* |
| lh-superiorfrontal | 18279.40 ± 2227.94 | 19489.19 ± 2653.24 | 8.688 | 0.004 | 0.064 | 0.014* |
| rh-parahippocampal | 1560.90 ± 260.22 | 1719.24 ± 335.43 | 8.717 | 0.004 | 0.064 | 0.014* |
| Right-Thalamus | 6292.74 ± 830.84 | 6782.86 ± 881.62 | 8.842 | 0.004 | 0.065 | 0.014* |
| rh-transversetemporal | 758.53 ± 147.57 | 844.24 ± 168.10 | 7.583 | 0.007 | 0.056 | 0.022* |
| lh-supramarginal | 8763.58 ± 1730.05 | 9511.83 ± 1363.41 | 7.331 | 0.008 | 0.054 | 0.025* |
| rh-isthmuscingulate | 2105.63 ± 352.81 | 2286.83 ± 417.89 | 6.435 | 0.012 | 0.048 | 0.038* |
| lh-isthmuscingulate | 2256.50 (1991.00, 2496.00) | 2467.00 (2208.00, 2787.25) | 6.021 | 0.015 | 0.045 | 0.038* |
| lh-middletemporal | 8537.28 ± 1511.17 | 9269.64 ± 2016.78 | 6.032 | 0.015 | 0.045 | 0.038* |
| rh-posteriorcingulate | 2543.78 ± 531.10 | 2796.26 ± 508.96 | 6.108 | 0.015 | 0.046 | 0.038* |
| rh-rostralanteriorcingulate | 1565.28 ± 437.92 | 1761.95 ± 373.95 | 6.183 | 0.014 | 0.046 | 0.038* |
| rh-frontalpole | 1013.83 ± 186.54 | 1106.88 ± 193.07 | 6.020 | 0.015 | 0.045 | 0.038* |
| Left-Putamen | 4090.25 (3618.25, 4554.15) | 4572.70 (3956.80, 4842.50) | 6.352 | 0.013 | 0.047 | 0.038* |
| Right-Putamen | 4320.25±756.59 | 4675.07±796.18 | 6.280 | 0.013 | 0.047 | 0.038* |
| rh-medialorbitofrontal | 4695.65 ± 565.62 | 4961.60 ± 647.80 | 5.549 | 0.020 | 0.042 | 0.048* |
| Right-Caudate | 3081.21 ± 463.89 | 3331.75 ± 661.22 | 5.118 | 0.025 | 0.038 | 0.059 |
| Left-Amygdala | 1303.18 ± 293.05 | 1457.36 ± 457.93 | 4.976 | 0.027 | 0.037 | 0.062 |
| lh-parahippocampal | 1658.24 ± 335.40 | 1844.10 ± 421.84 | 4.920 | 0.028 | 0.037 | 0.062 |
| rh-supramarginal | 7875.08 ± 1350.92 | 8401.33 ± 1185.56 | 4.756 | 0.031 | 0.036 | 0.065 |
| rh-superiorfrontal | 17776.50 (16439.75, 19382.50) | 18386.50 (17087.00, 19799.00) | 4.651 | 0.033 | 0.035 | 0.067 |
| rh-parsopercularis | 3182.89 ± 558.77 | 3400.29 ± 566.99 | 4.509 | 0.036 | 0.034 | 0.071 |
| rh-paracentral | 3557.52 ± 460.67 | 3749.26 ± 596.12 | 4.447 | 0.037 | 0.034 | 0.072 |
| rh-insula | 5967.64 ± 782.01 | 6265.19 ± 855.87 | 4.214 | 0.042 | 0.032 | 0.081 |
| Left-Thalamus | 5898.75 (5365.68, 6517.33) | 6437.20 (5843.35, 6638.35) | 4.111 | 0.045 | 0.031 | 0.084 |
| rh-lateralorbitofrontal | 10002.79 ± 1542.85 | 11204.50 ± 1703.33 | 3.766 | 0.054 | 0.029 | 0.100 |
| lh-caudalmiddlefrontal | 4500.50 (3980.00, 5338.75) | 4966.00 (4565.00, 5611.50) | 3.474 | 0.065 | 0.026 | 0.116 |
| lh-fusiform | 7925.33 ± 1311.00 | 8523.10 ± 1748.23 | 3.345 | 0.070 | 0.025 | 0.121 |
| rh-lingual | 6199.08 ± 919.34 | 6525.81 ± 940.27 | 3.331 | 0.070 | 0.025 | 0.121 |
| rh-caudalanteriorcingulate | 1672.65 ± 449.93 | 1804.98 ± 371.08 | 2.433 | 0.121 | 0.019 | 0.197 |
| rh-cuneus | 3020.76 ± 448.28 | 3164.12 ± 537.87 | 2.442 | 0.121 | 0.019 | 0.197 |
| lh-entorhinal | 1385.65 ± 380.91 | 1547.40 ± 530.20 | 2.072 | 0.152 | 0.016 | 0.226 |
| lh-lateraloccipital | 10256.49 ± 1720.70 | 10799.00 ± 1565.84 | 2.071 | 0.153 | 0.016 | 0.226 |
| rh-caudalmiddlefrontal | 4649.86 ± 971.30 | 4923.02 ± 981.81 | 2.104 | 0.149 | 0.016 | 0.226 |
| Right-Pallidum | 1816.26 ± 260.00 | 1762.10 ± 225.67 | 2.140 | 0.146 | 0.016 | 0.226 |
| lh-temporalpole | 2336.50 (2014.50, 2564.00) | 2351.50 (1841.50, 2552.25) | 2.033 | 0.156 | 0.016 | 0.228 |
| lh-lateralorbitofrontal | 6356.12 ± 711.90 | 6527.79 ± 977.90 | 1.751 | 0.188 | 0.013 | 0.265 |
| lh-transversetemporal | 995.00 (882.50, 1097.50) | 1078.00 (920.25, 1191.00) | 1.768 | 0.186 | 0.014 | 0.265 |
| lh-parsorbitalis | 1877.62 ± 264.58 | 1938.71 ± 234.10 | 1.631 | 0.204 | 0.013 | 0.283 |
| lh-postcentral | 8421.23 ± 1059.48 | 8718.12 ± 1132.77 | 1.443 | 0.232 | 0.011 | 0.312 |
| lh-rostralanteriorcingulate | 2137.03 ± 460.57 | 2248.26 ± 544.26 | 1.447 | 0.231 | 0.011 | 0.312 |
| lh-superiortemporal | 10091.53 ± 1576.15 | 10543.05 ± 1730.93 | 1.415 | 0.236 | 0.011 | 0.313 |
| lh-precentral | 11832.50 (11089.75, 12696.75) | 12242.50 (11282.00, 13358.25) | 1.326 | 0.252 | 0.010 | 0.328 |
| Left-Caudate | 2986.50 (2734.08, 3266.45) | 3247.75 (2710.10, 3538.43) | 1.228 | 0.270 | 0.010 | 0.346 |
| rh-precentral | 11605.89 ± 1625.11 | 11911.88 ± 1869.59 | 1.027 | 0.313 | 0.008 | 0.396 |
| lh-medialorbitofrontal | 4432.50 (4086.50, 4849.50) | 4703.00 (4159.00, 5072.25) | 0.887 | 0.348 | 0.007 | 0.422 |
| lh-parsopercularis | 3728.25 ± 689.61 | 3875.64 ± 684.25 | 0.904 | 0.343 | 0.007 | 0.422 |
| rh-entorhinal | 1433.75 ± 415.24 | 1526.21 ± 433.20 | 0.885 | 0.349 | 0.007 | 0.422 |
| rh-postcentral | 8184.18 ± 1339.56 | 8418.57 ± 1339.56 | 0.802 | 0.372 | 0.006 | 0.445 |
| lh-posteriorcingulate | 2543.78 ± 531.10 | 2796.26 ± 508.96 | 0.724 | 0.396 | 0.006 | 0.467 |
| rh-parsorbitalis | 2321.10 ± 313.48 | 2362.52 ± 364.75 | 0.645 | 0.423 | 0.005 | 0.492 |
| rh-temporalpole | 2381.00 ± 437.41 | 2433.21 ± 617.33 | 0.625 | 0.431 | 0.005 | 0.494 |
| lh-pericalcarine | 1905.95 ± 435.16 | 1845.12 ± 377.02 | 0.518 | 0.473 | 0.004 | 0.535 |
| lh-lingual | 5598.50 (5040.50, 6215.25) | 5484.50 (5210.50, 6592.50) | 0.451 | 0.503 | 0.004 | 0.562 |
| Left-Pallidum | 1858.68 ± 234.84 | 1886.28 ± 273.17 | 0.231 | 0.632 | 0.002 | 0.688 |
| rh-pericalcarine | 2262.60 ± 428.47 | 3469.17 ± 584.30 | 0.210 | 0.648 | 0.002 | 0.696 |
| lh-cuneus | 2688.05 ± 407.97 | 2730.83 ± 505.95 | 0.183 | 0.669 | 0.001 | 0.711 |
| lh-parstriangularis | 2960.83 ± 540.15 | 3875.64 ± 684.25 | 0.118 | 0.731 | 0.001 | 0.767 |
| lh-insula | 6159.28 ± 696.61 | 6186.43 ± 968.50 | 0.095 | 0.759 | 0.001 | 0.786 |
| lh-frontalpole | 891.50 (785.25, 1020.50) | 911.50 (816.25, 993.75) | 0.037 | 0.847 | 0.000 | 0.867 |
| lh-paracentral | 3182.53 ± 399.20 | 3223.40 ± 540.02 | 0.030 | 0.862 | 0.000 | 0.872 |
| lh-caudalanteriorcingulate | 1331.00 (1135.25, 1740.00) | 1434.50 (1208.25, 1715.00) | 0.005 | 0.945 | 0.000 | 0.945 |

*FDR-P≤0.05, statistically significant difference.

**Table S2. Brain Regions with Differences in ^18^F-FDG-PET SUVR Values between the AD Group and the Non-AD Dementia Group**

| **Brain Region** | **AD group**  **(n=92)** | **Non-AD dementia group**  **(n=42)** | **F value** | **P value** | **Partial η²** | **FDR-adjusted P** |
| --- | --- | --- | --- | --- | --- | --- |
| Right-Hippocampus | 1.04（0.96，1.15） | 1.25（1.13，1.34） | 27.853 | <0.001 | 0.179 | <0.001* |
| rh-entorhinal | 0.94±0.18 | 1.12±0.20 | 25.574 | <0.001 | 0.167 | <0.001* |
| rh-isthmuscingulate | 1.53（1.41，1.71） | 1.83（1.61，1.83） | 22.550 | <0.001 | 0.150 | <0.001* |
| lh-posteriorcingulate | 1.65（1.46，1.88） | 2.03（1.75，2.28） | 21.573 | <0.001 | 0.144 | <0.001* |
| rh-precuneus | 1.72±0.38 | 2.02±0.37 | 19.269 | <0.001 | 0.131 | <0.001* |
| lh-isthmuscingulate | 1.44（1.32，1.63） | 1.81（1.52，2.01） | 18.469 | <0.001 | 0.126 | <0.001* |
| rh-bankssts | 1.63±0.36 | 1.92±0.31 | 18.289 | <0.001 | 0.125 | <0.001* |
| rh-posteriorcingulate | 1.72±0.31 | 1.94±0.39 | 18.133 | <0.001 | 0.124 | <0.001* |
| rh-inferiortemporal | 1.44±0.29 | 1.70±0.27 | 17.415 | <0.001 | 0.120 | <0.001* |
| rh-middletemporal | 1.51±0.35 | 1.77±0.30 | 16.747 | <0.001 | 0.116 | <0.001* |
| lh-bankssts | 1.62±0.38 | 1.91±0.32 | 16.501 | <0.001 | 0.114 | <0.001* |
| lh-precuneus | 1.67（1.50，1.92） | 2.04（1.75，2.33） | 15.557 | <0.001 | 0.108 | 0.001* |
| Left-Hippocampus | 1.01（0.91，1.12） | 1.18（1.03，1.18） | 14.627 | <0.001 | 0.103 | 0.001* |
| lh-inferiorparietal | 1.59±0.41 | 1.90±0.42 | 14.687 | <0.001 | 0.103 | 0.001* |
| lh-inferiortemporal | 1.41±0.31 | 1.67±0.36 | 14.800 | <0.001 | 0.104 | 0.001* |
| rh-inferiorparietal | 1.57±0.38 | 1.87±0.42 | 14.869 | <0.001 | 0.104 | 0.001* |
| rh-superiortemporal | 1.50±0.26 | 1.69±0.32 | 13.266 | <0.001 | 0.094 | 0.002* |
| lh-supramarginal | 1.58（1.34，1.81） | 1.87（1.58，2.06） | 13.078 | <0.001 | 0.093 | 0.002* |
| rh-rostralmiddlefrontal | 1.81±0.89 | 1.92±0.25 | 12.801 | <0.001 | 0.091 | 0.002* |
| rh-caudalmiddlefrontal | 1.81±0.39 | 2.09±0.43 | 12.384 | <0.001 | 0.088 | 0.003* |
| rh-supramarginal | 1.67±0.31 | 1.83±0.35 | 12.126 | <0.001 | 0.087 | 0.003* |
| lh-caudalmiddlefrontal | 1.83±0.37 | 2.08±0.41 | 11.432 | <0.001 | 0.082 | 0.004* |
| lh-superiorparietal | 1.67±0.31 | 1.93±0.37 | 10.705 | 0.001 | 0.077 | 0.005* |
| lh-middletemporal | 1.49±0.34 | 1.69±0.32 | 10.439 | 0.002 | 0.075 | 0.006* |
| rh-insula | 1.30（1.19，1.47） | 1.46（1.33，1.77） | 10.315 | 0.002 | 0.075 | 0.006* |
| rh-medialorbitofrontal | 1.55±0.25 | 1.71±0.27 | 9.706 | 0.002 | 0.070 | 0.008* |
| lh-caudalanteriorcingulate | 1.40±0.19 | 1.53±0.23 | 9.415 | 0.003 | 0.069 | 0.008* |
| lh-fusiform | 1.50±0.29 | 1.69±0.34 | 9.324 | 0.003 | 0.068 | 0.008* |
| rh-lateralorbitofrontal | 1.64±0.26 | 1.80±0.28 | 9.438 | 0.003 | 0.069 | 0.008* |
| rh-superiorparietal | 1.66±0.35 | 1.89±0.38 | 9.352 | 0.003 | 0.068 | 0.008* |
| lh-superiortemporal | 1.49±0.69 | 1.66±0.31 | 9.208 | 0.003 | 0.067 | 0.008* |
| lh-rostralmiddlefrontal | 1.86±0.40 | 2.08±0.78 | 8.800 | 0.004 | 0.064 | 0.010* |
| rh-fusiform | 1.53±0.27 | 1.70±0.32 | 8.404 | 0.004 | 0.062 | 0.012* |
| lh-insula | 1.31（1.21，1.45） | 1.42（1.28，1.73） | 7.205 | 0.008 | 0.053 | 0.021* |
| lh-medialorbitofrontal | 1.53±0.24 | 1.67±0.33 | 6.906 | 0.010 | 0.051 | 0.024* |
| rh-rostralanteriorcingulate | 1.40±0.25 | 1.54±0.33 | 6.662 | 0.011 | 0.049 | 0.027* |
| rh-parsopercularis | 1.76±0.32 | 1.92±0.36 | 6.511 | 0.012 | 0.048 | 0.028* |
| lh-postcentral | 1.78±0.32 | 1.93±0.27 | 6.271 | 0.014 | 0.047 | 0.031* |
| rh-frontalpole | 1.79±0.38 | 1.96±0.41 | 6.232 | 0.014 | 0.046 | 0.031* |
| lh-lateralorbitofrontal | 1.68±0.26 | 1.80±0.33 | 5.806 | 0.017 | 0.043 | 0.038* |
| lh-rostralanteriorcingulate | 1.38（1.24，1.57） | 1.49（1.28，1.80） | 5.693 | 0.018 | 0.043 | 0.039* |
| rh-parstriangularis | 1.83±0.36 | 1.99±0.35 | 5.722 | 0.018 | 0.043 | 0.039* |
| Left-Thalamus | 1.47（1.37，1.57） | 1.59（1.42，1.72） | 5.272 | 0.023 | 0.040 | 0.047* |
| lh-parstriangularis | 1.83±0.35 | 1.99±0.38 | 4.917 | 0.028 | 0.037 | 0.055 |
| Right-Thalamus | 1.48（1.40，1.58） | 1.59（1.43，1.71） | 4.830 | 0.030 | 0.036 | 0.056 |
| rh-postcentral | 1.77±0.31 | 1.91±0.31 | 4.851 | 0.029 | 0.037 | 0.056 |
| rh-parahippocampal | 1.16±0.23 | 1.26±0.33 | 4.630 | 0.033 | 0.035 | 0.060 |
| rh-temporalpole | 1.12（0.97，1.25） | 1.26（1.03，1.38） | 4.649 | 0.033 | 0.035 | 0.060 |
| rh-superiorfrontal | 1.71±0.32 | 1.84±0.34 | 4.536 | 0.035 | 0.034 | 0.062 |
| rh-caudalanteriorcingulate | 1.46±0.22 | 1.56±0.23 | 4.268 | 0.041 | 0.032 | 0.071 |
| lh-parsopercularis | 1.76（1.55，1.94） | 1.89（1.63，2.15） | 4.021 | 0.047 | 0.030 | 0.077 |
| Right-Amygdala | 1.05（0.94，1.14） | 1.11（0.97，1.18） | 3.676 | 0.057 | 0.028 | 0.093 |
| lh-parahippocampal | 1.15（1.01，1.26） | 1.26（1.09，1.38） | 3.525 | 0.063 | 0.027 | 0.098 |
| lh-temporalpole | 1.11（1.01，1.25） | 1.26（1.00，1.36） | 3.308 | 0.071 | 0.025 | 0.109 |
| lh-paracentral | 1.70±0.28 | 1.80±0.18 | 3.115 | 0.080 | 0.024 | 0.121 |
| Right-Caudate | 1.52（1.32，1.69） | 1.68（1.38，1.85） | 2.962 | 0.088 | 0.023 | 0.130 |
| rh-paracentral | 1.73±0.27 | 1.83±0.21 | 2.899 | 0.091 | 0.022 | 0.133 |
| lh-entorhinal | 0.91±0.17 | 0.97±0.27 | 2.699 | 0.103 | 0.021 | 0.145 |
| lh-frontalpole | 1.83±0.35 | 1.92±0.41 | 2.660 | 0.105 | 0.020 | 0.145 |
| rh-precentral | 1.82±0.30 | 1.92±0.25 | 2.569 | 0.111 | 0.020 | 0.151 |
| lh-lingual | 1.79±0.33 | 1.90±0.27 | 2.468 | 0.119 | 0.019 | 0.158 |
| rh-cuneus | 1.92±0.36 | 2.03±0.33 | 2.272 | 0.134 | 0.017 | 0.176 |
| lh-precentral | 1.81±0.30 | 1.89±0.21 | 2.198 | 0.141 | 0.017 | 0.181 |
| lh-superiorfrontal | 1.70±0.31 | 2.04±0.39 | 2.162 | 0.144 | 0.017 | 0.181 |
| rh-parsorbitalis | 1.70±0.39 | 1.81±0.44 | 2.173 | 0.143 | 0.017 | 0.181 |
| lh-pericalcarine | 2.18±0.46 | 2.32±0.35 | 1.816 | 0.180 | 0.014 | 0.221 |
| rh-pericalcarine | 2.13±0.45 | 1.77±0.31 | 1.825 | 0.179 | 0.014 | 0.221 |
| Left-Caudate | 1.49（1.24，1.66） | 1.60（1.38，1.76） | 1.684 | 0.197 | 0.013 | 0.238 |
| lh-cuneus | 1.94±0.38 | 2.04±0.34 | 1.596 | 0.209 | 0.012 | 0.249 |
| Right-Pallidum | 1.34（1.23，1.44） | 1.37（1.28，1.53） | 1.408 | 0.238 | 0.011 | 0.277 |
| rh-transversetemporal | 1.88±0.47 | 1.99±0.55 | 1.410 | 0.237 | 0.011 | 0.277 |
| lh-lateraloccipital | 1.78±0.40 | 1.87±0.40 | 1.240 | 0.267 | 0.010 | 0.300 |
| Right-Putamen | 1.97（1.84，2.19） | 2.12（1.94，2.40） | 1.092 | 0.298 | 0.008 | 0.327 |
| lh-transversetemporal | 1.98±0.49 | 2.07±0.59 | 1.088 | 0.299 | 0.008 | 0.327 |
| rh-lingual | 1.79±0.31 | 1.87±0.29 | 0.906 | 0.343 | 0.007 | 0.371 |
| rh-lateraloccipital | 1.79±0.36 | 1.85±0.40 | 0.773 | 0.381 | 0.006 | 0.407 |
| lh-parsorbitalis | 1.74±0.43 | 1.77±0.46 | 0.211 | 0.647 | 0.002 | 0.684 |
| Left-Pallidum | 1.24（1.16，1.49） | 1.27（1.16，1.33） | 0.079 | 0.779 | 0.001 | 0.797 |
| Left-Putamen | 1.98（1.81，2.13） | 2.01（1.91，2.24） | 0.025 | 0.875 | <0.001 | 0.885 |
| Left-Amygdala | 0.98（0.91，1.05） | 1.02（0.93，1.08） | 0.003 | 0.957 | <0.001 | 0.957 |

*FDR-P≤0.05, statistically significant difference.

**Table S3. Correlation between Whole-Brain Aβ Deposition and Neuropsychological Scale Scores in AD /non-AD Patients**

| Group | **Neuropsychological Scale** | B | SE | Beta | t | p | CI low | CI high | R^2^ | Adj R^2^ | FDR-P |
| --- | --- | --- | --- | --- | --- | --- | --- | --- | --- | --- | --- |
| AD | MMSE | 3.5376 | 3.9916 | 0.0941 | 0.8863 | 0.3779 | -4.3975 | 11.4726 | 0.0573 | 0.0025 | 0.6557 |
|  | MOCA | -1.3922 | 3.5584 | -0.041 | -0.3912 | 0.6966 | -8.466 | 5.6817 | 0.0831 | 0.0298 | 0.8707 |
|  | ADL | -9.3212 | 6.5926 | -0.148 | -1.4139 | 0.161 | -22.4268 | 3.7843 | 0.0844 | 0.0312 | 0.6557 |
|  | HAMA | 0.3473 | 3.8851 | 0.0094 | 0.0894 | 0.929 | -7.376 | 8.0707 | 0.077 | 0.0234 | 0.929 |
|  | HAMD | 2.9184 | 3.4026 | 0.09 | 0.8577 | 0.3934 | -3.8458 | 9.6825 | 0.08 | 0.0265 | 0.6557 |
| Non-AD | MMSE | 51.738 | 18.5395 | 0.4589 | 2.7907 | 0.0084 | 14.1382 | 89.3378 | 0.2966 | 0.1989 | 0.0418* |
|  | MOCA | 37.7644 | 15.1104 | 0.4235 | 2.4992 | 0.0171 | 7.1191 | 68.4097 | 0.253 | 0.1492 | 0.0429* |
|  | ADL | -48.5245 | 22.2298 | -0.3852 | -2.1829 | 0.0356 | -93.6086 | -3.4405 | 0.1898 | 0.0773 | 0.0594 |
|  | HAMA | -2.9673 | 14.4056 | -0.0379 | -0.206 | 0.838 | -32.1833 | 26.2487 | 0.1175 | -0.0051 | 0.8711 |
|  | HAMD | -2.3104 | 14.1417 | -0.031 | -0.1634 | 0.8711 | -30.9911 | 26.3704 | 0.0633 | -0.0668 | 0.8711 |

*FDR-P≤0.05, statistically significant difference.

**Table S4.** Correlation between Whole-Brain Aβ Deposition and Regional Gray Matter Volume in AD group

| **ROI** | **B** | **SE** | **Beta** | **t** | **p** | **CI low** | **CI high** | **R^2^** | **Adj R^2^** | **FDR-P** |
| --- | --- | --- | --- | --- | --- | --- | --- | --- | --- | --- |
| lh-bankssts | -69.779 | 226.387 | -0.032 | -0.308 | 0.759 | -519.897 | 380.340 | 0.106 | 0.043 | 0.995 |
| lh-caudalanteriorcingulate | 6.075 | 264.028 | 0.002 | 0.023 | 0.982 | -518.884 | 531.033 | 0.078 | 0.013 | 0.995 |
| lh-caudalmiddlefrontal | 25.286 | 582.404 | 0.005 | 0.043 | 0.965 | -1132.690 | 1183.261 | 0.094 | 0.030 | 0.995 |
| lh-cuneus | -246.616 | 232.358 | -0.104 | -1.061 | 0.292 | -708.607 | 215.375 | 0.209 | 0.153 | 0.995 |
| lh-entorhinal | 46.435 | 230.542 | 0.021 | 0.201 | 0.841 | -411.945 | 504.814 | 0.107 | 0.044 | 0.995 |
| lh-fusiform | -340.468 | 757.661 | -0.045 | -0.449 | 0.654 | -1846.900 | 1165.965 | 0.185 | 0.128 | 0.995 |
| lh-inferiorparietal | -19.897 | 1037.085 | -0.002 | -0.019 | 0.985 | -2081.899 | 2042.106 | 0.150 | 0.090 | 0.995 |
| lh-inferiortemporal | -18.966 | 864.411 | -0.002 | -0.022 | 0.983 | -1737.646 | 1699.714 | 0.170 | 0.112 | 0.995 |
| lh-isthmuscingulate | -316.199 | 275.709 | -0.120 | -1.147 | 0.255 | -864.382 | 231.984 | 0.104 | 0.041 | 0.995 |
| lh-lateraloccipital | -648.858 | 990.460 | -0.065 | -0.655 | 0.514 | -2618.158 | 1320.441 | 0.192 | 0.135 | 0.995 |
| lh-lateralorbitofrontal | -80.906 | 378.654 | -0.020 | -0.214 | 0.831 | -833.773 | 671.960 | 0.310 | 0.261 | 0.995 |
| lh-lingual | -560.496 | 516.770 | -0.106 | -1.085 | 0.281 | -1587.973 | 466.980 | 0.208 | 0.152 | 0.995 |
| lh-medialorbitofrontal | 140.437 | 346.525 | 0.038 | 0.405 | 0.686 | -548.549 | 829.422 | 0.261 | 0.208 | 0.995 |
| lh-middletemporal | 708.648 | 914.307 | 0.081 | 0.775 | 0.440 | -1109.240 | 2526.535 | 0.107 | 0.044 | 0.995 |
| lh-parahippocampal | -174.704 | 200.037 | -0.090 | -0.873 | 0.385 | -572.432 | 223.024 | 0.133 | 0.071 | 0.995 |
| lh-paracentral | 411.943 | 219.536 | 0.178 | 1.876 | 0.064 | -24.552 | 848.439 | 0.262 | 0.210 | 0.995 |
| lh-parsopercularis | 106.702 | 414.855 | 0.027 | 0.257 | 0.798 | -718.141 | 931.545 | 0.117 | 0.055 | 0.995 |
| lh-parsorbitalis | 170.696 | 157.835 | 0.111 | 1.081 | 0.283 | -143.123 | 484.515 | 0.132 | 0.071 | 0.995 |
| lh-parstriangularis | 278.482 | 308.477 | 0.089 | 0.903 | 0.369 | -334.853 | 891.818 | 0.205 | 0.148 | 0.995 |
| lh-pericalcarine | 121.484 | 243.912 | 0.048 | 0.498 | 0.620 | -363.479 | 606.447 | 0.234 | 0.180 | 0.995 |
| lh-postcentral | 473.246 | 619.547 | 0.077 | 0.764 | 0.447 | -758.578 | 1705.071 | 0.166 | 0.107 | 0.995 |
| lh-posteriorcingulate | 305.514 | 319.893 | 0.098 | 0.955 | 0.342 | -330.518 | 941.546 | 0.134 | 0.073 | 0.995 |
| lh-precentral | 197.054 | 875.987 | 0.022 | 0.225 | 0.823 | -1544.643 | 1938.750 | 0.205 | 0.148 | 0.995 |
| lh-precuneus | -286.040 | 643.796 | -0.045 | -0.444 | 0.658 | -1566.079 | 993.999 | 0.163 | 0.104 | 0.995 |
| lh-rostralanteriorcingulate | 78.983 | 271.087 | 0.030 | 0.291 | 0.771 | -460.009 | 617.976 | 0.155 | 0.096 | 0.995 |
| lh-rostralmiddlefrontal | 6.124 | 1006.495 | 0.001 | 0.006 | 0.995 | -1995.057 | 2007.305 | 0.208 | 0.152 | 0.995 |
| lh-superiorfrontal | -586.358 | 1284.144 | -0.045 | -0.457 | 0.649 | -3139.581 | 1966.865 | 0.190 | 0.133 | 0.995 |
| lh-superiorparietal | 547.456 | 951.389 | 0.059 | 0.575 | 0.567 | -1344.160 | 2439.073 | 0.147 | 0.086 | 0.995 |
| lh-superiortemporal | -715.171 | 934.008 | -0.078 | -0.766 | 0.446 | -2572.229 | 1141.888 | 0.144 | 0.083 | 0.995 |
| lh-supramarginal | 494.347 | 1016.262 | 0.049 | 0.486 | 0.628 | -1526.254 | 2514.949 | 0.159 | 0.099 | 0.995 |
| lh-frontalpole | 56.311 | 103.695 | 0.057 | 0.543 | 0.589 | -149.863 | 262.485 | 0.081 | 0.016 | 0.995 |
| lh-temporalpole | 223.610 | 242.093 | 0.098 | 0.924 | 0.358 | -257.737 | 704.956 | 0.071 | 0.006 | 0.995 |
| lh-transversetemporal | -23.798 | 125.492 | -0.020 | -0.190 | 0.850 | -273.310 | 225.714 | 0.072 | 0.007 | 0.995 |
| lh-insula | 208.583 | 370.503 | 0.052 | 0.563 | 0.575 | -528.077 | 945.243 | 0.310 | 0.261 | 0.995 |
| rh-bankssts | -88.239 | 180.312 | -0.051 | -0.489 | 0.626 | -446.748 | 270.270 | 0.113 | 0.050 | 0.995 |
| rh-caudalanteriorcingulate | 29.144 | 269.042 | 0.011 | 0.108 | 0.914 | -505.784 | 564.072 | 0.128 | 0.066 | 0.995 |
| rh-caudalmiddlefrontal | -168.677 | 558.615 | -0.030 | -0.302 | 0.763 | -1279.355 | 942.000 | 0.193 | 0.136 | 0.995 |
| rh-cuneus | 46.410 | 259.497 | 0.018 | 0.179 | 0.858 | -469.539 | 562.360 | 0.183 | 0.125 | 0.995 |
| rh-entorhinal | -114.728 | 261.767 | -0.048 | -0.438 | 0.662 | -635.191 | 405.736 | 0.031 | -0.038 | 0.995 |
| rh-fusiform | -414.932 | 811.308 | -0.050 | -0.511 | 0.610 | -2028.029 | 1198.165 | 0.197 | 0.140 | 0.995 |
| rh-inferiorparietal | 187.297 | 1116.596 | 0.017 | 0.168 | 0.867 | -2032.795 | 2407.388 | 0.162 | 0.103 | 0.995 |
| rh-inferiortemporal | -270.399 | 917.762 | -0.029 | -0.295 | 0.769 | -2095.155 | 1554.357 | 0.196 | 0.139 | 0.995 |
| rh-isthmuscingulate | -197.024 | 212.790 | -0.096 | -0.926 | 0.357 | -620.107 | 226.059 | 0.113 | 0.050 | 0.995 |
| rh-lateraloccipital | -506.586 | 851.027 | -0.056 | -0.595 | 0.553 | -2198.655 | 1185.483 | 0.258 | 0.206 | 0.995 |
| rh-lateralorbitofrontal | 198.436 | 429.178 | 0.042 | 0.462 | 0.645 | -654.884 | 1051.756 | 0.331 | 0.283 | 0.995 |
| rh-lingual | -143.695 | 530.818 | -0.027 | -0.271 | 0.787 | -1199.105 | 911.714 | 0.187 | 0.130 | 0.995 |
| rh-medialorbitofrontal | -19.886 | 315.447 | -0.006 | -0.063 | 0.950 | -647.079 | 607.306 | 0.242 | 0.188 | 0.995 |
| rh-middletemporal | 211.922 | 933.810 | 0.022 | 0.227 | 0.821 | -1644.742 | 2068.586 | 0.218 | 0.163 | 0.995 |
| rh-parahippocampal | -188.295 | 148.691 | -0.124 | -1.266 | 0.209 | -483.932 | 107.343 | 0.204 | 0.148 | 0.995 |
| rh-paracentral | 477.279 | 261.309 | 0.178 | 1.826 | 0.071 | -42.274 | 996.833 | 0.215 | 0.160 | 0.995 |
| rh-parsopercularis | 65.360 | 326.594 | 0.020 | 0.200 | 0.842 | -583.997 | 714.717 | 0.167 | 0.108 | 0.995 |
| rh-parsorbitalis | 152.907 | 193.138 | 0.084 | 0.792 | 0.431 | -231.103 | 536.917 | 0.074 | 0.009 | 0.995 |
| rh-parstriangularis | 216.791 | 358.946 | 0.064 | 0.604 | 0.547 | -496.890 | 930.471 | 0.080 | 0.015 | 0.995 |
| rh-pericalcarine | 81.471 | 230.108 | 0.033 | 0.354 | 0.724 | -376.045 | 538.987 | 0.296 | 0.247 | 0.995 |
| rh-postcentral | 810.533 | 649.781 | 0.123 | 1.247 | 0.216 | -481.405 | 2102.472 | 0.193 | 0.136 | 0.995 |
| rh-posteriorcingulate | 263.983 | 312.889 | 0.086 | 0.844 | 0.401 | -358.124 | 886.091 | 0.154 | 0.094 | 0.995 |
| rh-precentral | 637.746 | 894.327 | 0.068 | 0.713 | 0.478 | -1140.415 | 2415.907 | 0.261 | 0.209 | 0.995 |
| rh-precuneus | 389.817 | 721.440 | 0.054 | 0.540 | 0.590 | -1044.600 | 1824.234 | 0.171 | 0.112 | 0.995 |
| rh-rostralanteriorcingulate | -159.183 | 253.289 | -0.063 | -0.628 | 0.531 | -662.789 | 344.423 | 0.184 | 0.127 | 0.995 |
| rh-rostralmiddlefrontal | 125.485 | 1149.412 | 0.011 | 0.109 | 0.913 | -2159.854 | 2410.824 | 0.231 | 0.177 | 0.995 |
| rh-superiorfrontal | 673.639 | 1376.952 | 0.049 | 0.489 | 0.626 | -2064.111 | 3411.389 | 0.164 | 0.105 | 0.995 |
| rh-superiorparietal | 430.814 | 900.934 | 0.049 | 0.478 | 0.634 | -1360.484 | 2222.111 | 0.119 | 0.057 | 0.995 |
| rh-superiortemporal | 156.761 | 753.928 | 0.021 | 0.208 | 0.836 | -1342.250 | 1655.772 | 0.166 | 0.107 | 0.995 |
| rh-supramarginal | -265.933 | 799.737 | -0.034 | -0.333 | 0.740 | -1856.025 | 1324.158 | 0.145 | 0.085 | 0.995 |
| rh-frontalpole | 126.729 | 113.633 | 0.117 | 1.115 | 0.268 | -99.203 | 352.661 | 0.095 | 0.031 | 0.995 |
| rh-temporalpole | 181.223 | 274.999 | 0.071 | 0.659 | 0.512 | -365.549 | 727.995 | 0.036 | -0.032 | 0.995 |
| rh-transversetemporal | -20.440 | 90.047 | -0.024 | -0.227 | 0.821 | -199.478 | 158.598 | 0.092 | 0.028 | 0.995 |
| rh-insula | 493.372 | 408.495 | 0.109 | 1.208 | 0.230 | -318.826 | 1305.571 | 0.335 | 0.288 | 0.995 |
| Left-Thalamus | -405.520 | 443.911 | -0.080 | -0.914 | 0.364 | -1288.134 | 477.094 | 0.375 | 0.331 | 0.995 |
| Left-Caudate | -551.345 | 296.406 | -0.172 | -1.860 | 0.066 | -1140.679 | 37.988 | 0.295 | 0.245 | 0.995 |
| Left-Putamen | -408.796 | 362.018 | -0.113 | -1.129 | 0.262 | -1128.584 | 310.993 | 0.169 | 0.111 | 0.995 |
| Left-Pallidum | 30.324 | 137.843 | 0.022 | 0.220 | 0.826 | -243.746 | 304.393 | 0.160 | 0.101 | 0.995 |
| Left-Hippocampus | -143.499 | 292.418 | -0.051 | -0.491 | 0.625 | -724.903 | 437.905 | 0.105 | 0.042 | 0.995 |
| Left-Amygdala | -102.301 | 185.740 | -0.060 | -0.551 | 0.583 | -471.601 | 267.000 | 0.020 | -0.049 | 0.995 |
| Left-Accumbens.area | -66.156 | 58.906 | -0.108 | -1.123 | 0.265 | -183.278 | 50.965 | 0.233 | 0.179 | 0.995 |
| Right-Thalamus | -241.829 | 440.653 | -0.050 | -0.549 | 0.585 | -1117.965 | 634.307 | 0.314 | 0.266 | 0.995 |
| Right-Caudate | -59.250 | 270.261 | -0.022 | -0.219 | 0.827 | -596.601 | 478.102 | 0.172 | 0.114 | 0.995 |
| Right-Putamen | -372.343 | 457.436 | -0.085 | -0.814 | 0.418 | -1281.849 | 537.162 | 0.109 | 0.046 | 0.995 |
| Right-Pallidum | 120.994 | 154.179 | 0.080 | 0.785 | 0.435 | -185.556 | 427.544 | 0.142 | 0.082 | 0.995 |
| Right-Hippocampus | 32.531 | 300.937 | 0.012 | 0.108 | 0.914 | -565.813 | 630.875 | 0.050 | -0.017 | 0.995 |
| Right-Amygdala | 28.858 | 195.997 | 0.016 | 0.147 | 0.883 | -360.836 | 418.552 | 0.028 | -0.040 | 0.995 |

**Table S5.** Correlation between Whole-Brain Aβ Deposition and Regional FDG Metabolism in AD group

| **Brain region** | **B** | **SE** | **Beta** | **t** | **p** | **CI low** | **CI high** | **R^2^** | **AdjR^2^** | **FDR-P** |
| --- | --- | --- | --- | --- | --- | --- | --- | --- | --- | --- |
| Left-Thalamus | 0.037 | 0.112 | 0.035 | 0.331 | 0.741 | -0.185 | 0.259 | 0.047 | -0.009 | 0.981 |
| Left-Caudate | 0.147 | 0.176 | 0.088 | 0.836 | 0.406 | -0.203 | 0.498 | 0.069 | 0.015 | 0.981 |
| Left-Putamen | 0.057 | 0.226 | 0.027 | 0.253 | 0.801 | -0.393 | 0.507 | 0.048 | -0.007 | 0.981 |
| Left-Pallidum | -0.068 | 0.102 | -0.069 | -0.669 | 0.505 | -0.271 | 0.134 | 0.118 | 0.067 | 0.981 |
| Left-Hippocampus | 0.016 | 0.091 | 0.019 | 0.179 | 0.858 | -0.164 | 0.197 | 0.062 | 0.007 | 0.981 |
| Left-Amygdala | -0.008 | 0.069 | -0.012 | -0.115 | 0.909 | -0.146 | 0.130 | 0.043 | -0.012 | 0.981 |
| Left-Accumbens-area | 0.034 | 0.152 | 0.024 | 0.221 | 0.825 | -0.269 | 0.336 | 0.028 | -0.028 | 0.981 |
| Right-Thalamus | 0.032 | 0.106 | 0.032 | 0.300 | 0.765 | -0.179 | 0.243 | 0.047 | -0.008 | 0.981 |
| Right-Caudate | 0.086 | 0.190 | 0.049 | 0.454 | 0.651 | -0.292 | 0.464 | 0.038 | -0.018 | 0.981 |
| Right-Putamen | 0.046 | 0.226 | 0.022 | 0.203 | 0.840 | -0.404 | 0.496 | 0.027 | -0.029 | 0.981 |
| Right-Pallidum | -0.041 | 0.116 | -0.038 | -0.355 | 0.723 | -0.272 | 0.190 | 0.049 | -0.007 | 0.981 |
| Right-Hippocampus | -0.007 | 0.094 | -0.008 | -0.076 | 0.940 | -0.194 | 0.180 | 0.052 | -0.003 | 0.981 |
| Right-Amygdala | -0.004 | 0.083 | -0.006 | -0.054 | 0.957 | -0.169 | 0.160 | 0.090 | 0.037 | 0.981 |
| lh-bankssts | -0.116 | 0.234 | -0.053 | -0.496 | 0.621 | -0.581 | 0.349 | 0.054 | -0.001 | 0.981 |
| lh-caudalanteriorcingulate | 0.095 | 0.114 | 0.088 | 0.839 | 0.404 | -0.131 | 0.322 | 0.081 | 0.028 | 0.981 |
| lh-caudalmiddlefrontal | 0.065 | 0.229 | 0.030 | 0.284 | 0.777 | -0.391 | 0.521 | 0.069 | 0.015 | 0.981 |
| lh-cuneus | -0.085 | 0.233 | -0.039 | -0.366 | 0.715 | -0.549 | 0.379 | 0.061 | 0.006 | 0.981 |
| lh-entorhinal | -0.004 | 0.104 | -0.004 | -0.038 | 0.970 | -0.211 | 0.203 | 0.036 | -0.020 | 0.981 |
| lh-fusiform | -0.138 | 0.175 | -0.083 | -0.788 | 0.433 | -0.487 | 0.211 | 0.082 | 0.029 | 0.981 |
| lh-inferiorparietal | -0.152 | 0.240 | -0.065 | -0.632 | 0.529 | -0.630 | 0.326 | 0.115 | 0.064 | 0.981 |
| lh-inferiortemporal | -0.074 | 0.190 | -0.042 | -0.392 | 0.696 | -0.453 | 0.304 | 0.057 | 0.002 | 0.981 |
| lh-isthmuscingulate | -0.040 | 0.180 | -0.024 | -0.220 | 0.827 | -0.398 | 0.319 | 0.037 | -0.019 | 0.981 |
| lh-lateraloccipital | -0.009 | 0.243 | -0.004 | -0.038 | 0.970 | -0.492 | 0.474 | 0.086 | 0.033 | 0.981 |
| lh-lateralorbitofrontal | 0.090 | 0.165 | 0.059 | 0.546 | 0.587 | -0.238 | 0.418 | 0.035 | -0.021 | 0.981 |
| lh-lingual | -0.022 | 0.201 | -0.011 | -0.110 | 0.913 | -0.422 | 0.378 | 0.095 | 0.043 | 0.981 |
| lh-medialorbitofrontal | 0.009 | 0.147 | 0.007 | 0.063 | 0.950 | -0.283 | 0.302 | 0.061 | 0.006 | 0.981 |
| lh-middletemporal | -0.110 | 0.202 | -0.056 | -0.543 | 0.588 | -0.511 | 0.292 | 0.104 | 0.052 | 0.981 |
| lh-parahippocampal | -0.156 | 0.137 | -0.119 | -1.143 | 0.256 | -0.428 | 0.116 | 0.091 | 0.038 | 0.981 |
| lh-paracentral | -0.046 | 0.177 | -0.028 | -0.257 | 0.798 | -0.398 | 0.307 | 0.035 | -0.022 | 0.981 |
| lh-parsopercularis | 0.118 | 0.206 | 0.060 | 0.570 | 0.570 | -0.292 | 0.528 | 0.064 | 0.010 | 0.981 |
| lh-parsorbitalis | 0.273 | 0.264 | 0.110 | 1.036 | 0.303 | -0.251 | 0.798 | 0.052 | -0.003 | 0.981 |
| lh-parstriangularis | 0.143 | 0.212 | 0.071 | 0.676 | 0.501 | -0.278 | 0.565 | 0.071 | 0.016 | 0.981 |
| lh-pericalcarine | -0.148 | 0.288 | -0.055 | -0.514 | 0.609 | -0.721 | 0.425 | 0.043 | -0.013 | 0.981 |
| lh-postcentral | 0.014 | 0.202 | 0.007 | 0.069 | 0.946 | -0.387 | 0.415 | 0.027 | -0.029 | 0.981 |
| lh-posteriorcingulate | -0.134 | 0.222 | -0.064 | -0.604 | 0.547 | -0.575 | 0.307 | 0.071 | 0.017 | 0.981 |
| lh-precentral | 0.034 | 0.184 | 0.020 | 0.186 | 0.853 | -0.332 | 0.400 | 0.046 | -0.010 | 0.981 |
| lh-precuneus | -0.114 | 0.227 | -0.052 | -0.500 | 0.618 | -0.565 | 0.338 | 0.094 | 0.041 | 0.981 |
| lh-rostralanteriorcingulate | 0.060 | 0.153 | 0.041 | 0.393 | 0.695 | -0.245 | 0.365 | 0.101 | 0.048 | 0.981 |
| lh-rostralmiddlefrontal | 0.037 | 0.244 | 0.016 | 0.151 | 0.881 | -0.448 | 0.521 | 0.060 | 0.006 | 0.981 |
| lh-superiorfrontal | 0.156 | 0.193 | 0.086 | 0.808 | 0.421 | -0.227 | 0.538 | 0.044 | -0.011 | 0.981 |
| lh-superiorparietal | -0.064 | 0.224 | -0.029 | -0.284 | 0.777 | -0.510 | 0.382 | 0.102 | 0.049 | 0.981 |
| lh-superiortemporal | -0.041 | 0.171 | -0.025 | -0.242 | 0.810 | -0.380 | 0.298 | 0.090 | 0.037 | 0.981 |
| lh-supramarginal | -0.030 | 0.218 | -0.015 | -0.138 | 0.891 | -0.463 | 0.403 | 0.072 | 0.018 | 0.981 |
| lh-frontalpole | 0.126 | 0.213 | 0.062 | 0.590 | 0.557 | -0.298 | 0.550 | 0.074 | 0.021 | 0.981 |
| lh-temporalpole | -0.053 | 0.156 | -0.036 | -0.339 | 0.735 | -0.363 | 0.257 | 0.063 | 0.009 | 0.981 |
| lh-transversetemporal | -0.015 | 0.305 | -0.005 | -0.050 | 0.960 | -0.622 | 0.591 | 0.034 | -0.022 | 0.981 |
| lh-insula | -0.044 | 0.183 | -0.025 | -0.238 | 0.812 | -0.407 | 0.320 | 0.083 | 0.030 | 0.981 |
| rh-bankssts | -0.187 | 0.218 | -0.090 | -0.858 | 0.393 | -0.620 | 0.246 | 0.071 | 0.017 | 0.981 |
| rh-caudalanteriorcingulate | 0.021 | 0.136 | 0.017 | 0.157 | 0.876 | -0.249 | 0.292 | 0.064 | 0.010 | 0.981 |
| rh-caudalmiddlefrontal | 0.110 | 0.236 | 0.049 | 0.467 | 0.642 | -0.359 | 0.579 | 0.082 | 0.028 | 0.981 |
| rh-cuneus | -0.083 | 0.217 | -0.040 | -0.383 | 0.703 | -0.516 | 0.349 | 0.104 | 0.052 | 0.981 |
| rh-entorhinal | -0.087 | 0.112 | -0.082 | -0.774 | 0.441 | -0.310 | 0.136 | 0.065 | 0.010 | 0.981 |
| rh-fusiform | -0.165 | 0.163 | -0.106 | -1.017 | 0.312 | -0.489 | 0.158 | 0.084 | 0.031 | 0.981 |
| rh-inferiorparietal | -0.089 | 0.232 | -0.040 | -0.386 | 0.700 | -0.550 | 0.371 | 0.086 | 0.033 | 0.981 |
| rh-inferiortemporal | -0.130 | 0.180 | -0.077 | -0.724 | 0.471 | -0.487 | 0.227 | 0.060 | 0.006 | 0.981 |
| rh-isthmuscingulate | -0.043 | 0.167 | -0.028 | -0.260 | 0.796 | -0.374 | 0.288 | 0.035 | -0.021 | 0.981 |
| rh-lateraloccipital | -0.024 | 0.225 | -0.011 | -0.106 | 0.916 | -0.470 | 0.423 | 0.062 | 0.007 | 0.981 |
| rh-lateralorbitofrontal | 0.081 | 0.160 | 0.054 | 0.506 | 0.614 | -0.238 | 0.400 | 0.035 | -0.021 | 0.981 |
| rh-lingual | -0.086 | 0.184 | -0.048 | -0.466 | 0.642 | -0.451 | 0.280 | 0.108 | 0.056 | 0.981 |
| rh-medialorbitofrontal | 0.054 | 0.156 | 0.037 | 0.345 | 0.731 | -0.256 | 0.364 | 0.060 | 0.006 | 0.981 |
| rh-middletemporal | -0.136 | 0.215 | -0.066 | -0.631 | 0.530 | -0.564 | 0.292 | 0.074 | 0.021 | 0.981 |
| rh-parahippocampal | -0.080 | 0.131 | -0.065 | -0.609 | 0.544 | -0.341 | 0.181 | 0.058 | 0.003 | 0.981 |
| rh-paracentral | -0.026 | 0.169 | -0.016 | -0.154 | 0.878 | -0.362 | 0.310 | 0.051 | -0.004 | 0.981 |
| rh-parsopercularis | 0.085 | 0.200 | 0.045 | 0.423 | 0.673 | -0.314 | 0.483 | 0.049 | -0.006 | 0.981 |
| rh-parsorbitalis | 0.266 | 0.241 | 0.118 | 1.101 | 0.274 | -0.214 | 0.745 | 0.047 | -0.008 | 0.981 |
| rh-parstriangularis | 0.124 | 0.218 | 0.060 | 0.568 | 0.572 | -0.309 | 0.556 | 0.081 | 0.027 | 0.981 |
| rh-pericalcarine | -0.092 | 0.274 | -0.035 | -0.335 | 0.739 | -0.636 | 0.453 | 0.066 | 0.011 | 0.981 |
| rh-postcentral | -0.003 | 0.191 | -0.001 | -0.014 | 0.989 | -0.382 | 0.377 | 0.035 | -0.021 | 0.989 |
| rh-posteriorcingulate | -0.150 | 0.218 | -0.072 | -0.688 | 0.494 | -0.583 | 0.283 | 0.093 | 0.041 | 0.981 |
| rh-precentral | 0.062 | 0.183 | 0.036 | 0.336 | 0.738 | -0.303 | 0.426 | 0.050 | -0.005 | 0.981 |
| rh-precuneus | -0.111 | 0.196 | -0.059 | -0.565 | 0.574 | -0.502 | 0.280 | 0.090 | 0.037 | 0.981 |
| rh-rostralanteriorcingulate | 0.032 | 0.170 | 0.019 | 0.187 | 0.852 | -0.307 | 0.370 | 0.091 | 0.038 | 0.981 |
| rh-rostralmiddlefrontal | 0.112 | 0.241 | 0.050 | 0.466 | 0.642 | -0.366 | 0.591 | 0.050 | -0.006 | 0.981 |
| rh-superiorfrontal | 0.174 | 0.196 | 0.094 | 0.884 | 0.379 | -0.217 | 0.564 | 0.059 | 0.005 | 0.981 |
| rh-superiorparietal | -0.011 | 0.211 | -0.005 | -0.051 | 0.959 | -0.429 | 0.408 | 0.102 | 0.049 | 0.981 |
| rh-superiortemporal | -0.055 | 0.159 | -0.036 | -0.347 | 0.729 | -0.372 | 0.262 | 0.102 | 0.050 | 0.981 |
| rh-supramarginal | -0.046 | 0.205 | -0.024 | -0.227 | 0.821 | -0.453 | 0.360 | 0.066 | 0.011 | 0.981 |
| rh-frontalpole | 0.188 | 0.236 | 0.085 | 0.796 | 0.428 | -0.281 | 0.658 | 0.053 | -0.002 | 0.981 |
| rh-temporalpole | -0.098 | 0.152 | -0.068 | -0.643 | 0.522 | -0.401 | 0.205 | 0.075 | 0.022 | 0.981 |
| rh-transversetemporal | 0.049 | 0.294 | 0.018 | 0.167 | 0.867 | -0.535 | 0.634 | 0.026 | -0.031 | 0.981 |
| rh-insula | -0.018 | 0.181 | -0.010 | -0.098 | 0.923 | -0.377 | 0.342 | 0.093 | 0.040 | 0.981 |

**Table S6.** Correlation between Whole-Brain Aβ Deposition and Regional Gray Matter Volume in non-AD dementia group

| **Brain region** | **B** | **SE** | **Beta** | **t** | **p** | **CI low** | **CI high** | **R^2^** | **AdjR^2^** | **FDR-P** |
| --- | --- | --- | --- | --- | --- | --- | --- | --- | --- | --- |
| lh-bankssts | 1072.816 | 907.892 | 0.196 | 1.182 | 0.245 | -770.302 | 2915.935 | 0.316 | 0.198 | 0.827 |
| lh-caudalanteriorcingulate | -1231.331 | 981.973 | -0.222 | -1.254 | 0.218 | -3224.842 | 762.180 | 0.217 | 0.083 | 0.827 |
| lh-caudalmiddlefrontal | 1196.866 | 2049.643 | 0.098 | 0.584 | 0.563 | -2964.130 | 5357.862 | 0.294 | 0.173 | 0.845 |
| lh-cuneus | 840.724 | 1123.619 | 0.120 | 0.748 | 0.459 | -1440.344 | 3121.792 | 0.364 | 0.254 | 0.827 |
| lh-entorhinal | -46.211 | 1322.268 | -0.006 | -0.035 | 0.972 | -2730.559 | 2638.137 | 0.197 | 0.060 | 0.972 |
| lh-fusiform | 2017.316 | 4261.017 | 0.083 | 0.473 | 0.639 | -6633.008 | 10667.640 | 0.233 | 0.102 | 0.845 |
| lh-inferiorparietal | 5318.540 | 3811.140 | 0.227 | 1.396 | 0.172 | -2418.487 | 13055.566 | 0.342 | 0.229 | 0.827 |
| lh-inferiortemporal | 6282.563 | 5044.118 | 0.208 | 1.246 | 0.221 | -3957.542 | 16522.668 | 0.307 | 0.189 | 0.827 |
| lh-isthmuscingulate | 824.656 | 1126.892 | 0.126 | 0.732 | 0.469 | -1463.057 | 3112.368 | 0.263 | 0.136 | 0.827 |
| lh-lateraloccipital | 4931.258 | 3150.163 | 0.227 | 1.565 | 0.126 | -1463.913 | 11326.430 | 0.478 | 0.388 | 0.827 |
| lh-lateralorbitofrontal | 4668.741 | 2299.430 | 0.344 | 2.030 | 0.050 | 0.649 | 9336.833 | 0.287 | 0.164 | 0.827 |
| lh-lingual | 1199.230 | 2477.512 | 0.088 | 0.484 | 0.631 | -3830.387 | 6228.846 | 0.173 | 0.031 | 0.845 |
| lh-medialorbitofrontal | 1054.249 | 1773.650 | 0.105 | 0.594 | 0.556 | -2546.453 | 4654.950 | 0.226 | 0.093 | 0.845 |
| lh-middletemporal | 2024.026 | 4620.588 | 0.072 | 0.438 | 0.664 | -7356.265 | 11404.318 | 0.323 | 0.207 | 0.845 |
| lh-parahippocampal | 891.942 | 1075.872 | 0.152 | 0.829 | 0.413 | -1292.194 | 3076.078 | 0.161 | 0.017 | 0.827 |
| lh-paracentral | 2346.271 | 1335.515 | 0.313 | 1.757 | 0.088 | -364.969 | 5057.511 | 0.211 | 0.075 | 0.827 |
| lh-parsopercularis | 851.323 | 1639.406 | 0.090 | 0.519 | 0.607 | -2476.849 | 4179.494 | 0.259 | 0.132 | 0.845 |
| lh-parsorbitalis | 568.422 | 590.849 | 0.175 | 0.962 | 0.343 | -631.064 | 1767.909 | 0.178 | 0.037 | 0.827 |
| lh-parstriangularis | 1819.031 | 1084.533 | 0.318 | 1.677 | 0.102 | -382.687 | 4020.749 | 0.103 | -0.050 | 0.827 |
| lh-pericalcarine | 1387.329 | 937.230 | 0.265 | 1.480 | 0.148 | -515.348 | 3290.006 | 0.203 | 0.066 | 0.827 |
| lh-postcentral | -1233.595 | 2656.365 | -0.078 | -0.464 | 0.645 | -6626.303 | 4159.112 | 0.290 | 0.169 | 0.845 |
| lh-posteriorcingulate | 1134.627 | 1212.248 | 0.169 | 0.936 | 0.356 | -1326.367 | 3595.620 | 0.191 | 0.052 | 0.827 |
| lh-precentral | 5622.476 | 3762.332 | 0.251 | 1.494 | 0.144 | -2015.464 | 13260.416 | 0.295 | 0.174 | 0.827 |
| lh-precuneus | 3524.682 | 2272.560 | 0.237 | 1.551 | 0.130 | -1088.861 | 8138.225 | 0.417 | 0.317 | 0.827 |
| lh-rostralanteriorcingulate | 1642.545 | 1339.504 | 0.217 | 1.226 | 0.228 | -1076.792 | 4361.883 | 0.218 | 0.084 | 0.827 |
| lh-rostralmiddlefrontal | 3313.202 | 3495.182 | 0.147 | 0.948 | 0.350 | -3782.394 | 10408.798 | 0.401 | 0.298 | 0.827 |
| lh-superiorfrontal | 10422.127 | 5115.385 | 0.283 | 2.037 | 0.049 | 37.344 | 20806.910 | 0.520 | 0.438 | 0.827 |
| lh-superiorparietal | 7338.840 | 3599.570 | 0.318 | 2.039 | 0.049 | 31.325 | 14646.355 | 0.394 | 0.291 | 0.827 |
| lh-superiortemporal | 2245.376 | 3939.304 | 0.093 | 0.570 | 0.572 | -5751.837 | 10242.589 | 0.332 | 0.217 | 0.845 |
| lh-supramarginal | 3735.585 | 3106.285 | 0.197 | 1.203 | 0.237 | -2570.509 | 10041.678 | 0.330 | 0.215 | 0.827 |
| lh-frontalpole | 339.034 | 353.977 | 0.153 | 0.958 | 0.345 | -379.576 | 1057.645 | 0.365 | 0.256 | 0.827 |
| lh-temporalpole | 1188.061 | 1548.021 | 0.135 | 0.767 | 0.448 | -1954.588 | 4330.710 | 0.228 | 0.096 | 0.827 |
| lh-transversetemporal | -312.756 | 519.586 | -0.115 | -0.602 | 0.551 | -1367.572 | 742.061 | 0.084 | -0.073 | 0.845 |
| lh-insula | 1258.167 | 2144.246 | 0.094 | 0.587 | 0.561 | -3094.884 | 5611.219 | 0.367 | 0.259 | 0.845 |
| rh-bankssts | -490.537 | 878.594 | -0.100 | -0.558 | 0.580 | -2274.177 | 1293.103 | 0.195 | 0.058 | 0.845 |
| rh-caudalanteriorcingulate | 419.647 | 1018.946 | 0.081 | 0.412 | 0.683 | -1648.924 | 2488.217 | 0.027 | -0.140 | 0.848 |
| rh-caudalmiddlefrontal | -2480.055 | 2293.880 | -0.182 | -1.081 | 0.287 | -7136.880 | 2176.769 | 0.296 | 0.175 | 0.827 |
| rh-cuneus | -131.977 | 1348.550 | -0.018 | -0.098 | 0.923 | -2869.680 | 2605.725 | 0.189 | 0.050 | 0.961 |
| rh-entorhinal | 599.445 | 1105.700 | 0.100 | 0.542 | 0.591 | -1645.245 | 2844.136 | 0.159 | 0.015 | 0.845 |
| rh-fusiform | 3377.646 | 3423.676 | 0.154 | 0.987 | 0.331 | -3572.785 | 10328.077 | 0.397 | 0.294 | 0.827 |
| rh-inferiorparietal | -4519.075 | 4618.905 | -0.163 | -0.978 | 0.335 | -13895.950 | 4857.800 | 0.312 | 0.194 | 0.827 |
| rh-inferiortemporal | 1510.593 | 3759.531 | 0.062 | 0.402 | 0.690 | -6121.661 | 9142.847 | 0.414 | 0.313 | 0.848 |
| rh-isthmuscingulate | 438.495 | 1013.440 | 0.076 | 0.433 | 0.668 | -1618.898 | 2495.887 | 0.241 | 0.111 | 0.845 |
| rh-lateraloccipital | 3001.744 | 3625.966 | 0.127 | 0.828 | 0.413 | -4359.358 | 10362.847 | 0.415 | 0.315 | 0.827 |
| rh-lateralorbitofrontal | 2181.928 | 2157.680 | 0.167 | 1.011 | 0.319 | -2198.395 | 6562.250 | 0.320 | 0.204 | 0.827 |
| rh-lingual | -777.765 | 2371.836 | -0.060 | -0.328 | 0.745 | -5592.848 | 4037.317 | 0.177 | 0.036 | 0.878 |
| rh-medialorbitofrontal | 1978.148 | 1572.430 | 0.220 | 1.258 | 0.217 | -1214.055 | 5170.351 | 0.240 | 0.109 | 0.827 |
| rh-middletemporal | -757.150 | 3735.124 | -0.033 | -0.203 | 0.841 | -8339.855 | 6825.554 | 0.351 | 0.239 | 0.927 |
| rh-parahippocampal | -1041.821 | 743.339 | -0.224 | -1.402 | 0.170 | -2550.879 | 467.237 | 0.366 | 0.258 | 0.827 |
| rh-paracentral | 2306.173 | 1405.702 | 0.278 | 1.641 | 0.110 | -547.553 | 5159.900 | 0.282 | 0.159 | 0.827 |
| rh-parsopercularis | 899.780 | 1251.994 | 0.114 | 0.719 | 0.477 | -1641.903 | 3441.463 | 0.371 | 0.263 | 0.827 |
| rh-parsorbitalis | 856.922 | 937.894 | 0.169 | 0.914 | 0.367 | -1047.104 | 2760.947 | 0.147 | 0.001 | 0.827 |
| rh-parstriangularis | 96.535 | 1486.448 | 0.011 | 0.065 | 0.949 | -2921.115 | 3114.186 | 0.230 | 0.098 | 0.962 |
| rh-pericalcarine | 1622.727 | 1147.650 | 0.256 | 1.414 | 0.166 | -707.126 | 3952.580 | 0.183 | 0.043 | 0.827 |
| rh-postcentral | -752.438 | 2910.158 | -0.040 | -0.259 | 0.797 | -6660.373 | 5155.497 | 0.391 | 0.287 | 0.903 |
| rh-posteriorcingulate | 1267.976 | 1269.897 | 0.179 | 0.998 | 0.325 | -1310.053 | 3846.005 | 0.197 | 0.059 | 0.827 |
| rh-precentral | 5235.978 | 4644.575 | 0.202 | 1.127 | 0.267 | -4193.010 | 14664.966 | 0.204 | 0.067 | 0.827 |
| rh-precuneus | 6037.508 | 2516.186 | 0.342 | 2.399 | 0.022 | 929.380 | 11145.637 | 0.495 | 0.409 | 0.827 |
| rh-rostralanteriorcingulate | -171.925 | 943.526 | -0.033 | -0.182 | 0.856 | -2087.385 | 1743.535 | 0.178 | 0.038 | 0.932 |
| rh-rostralmiddlefrontal | -2345.968 | 5420.154 | -0.078 | -0.433 | 0.668 | -13349.465 | 8657.529 | 0.188 | 0.049 | 0.845 |
| rh-superiorfrontal | 8198.274 | 4691.403 | 0.219 | 1.748 | 0.089 | -1325.780 | 17722.328 | 0.609 | 0.542 | 0.827 |
| rh-superiorparietal | 1976.307 | 4094.278 | 0.087 | 0.483 | 0.632 | -6335.519 | 10288.134 | 0.184 | 0.044 | 0.845 |
| rh-superiortemporal | -2515.528 | 3049.094 | -0.121 | -0.825 | 0.415 | -8705.517 | 3674.461 | 0.464 | 0.372 | 0.827 |
| rh-supramarginal | 1961.649 | 2718.517 | 0.119 | 0.722 | 0.475 | -3557.235 | 7480.533 | 0.321 | 0.205 | 0.827 |
| rh-frontalpole | 163.968 | 433.530 | 0.061 | 0.378 | 0.708 | -716.145 | 1044.081 | 0.349 | 0.238 | 0.857 |
| rh-temporalpole | 983.307 | 1547.020 | 0.115 | 0.636 | 0.529 | -2157.310 | 4123.924 | 0.190 | 0.051 | 0.845 |
| rh-transversetemporal | -330.108 | 430.976 | -0.141 | -0.766 | 0.449 | -1205.035 | 544.819 | 0.152 | 0.006 | 0.827 |
| rh-insula | -641.920 | 1790.117 | -0.054 | -0.359 | 0.722 | -4276.050 | 2992.211 | 0.435 | 0.339 | 0.862 |
| Left-Thalamus | 388.922 | 1681.470 | 0.035 | 0.231 | 0.818 | -3024.644 | 3802.488 | 0.413 | 0.313 | 0.914 |
| Left-Caudate | 2629.629 | 1968.798 | 0.244 | 1.336 | 0.190 | -1367.244 | 6626.503 | 0.172 | 0.030 | 0.827 |
| Left-Putamen | 2057.702 | 2080.423 | 0.178 | 0.989 | 0.329 | -2165.782 | 6281.185 | 0.195 | 0.057 | 0.827 |
| Left-Pallidum | -70.080 | 626.371 | -0.018 | -0.112 | 0.912 | -1341.681 | 1201.521 | 0.322 | 0.205 | 0.961 |
| Left-Hippocampus | 1310.182 | 1638.976 | 0.135 | 0.799 | 0.429 | -2017.116 | 4637.480 | 0.286 | 0.163 | 0.827 |
| Left-Amygdala | 920.881 | 1091.751 | 0.145 | 0.843 | 0.405 | -1295.491 | 3137.252 | 0.267 | 0.141 | 0.827 |
| Right-Thalamus | 1897.999 | 1890.694 | 0.155 | 1.004 | 0.322 | -1940.313 | 5736.312 | 0.407 | 0.305 | 0.827 |
| Right-Caudate | 2199.971 | 1622.432 | 0.239 | 1.356 | 0.184 | -1093.741 | 5493.684 | 0.223 | 0.090 | 0.827 |
| Right-Putamen | 933.960 | 1928.880 | 0.084 | 0.484 | 0.631 | -2981.875 | 4849.795 | 0.243 | 0.113 | 0.845 |
| Right-Pallidum | -575.129 | 466.369 | -0.183 | -1.233 | 0.226 | -1521.908 | 371.651 | 0.449 | 0.354 | 0.827 |
| Right-Hippocampus | -146.751 | 1595.360 | -0.016 | -0.092 | 0.927 | -3385.504 | 3092.003 | 0.202 | 0.065 | 0.961 |
| Right-Amygdala | -285.687 | 970.067 | -0.054 | -0.295 | 0.770 | -2255.028 | 1683.655 | 0.165 | 0.022 | 0.895 |

**Table S7.** Correlation between Whole-Brain Aβ Deposition and Regional FDG Metabolism in non-AD dementia group

| **ROI** | **B** | **SE** | **Beta** | **t** | **p** | **CI low** | **CI high** | **R^2^** | **Adj R^2^** | **FDR-P** |
| --- | --- | --- | --- | --- | --- | --- | --- | --- | --- | --- |
| Left-Thalamus | 0.406457094 | 0.476585901 | 0.152720865 | 0.852851697 | 0.399380332 | -0.560103912 | 1.373018101 | 0.165798263 | 0.04993691 | 0.97661638 |
| Left-Caudate | 0.819251275 | 0.787431416 | 0.182684149 | 1.04040969 | 0.305086906 | -0.777733656 | 2.416236205 | 0.197926196 | 0.086527057 | 0.97661638 |
| Left-Putamen | 0.12079362 | 0.698703672 | 0.030080797 | 0.172882475 | 0.863712162 | -1.296243104 | 1.537830345 | 0.212413772 | 0.103026796 | 0.97661638 |
| Left-Pallidum | 0.261927782 | 0.359403968 | 0.131820521 | 0.72878378 | 0.470848698 | -0.466977249 | 0.990832813 | 0.148881504 | 0.030670602 | 0.97661638 |
| Left-Hippocampus | -0.298170366 | 0.397617687 | -0.128440561 | -0.74989211 | 0.458192665 | -1.104576412 | 0.508235679 | 0.236817863 | 0.130820345 | 0.97661638 |
| Left-Amygdala | -0.0279294 | 0.361001092 | -0.013376743 | -0.077366526 | 0.938760374 | -0.760073549 | 0.704214749 | 0.222293089 | 0.114278241 | 0.97812632 |
| Right-Thalamus | 0.195381672 | 0.461014562 | 0.077578282 | 0.423808028 | 0.674227477 | -0.739599196 | 1.130362541 | 0.128307441 | 0.00723903 | 0.97661638 |
| Right-Caudate | 0.160871599 | 0.75831208 | 0.036810173 | 0.212144318 | 0.833191031 | -1.377056582 | 1.69879978 | 0.216761052 | 0.107977864 | 0.97661638 |
| Right-Putamen | 0.127036457 | 0.811202662 | 0.02837359 | 0.156602614 | 0.876432997 | -1.518158795 | 1.77223171 | 0.146011686 | 0.027402198 | 0.97661638 |
| Right-Pallidum | -0.091134813 | 0.487173457 | -0.035035356 | -0.187068511 | 0.852657228 | -1.079168378 | 0.896898753 | 0.087499573 | -0.039236598 | 0.97661638 |
| Right-Hippocampus | -0.713144354 | 0.408649872 | -0.307701138 | -1.745123156 | 0.089495312 | -1.541924709 | 0.115636 | 0.19122556 | 0.078895776 | 0.97661638 |
| Right-Amygdala | -0.222803789 | 0.308571912 | -0.124856563 | -0.722048186 | 0.474929138 | -0.848616633 | 0.403009054 | 0.222121501 | 0.114082821 | 0.97661638 |
| lh-bankssts | -0.099233502 | 0.802769993 | -0.022273084 | -0.123613865 | 0.902308612 | -1.727326509 | 1.528859505 | 0.155406556 | 0.03810191 | 0.97661638 |
| lh-caudalanteriorcingulate | 0.876412719 | 0.566463281 | 0.277731731 | 1.547165982 | 0.13057014 | -0.272428064 | 2.025253501 | 0.161701748 | 0.045271436 | 0.97661638 |
| lh-caudalmiddlefrontal | 1.591736881 | 0.941304475 | 0.278716377 | 1.690990453 | 0.099480042 | -0.317317079 | 3.50079084 | 0.293252891 | 0.195093571 | 0.97661638 |
| lh-cuneus | 0.298010932 | 0.751850564 | 0.063015168 | 0.396369898 | 0.694171938 | -1.226812687 | 1.822834551 | 0.342478061 | 0.251155569 | 0.97661638 |
| lh-entorhinal | 0.455539221 | 0.669724605 | 0.12252322 | 0.680188868 | 0.500736055 | -0.902725232 | 1.813803674 | 0.155889823 | 0.038652298 | 0.97661638 |
| lh-fusiform | 0.106352112 | 0.817903004 | 0.02248217 | 0.130030225 | 0.89726637 | -1.552432064 | 1.765136288 | 0.22230518 | 0.114292011 | 0.97661638 |
| lh-inferiorparietal | 0.64369324 | 1.066692616 | 0.109419974 | 0.603447732 | 0.54999441 | -1.519659656 | 2.807046135 | 0.14466691 | 0.025870647 | 0.97661638 |
| lh-inferiortemporal | -0.324829105 | 0.864482339 | -0.064540194 | -0.375749845 | 0.709308013 | -2.07808055 | 1.42842234 | 0.232489571 | 0.1258909 | 0.97661638 |
| lh-isthmuscingulate | 0.568483496 | 0.823823742 | 0.111664366 | 0.690054761 | 0.494584589 | -1.102308494 | 2.239275485 | 0.318786151 | 0.224173116 | 0.97661638 |
| lh-lateraloccipital | 1.067075181 | 0.973077808 | 0.191580325 | 1.096598002 | 0.280098699 | -0.906418085 | 3.040568446 | 0.205985766 | 0.095706011 | 0.97661638 |
| lh-lateralorbitofrontal | 0.779360457 | 0.840507767 | 0.170586022 | 0.92724956 | 0.35997221 | -0.925268302 | 2.483989216 | 0.119529141 | -0.002758478 | 0.97661638 |
| lh-lingual | -0.369647462 | 0.606086681 | -0.099795986 | -0.609892072 | 0.545764035 | -1.598848224 | 0.859553301 | 0.303467238 | 0.206726576 | 0.97661638 |
| lh-medialorbitofrontal | 0.600178892 | 0.824759817 | 0.129532352 | 0.727701423 | 0.47150303 | -1.072511545 | 2.27286933 | 0.175726329 | 0.061243875 | 0.97661638 |
| lh-middletemporal | 0.220602705 | 0.81282868 | 0.048916908 | 0.271401231 | 0.787632751 | -1.427890265 | 1.869095675 | 0.154885138 | 0.037508074 | 0.97661638 |
| lh-parahippocampal | -0.370018293 | 0.808936217 | -0.07975964 | -0.457413433 | 0.650122538 | -2.010616982 | 1.270580397 | 0.209011955 | 0.099152505 | 0.97661638 |
| lh-paracentral | 0.612688453 | 0.432982014 | 0.234826827 | 1.415043657 | 0.165648142 | -0.265439772 | 1.490816677 | 0.283564466 | 0.184059531 | 0.97661638 |
| lh-parsopercularis | 1.350707032 | 0.950975428 | 0.255629037 | 1.420338521 | 0.164112473 | -0.577960528 | 3.279374592 | 0.157329073 | 0.040291444 | 0.97661638 |
| lh-parsorbitalis | 1.744794406 | 1.157144076 | 0.275878361 | 1.507845429 | 0.140320556 | -0.602002553 | 4.091591366 | 0.129150708 | 0.008199417 | 0.97661638 |
| lh-parstriangularis | 0.944522769 | 0.939625309 | 0.177121551 | 1.005212142 | 0.321503768 | -0.961125683 | 2.850171222 | 0.192302499 | 0.080122291 | 0.97661638 |
| lh-pericalcarine | -0.418910963 | 1.076760606 | -0.06748874 | -0.389047445 | 0.699532806 | -2.602682687 | 1.764860762 | 0.217149421 | 0.108420174 | 0.97661638 |
| lh-postcentral | 0.971123485 | 0.663782636 | 0.259307393 | 1.463014295 | 0.152140216 | -0.375090096 | 2.317337066 | 0.182751683 | 0.069244972 | 0.97661638 |
| lh-posteriorcingulate | -0.065824906 | 0.970192224 | -0.012076008 | -0.067847283 | 0.946282642 | -2.033465935 | 1.901816122 | 0.175855682 | 0.061391194 | 0.97812632 |
| lh-precentral | 1.038873599 | 0.535964691 | 0.323719148 | 1.938324699 | 0.060456044 | -0.048113176 | 2.125860374 | 0.274386934 | 0.173607342 | 0.97661638 |
| lh-precuneus | 0.824429742 | 0.862937157 | 0.160695304 | 0.955376339 | 0.345759212 | -0.92568793 | 2.574547414 | 0.263998354 | 0.161775903 | 0.97661638 |
| lh-rostralanteriorcingulate | 0.120255398 | 0.844159121 | 0.026164515 | 0.142455842 | 0.887514272 | -1.59177865 | 1.832289446 | 0.122421016 | 0.000535046 | 0.97661638 |
| lh-rostralmiddlefrontal | 1.258682936 | 0.913425441 | 0.240627471 | 1.377981036 | 0.176714662 | -0.593829722 | 3.111195594 | 0.206722185 | 0.096544711 | 0.97661638 |
| lh-superiorfrontal | 1.518475723 | 0.740627034 | 0.340999075 | 2.050256949 | 0.047675431 | 0.016414478 | 3.020536967 | 0.280366797 | 0.180417741 | 0.97661638 |
| lh-superiorparietal | 0.662497023 | 0.8744844 | 0.127192702 | 0.757585867 | 0.45362957 | -1.111039544 | 2.436033589 | 0.266699407 | 0.164852102 | 0.97661638 |
| lh-superiortemporal | 0.406882875 | 0.780588808 | 0.093325591 | 0.521251228 | 0.605383765 | -1.176224604 | 1.989990354 | 0.166071745 | 0.050248376 | 0.97661638 |
| lh-supramarginal | 0.390494712 | 0.810996089 | 0.085068659 | 0.481500118 | 0.633075186 | -1.254281591 | 2.035271015 | 0.187978071 | 0.075197248 | 0.97661638 |
| lh-frontalpole | 1.435038198 | 0.993227192 | 0.254961632 | 1.444823712 | 0.157155963 | -0.579319911 | 3.449396308 | 0.189894993 | 0.077380408 | 0.97661638 |
| lh-temporalpole | -0.057036848 | 1.10844624 | -0.009216273 | -0.051456576 | 0.959246167 | -2.305070017 | 2.190996321 | 0.165453929 | 0.049544753 | 0.97812632 |
| lh-transversetemporal | 0.927967561 | 1.499227374 | 0.11390046 | 0.618963859 | 0.539837509 | -2.112606481 | 3.968541604 | 0.119069299 | -0.003282188 | 0.97661638 |
| lh-insula | 0.024152235 | 1.19274224 | 0.00369576 | 0.020249333 | 0.983956293 | -2.394841147 | 2.443145617 | 0.13342254 | 0.01306456 | 0.983956293 |
| rh-bankssts | -0.307718816 | 0.738671239 | -0.070652686 | -0.416584266 | 0.679455987 | -1.805813525 | 1.190375893 | 0.251704723 | 0.147774823 | 0.97661638 |
| rh-caudalanteriorcingulate | 0.989622253 | 0.550749276 | 0.306871291 | 1.796865283 | 0.080751275 | -0.127349049 | 2.106593555 | 0.241242738 | 0.135859785 | 0.97661638 |
| rh-caudalmiddlefrontal | 1.201948354 | 0.978354021 | 0.199570967 | 1.228541334 | 0.227214891 | -0.782245567 | 3.186142274 | 0.313506302 | 0.218159955 | 0.97661638 |
| rh-cuneus | 0.192690462 | 0.771985954 | 0.041925987 | 0.249603585 | 0.804312708 | -1.37296962 | 1.758350543 | 0.266016445 | 0.164074285 | 0.97661638 |
| rh-entorhinal | -0.401440746 | 0.494808195 | -0.142062597 | -0.811305775 | 0.422521885 | -1.404958279 | 0.602076786 | 0.202351154 | 0.091566592 | 0.97661638 |
| rh-fusiform | -0.29355473 | 0.767251601 | -0.065225157 | -0.382605562 | 0.704261948 | -1.849613099 | 1.262503639 | 0.243952505 | 0.138945908 | 0.97661638 |
| rh-inferiorparietal | 0.597895009 | 1.04699593 | 0.101894321 | 0.571057625 | 0.571508561 | -1.525511155 | 2.721301174 | 0.171749969 | 0.056715242 | 0.97661638 |
| rh-inferiortemporal | -0.662993543 | 0.799040088 | -0.139651075 | -0.829737523 | 0.412155666 | -2.283521953 | 0.957534866 | 0.263066191 | 0.160714274 | 0.97661638 |
| rh-isthmuscingulate | 0.3588452 | 0.873328713 | 0.069858537 | 0.410893624 | 0.683586196 | -1.412347524 | 2.130037924 | 0.248028219 | 0.143587694 | 0.97661638 |
| rh-lateraloccipital | 1.030398777 | 0.983976547 | 0.184479657 | 1.047178187 | 0.301997544 | -0.965198155 | 3.025995709 | 0.192621526 | 0.080485627 | 0.97661638 |
| rh-lateralorbitofrontal | 0.432713002 | 0.699753496 | 0.111514791 | 0.618379193 | 0.540218452 | -0.986452866 | 1.85187887 | 0.153987873 | 0.036486189 | 0.97661638 |
| rh-lingual | -0.255611813 | 0.675946865 | -0.064029097 | -0.378153707 | 0.707537152 | -1.626495595 | 1.115271969 | 0.254170773 | 0.15058338 | 0.97661638 |
| rh-medialorbitofrontal | 0.404375663 | 0.654710359 | 0.107126649 | 0.617640545 | 0.540699924 | -0.923438488 | 1.732189814 | 0.217391035 | 0.108695345 | 0.97661638 |
| rh-middletemporal | -0.055056289 | 0.734939568 | -0.01312968 | -0.074912675 | 0.940698932 | -1.545582819 | 1.43547024 | 0.200867021 | 0.08987633 | 0.97812632 |
| rh-parahippocampal | -0.13284835 | 0.661866489 | -0.03465589 | -0.200717747 | 0.842048981 | -1.475175805 | 1.209479105 | 0.224460482 | 0.11674666 | 0.97661638 |
| rh-paracentral | 0.640838693 | 0.516754203 | 0.215950485 | 1.240122847 | 0.222952439 | -0.407187406 | 1.688864792 | 0.211139291 | 0.101575304 | 0.97661638 |
| rh-parsopercularis | 1.200719921 | 0.880031742 | 0.241408246 | 1.364405241 | 0.180909049 | -0.584067175 | 2.985507017 | 0.185598027 | 0.072486642 | 0.97661638 |
| rh-parsorbitalis | 0.92750841 | 1.117194739 | 0.151599207 | 0.830211938 | 0.411890939 | -1.338267538 | 3.193284358 | 0.132564307 | 0.012087128 | 0.97661638 |
| rh-parstriangularis | 0.753317967 | 0.842107473 | 0.154087821 | 0.894562738 | 0.376963011 | -0.954555147 | 2.46119108 | 0.228143804 | 0.120941554 | 0.97661638 |
| rh-pericalcarine | -0.803772443 | 1.102462889 | -0.127755409 | -0.72906984 | 0.47067585 | -3.039670814 | 1.432125929 | 0.20119329 | 0.090247913 | 0.97661638 |
| rh-postcentral | 0.90429072 | 0.768023178 | 0.212015454 | 1.17742634 | 0.246751141 | -0.65333248 | 2.46191392 | 0.156492557 | 0.039338746 | 0.97661638 |
| rh-posteriorcingulate | -0.306334875 | 0.919814267 | -0.058640891 | -0.333039925 | 0.741035997 | -2.171804672 | 1.559134922 | 0.193452587 | 0.081432113 | 0.97661638 |
| rh-precentral | 0.82222515 | 0.57953908 | 0.237532821 | 1.418757041 | 0.164569975 | -0.353134581 | 1.99758488 | 0.270790079 | 0.169510924 | 0.97661638 |
| rh-precuneus | 0.435873101 | 0.887281374 | 0.084290672 | 0.491245633 | 0.626234403 | -1.363616932 | 2.235363133 | 0.234080712 | 0.127703033 | 0.97661638 |
| rh-rostralanteriorcingulate | 0.218998813 | 0.767653631 | 0.052422788 | 0.285283368 | 0.77706201 | -1.337874911 | 1.775872536 | 0.121566873 | -0.000437728 | 0.97661638 |
| rh-rostralmiddlefrontal | 0.815060206 | 0.911137369 | 0.154069739 | 0.894552493 | 0.376968416 | -1.032812027 | 2.662932438 | 0.228307267 | 0.121127721 | 0.97661638 |
| rh-superiorfrontal | 1.295652381 | 0.779412736 | 0.27487238 | 1.662344379 | 0.105128151 | -0.285069914 | 2.876374676 | 0.288718386 | 0.189929272 | 0.97661638 |
| rh-superiorparietal | 0.515071637 | 0.888797475 | 0.098754857 | 0.579515189 | 0.565850804 | -1.28749319 | 2.317636463 | 0.244544893 | 0.139620572 | 0.97661638 |
| rh-superiortemporal | 0.157350187 | 0.753288745 | 0.035889735 | 0.208884293 | 0.835716028 | -1.370390198 | 1.685090571 | 0.232019278 | 0.125355288 | 0.97661638 |
| rh-supramarginal | 0.501429664 | 0.746441734 | 0.111759797 | 0.671759953 | 0.50602486 | -1.012424339 | 2.015283667 | 0.279947167 | 0.17993983 | 0.97661638 |
| rh-frontalpole | 1.166092283 | 0.983174539 | 0.207144821 | 1.186048089 | 0.24337249 | -0.827878102 | 3.160062667 | 0.206467096 | 0.096254193 | 0.97661638 |
| rh-temporalpole | -0.632723158 | 0.98738612 | -0.1148582 | -0.640806211 | 0.525707065 | -2.635235025 | 1.369788708 | 0.164220018 | 0.048139465 | 0.97661638 |
| rh-transversetemporal | 0.837386889 | 1.387185482 | 0.109049518 | 0.60365892 | 0.54985551 | -1.975955665 | 3.650729443 | 0.151043122 | 0.033132445 | 0.97661638 |
| rh-insula | -0.13808398 | 0.98899747 | -0.025375953 | -0.139620155 | 0.889738322 | -2.143863815 | 1.867695856 | 0.140650448 | 0.021296344 | 0.97661638 |

### Exploratory Analysis of ApoE ε4-Related Differences in Cognitive Performance and Brain Imaging Biomarkers in AD

Of the 134 participants, 71 (53.0%) underwent ApoE genotyping, while 63 (47.0%) did not. No significant differences in clinical features, cognitive scale scores, regional GMV, FDG SUVR, or AV45 SUVR were detected between genotyped and non-genotyped subgroups (Table S12-S15). Among the 71 participants with available ApoE genotyping, 53 were diagnosed with AD and 18 were classified as non-AD. Within the AD group, 20 participants were heterozygotes, 9 were homozygotes, and 24 were non-carriers. No significant differences were observed among these three genotype groups in demographic characteristics or neuropsychological measures (Table S8). Furthermore, no significant genotype-related differences were detected in regional GMV, FDG metabolism, or Aβ deposition after FDR correction (Table S9-S11). Genotype-group comparisons were not conducted in the non-AD group because only one participant had the ApoE ε4/ε4 genotype, resulting in an insufficient sample size for meaningful statistical analysis.

**Table S8. Comparison of Clinical Data and Neuropsychological Scale Scores among ApoE ε4 Homozygous, Heterozygous, and Non-Carrier in AD group**

| **Indicators** | **ApoE ε4 homozygous group** | **ApoE ε4 heterozygous group** | **Non-carrier group** | **F** | ***R^2^*** | ***Adj.R^2^*** | **P value** |
| --- | --- | --- | --- | --- | --- | --- | --- |
| Age (years) | 70.45 ± 5.15 | 70.44 ± 10.91 | 69.88 ± 6.80 | 0.043 | 0.002 | -0.038 | 0.958 |
| Male [n (%)] | 5/9 (55.6%) | 4/20 (20.0%) | 7/24 (29.2%) | – | – | – | 0.194 |
| Disease duration (years) | 5.00 (3.00, 6.00) | 3.00 (2.00, 6.00) | 2.50 (2.00, 4.25) | 1.237 | 0.154 | 0.084 | 0.107 |
| Educational years (years) | 12.00 (9.00, 12.00) | 9.00 (9.00, 12.00) | 11.00 (8.75, 12.75) | 0.227 | 0.041 | -0.039 | 0.555 |
| MMSE score(points) | 17.00 (15.00, 23.00) | 14.00 (11.00, 21.00) | 18.00 (15.00, 22.00) | 1.086 | 0.094 | -0.024 | 0.526 |
| MoCA score(points) | 14.22 ± 6.20 | 11.85 ± 5.63 | 11.92 ± 6.58 | 1.553 | 0.153 | 0.042 | 0.589 |
| ADL score(points) | 24.00 (18.00, 39.00) | 29.50 (24.75, 42.00) | 22.00 (16.75, 27.25) | 2.144 | 0.154 | 0.043 | 0.051 |
| HAMA score(points) | 7.00 (3.00, 14.00) | 15.50 (8.50, 19.75) | 6.50 (5.75, 11.25) | 4.045 | 0.193 | 0.087 | 0.566 |
| HAMD score(points) | 11.00 ± 5.92 | 11.60 ± 6.18 | 7.96 ± 4.02 | 2.166 | 0.196 | 0.092 | 0.065 |
|  |  |  |  |  |  |  |  |

**Table S9. Comparison of Gray Matter Volume among ApoE ε4 Homozygous, Heterozygous, and Non-Carrier in AD group**

| **Brain region** | **Heterozygous** | **Homozygous** | **Non-carrier** | **F value** | **P value** | **FDR-P value** |
| --- | --- | --- | --- | --- | --- | --- |
| Left-Amygdala | 1277.67±308.37 | 1448.07±226.83 | 1211.12±330.07 | 0.838 | 0.439 | 0.666 |
| Left-Caudate | 3021.27±426.97 | 3183.52±391.42 | 2963.71±502.39 | 2.171 | 0.126 | 0.537 |
| Left-Hippocampus | 3037.07±417.34 | 2909.33±299.53 | 2902.68±548.90 | 0.055 | 0.946 | 0.946 |
| Left-Pallidum | 1858.78±219.40 | 1844.50±278.76 | 1817.55±267.81 | 5.136 | 0.010 | 0.237 |
| Left-Putamen | 4153.83±501.53 | 4366.67±674.88 | 3878.82±668.31 | 4.886 | 0.012 | 0.237 |
| Left-Thalamus | 5933.68±727.27 | 6487.74±1360.98 | 5956.90±954.06 | 5.141 | 0.010 | 0.237 |
| Right-Amygdala | 1397.44±325.69 | 1511.83±236.21 | 1280.85±336.37 | 0.288 | 0.751 | 0.855 |
| Right-Caudate | 3115.66±476.61 | 3329.33±434.74 | 2994.82±456.49 | 3.316 | 0.045 | 0.454 |
| Right-Hippocampus | 3154.16±460.72 | 2984.91±262.41 | 3037.19±543.95 | 5.642 | 0.007 | 0.237 |
| Right-Pallidum | 1792.98±247.35 | 1797.86±274.37 | 1742.69±233.93 | 0.132 | 0.877 | 0.893 |
| Right-Putamen | 4343.77±544.40 | 4617.98±635.49 | 4066.85±812.94 | 0.621 | 0.542 | 0.748 |
| Right-Thalamus | 6248.70±838.85 | 6621.66±1247.24 | 6283.40±863.18 | 0.565 | 0.572 | 0.766 |
| lh-bankssts | 1696.65±374.97 | 1882.56±367.95 | 1624.92±437.14 | 1.230 | 0.302 | 0.567 |
| lh-caudalanteriorcingulate | 1535.25±565.51 | 1231.11±188.95 | 1247.17±339.38 | 1.176 | 0.318 | 0.567 |
| lh-caudalmiddlefrontal | 4821.55±991.65 | 5037.44±1332.81 | 4617.83±872.81 | 0.449 | 0.641 | 0.833 |
| lh-cuneus | 2690.55±438.60 | 2522.78±493.69 | 2736.13±360.87 | 1.803 | 0.176 | 0.537 |
| lh-entorhinal | 1355.90±391.97 | 1391.67±547.47 | 1343.17±389.05 | 0.182 | 0.834 | 0.882 |
| lh-frontalpole | 872.85±141.46 | 1041.67±139.21 | 892.83±168.83 | 1.303 | 0.282 | 0.567 |
| lh-fusiform | 7754.65±820.94 | 9031.56±1489.21 | 7394.67±1484.00 | 1.907 | 0.160 | 0.537 |
| lh-inferiorparietal | 9586.05±1546.47 | 10610.44±1459.77 | 8925.67±1609.31 | 1.028 | 0.366 | 0.585 |
| lh-inferiortemporal | 8844.35±1428.35 | 9284.56±1054.71 | 8091.21±1313.88 | 3.701 | 0.032 | 0.370 |
| lh-insula | 6153.15±561.73 | 6249.56±593.09 | 5884.17±726.23 | 0.883 | 0.421 | 0.649 |
| lh-isthmuscingulate | 2185.75±601.89 | 2171.56±177.42 | 2240.29±404.60 | 0.805 | 0.453 | 0.676 |
| lh-lateraloccipital | 10383.80±1647.14 | 10894.89±959.34 | 9831.88±1698.75 | 1.765 | 0.183 | 0.537 |
| lh-lateralorbitofrontal | 6384.00±812.83 | 6410.78±715.33 | 6217.08±676.68 | 1.106 | 0.340 | 0.573 |
| lh-lingual | 5621.90±1099.02 | 5431.11±795.18 | 5632.17±880.75 | 0.148 | 0.863 | 0.892 |
| lh-medialorbitofrontal | 4648.05±575.50 | 4896.11±648.91 | 4360.67±627.26 | 1.140 | 0.329 | 0.572 |
| lh-middletemporal | 8376.45±1300.53 | 9744.33±980.90 | 8040.38±1548.87 | 1.218 | 0.305 | 0.567 |
| lh-paracentral | 3136.40±414.17 | 3244.44±443.66 | 3297.21±405.36 | 0.717 | 0.494 | 0.711 |
| lh-parahippocampal | 1588.35±459.85 | 1591.22±263.95 | 1602.83±319.34 | 2.169 | 0.126 | 0.537 |
| lh-parsopercularis | 3720.40±751.73 | 3783.00±788.72 | 3569.46±625.87 | 4.168 | 0.022 | 0.284 |
| lh-parsorbitalis | 1872.40±269.46 | 1951.11±267.20 | 1839.50±231.83 | 2.490 | 0.094 | 0.503 |
| lh-parstriangularis | 3013.45±634.55 | 3098.33±332.10 | 2829.75±517.01 | 1.695 | 0.195 | 0.538 |
| lh-pericalcarine | 1929.40±474.18 | 1736.11±431.04 | 2033.63±384.09 | 1.227 | 0.303 | 0.567 |
| lh-postcentral | 8171.10±993.67 | 9062.22±1107.08 | 8476.63±1071.67 | 1.394 | 0.259 | 0.567 |
| lh-posteriorcingulate | 2574.40±612.92 | 2714.56±508.04 | 2564.79±548.84 | 3.000 | 0.060 | 0.454 |
| lh-precentral | 11654.65±1498.83 | 12619.67±1929.41 | 11819.75±1399.70 | 0.897 | 0.415 | 0.649 |
| lh-precuneus | 7216.35±1294.16 | 8053.56±894.09 | 7646.63±1058.60 | 4.492 | 0.017 | 0.252 |
| lh-rostralanteriorcingulate | 2125.75±528.09 | 2190.11±378.95 | 2025.67±426.54 | 0.320 | 0.727 | 0.855 |
| lh-rostralmiddlefrontal | 12029.10±1875.76 | 12554.56±1290.57 | 11328.00±1687.37 | 0.272 | 0.763 | 0.855 |
| lh-superiorfrontal | 18354.60±2056.44 | 19050.56±1767.50 | 18313.17±2619.30 | 0.259 | 0.773 | 0.855 |
| lh-superiorparietal | 10593.85±1418.34 | 11421.11±1602.80 | 10513.71±1781.89 | 1.081 | 0.348 | 0.576 |
| lh-superiortemporal | 9823.75±1556.94 | 11028.00±1242.22 | 9593.29±1668.84 | 0.249 | 0.780 | 0.855 |
| lh-supramarginal | 8718.35±1700.43 | 9474.00±1132.98 | 8458.92±1736.63 | 2.596 | 0.086 | 0.503 |
| lh-temporalpole | 2260.20±428.53 | 2446.22±366.22 | 2231.29±397.00 | 1.213 | 0.307 | 0.567 |
| lh-transversetemporal | 999.50±208.59 | 1111.33±188.43 | 982.83±183.59 | 0.124 | 0.884 | 0.893 |
| rh-bankssts | 1558.90±293.27 | 1641.44±249.36 | 1509.17±364.94 | 0.267 | 0.767 | 0.855 |
| rh-caudalanteriorcingulate | 1718.40±474.65 | 1699.89±361.79 | 1682.71±352.02 | 0.435 | 0.650 | 0.833 |
| rh-caudalmiddlefrontal | 4861.65±1071.82 | 4630.89±844.31 | 4503.13±955.81 | 1.192 | 0.313 | 0.567 |
| rh-cuneus | 2930.85±314.99 | 2977.89±448.21 | 3121.04±530.63 | 1.028 | 0.366 | 0.585 |
| rh-entorhinal | 1469.70±352.19 | 1370.67±496.78 | 1333.08±479.38 | 0.217 | 0.806 | 0.863 |
| rh-frontalpole | 1010.50±218.96 | 1112.22±61.47 | 996.79±151.91 | 1.821 | 0.174 | 0.537 |
| rh-fusiform | 7760.25±1376.77 | 8219.33±1232.36 | 7319.96±1301.26 | 0.704 | 0.500 | 0.711 |
| rh-inferiorparietal | 10958.35±1668.50 | 12370.44±1640.07 | 10595.38±1932.55 | 1.551 | 0.223 | 0.560 |
| rh-inferiortemporal | 8207.10±1272.60 | 8885.00±1235.07 | 7844.13±1381.85 | 0.154 | 0.858 | 0.892 |
| rh-insula | 5865.00±678.94 | 6102.67±675.05 | 5823.79±820.74 | 0.333 | 0.719 | 0.855 |
| rh-isthmuscingulate | 2163.75±377.16 | 1927.22±301.50 | 2095.42±355.54 | 0.410 | 0.666 | 0.842 |
| rh-lateraloccipital | 9775.70±1325.79 | 10590.56±1257.39 | 9636.25±1527.60 | 4.786 | 0.013 | 0.237 |
| rh-lateralorbitofrontal | 6429.70±707.69 | 6315.22±838.44 | 6138.25±773.65 | 1.731 | 0.189 | 0.537 |
| rh-lingual | 5871.15±632.82 | 5880.56±724.69 | 6389.00±981.16 | 3.077 | 0.056 | 0.454 |
| rh-medialorbitofrontal | 4811.20±625.71 | 4891.89±610.93 | 4611.96±564.49 | 0.265 | 0.768 | 0.855 |
| rh-middletemporal | 9399.45±1702.66 | 10119.56±1359.14 | 8528.33±1746.43 | 0.660 | 0.522 | 0.731 |
| rh-paracentral | 3513.80±488.09 | 3451.11±423.89 | 3594.42±390.68 | 1.514 | 0.231 | 0.560 |
| rh-parahippocampal | 1558.90±226.36 | 1462.44±204.22 | 1539.38±285.43 | 1.126 | 0.333 | 0.572 |
| rh-parsopercularis | 3183.50±640.10 | 3282.00±497.69 | 3120.71±599.22 | 3.125 | 0.054 | 0.454 |
| rh-parsorbitalis | 2373.60±250.96 | 2458.11±295.82 | 2287.00±387.55 | 2.731 | 0.076 | 0.503 |
| rh-parstriangularis | 3460.70±510.76 | 3557.78±594.88 | 3398.54±598.64 | 1.501 | 0.234 | 0.560 |
| rh-pericalcarine | 2218.10±428.98 | 2108.44±385.31 | 2386.54±419.65 | 2.304 | 0.112 | 0.534 |
| rh-postcentral | 7911.30±1318.61 | 8616.33±1059.76 | 8111.63±1002.12 | 2.430 | 0.100 | 0.503 |
| rh-posteriorcingulate | 2547.35±508.40 | 2543.56±386.95 | 2510.46±557.15 | 0.294 | 0.747 | 0.855 |
| rh-precentral | 11425.85±1573.63 | 12347.78±2012.33 | 11518.63±1278.70 | 0.590 | 0.558 | 0.758 |
| rh-precuneus | 8120.65±1218.85 | 8493.67±1118.80 | 8295.08±1228.38 | 1.914 | 0.159 | 0.537 |
| rh-rostralanteriorcingulate | 1656.10±449.72 | 1677.33±465.61 | 1584.58±299.13 | 1.372 | 0.264 | 0.567 |
| rh-rostralmiddlefrontal | 12610.90±1962.55 | 12281.67±1149.23 | 11855.13±1916.25 | 0.316 | 0.731 | 0.855 |
| rh-superiorfrontal | 18120.00±2241.81 | 18265.44±1681.69 | 17292.42±2415.70 | 1.590 | 0.215 | 0.559 |
| rh-superiorparietal | 10103.15±1421.47 | 10386.67±1302.09 | 10166.13±1455.07 | 1.626 | 0.208 | 0.557 |
| rh-superiortemporal | 9188.95±1317.57 | 10208.33±1193.90 | 9256.00±1352.28 | 1.332 | 0.274 | 0.567 |
| rh-supramarginal | 7848.05±1547.28 | 7804.89±466.34 | 7496.54±1459.13 | 1.742 | 0.187 | 0.537 |
| rh-temporalpole | 2431.40±412.68 | 2399.44±495.86 | 2331.50±396.01 | 2.430 | 0.099 | 0.503 |
| rh-transversetemporal | 749.55±195.73 | 790.56±135.05 | 745.08±141.80 | 1.811 | 0.175 | 0.537 |

**Table S10. Comparison of AV45-PET SUVR value among ApoE ε4 Homozygous, Heterozygous, and Non-Carrier in AD group**

| **Brain region** | **Heterozygous** | **Homozygous** | **Non-carrier** | **F** | **P** | **FDR-P** |
| --- | --- | --- | --- | --- | --- | --- |
| Left-Amygdala | 1.04±0.12 | 1.06±0.11 | 1.07±0.15 | 0.657 | 0.523 | 0.680 |
| Left-Caudate | 1.04±0.16 | 1.05±0.18 | 1.09±0.18 | 0.516 | 0.600 | 0.701 |
| Left-Hippocampus | 0.95±0.07 | 0.94±0.17 | 0.91±0.13 | 0.656 | 0.524 | 0.680 |
| Left-Pallidum | 1.36±0.19 | 1.31±0.12 | 1.39±0.19 | 0.887 | 0.419 | 0.638 |
| Left-Putamen | 1.31±0.16 | 1.25±0.13 | 1.38±0.18 | 2.844 | 0.068 | 0.379 |
| Left-Thalamus | 1.24±0.10 | 1.19±0.16 | 1.22±0.12 | 1.232 | 0.301 | 0.591 |
| Right-Amygdala | 1.09±0.11 | 1.14±0.14 | 1.13±0.14 | 0.711 | 0.496 | 0.680 |
| Right-Caudate | 1.10±0.15 | 1.12±0.13 | 1.17±0.20 | 0.870 | 0.426 | 0.638 |
| Right-Hippocampus | 0.96±0.09 | 0.93±0.09 | 0.93±0.13 | 0.611 | 0.547 | 0.680 |
| Right-Pallidum | 1.36±0.16 | 1.35±0.10 | 1.42±0.19 | 0.652 | 0.526 | 0.680 |
| Right-Putamen | 1.35±0.15 | 1.29±0.10 | 1.45±0.19 | 3.446 | 0.040 | 0.379 |
| Right-Thalamus | 1.28±0.10 | 1.23±0.16 | 1.27±0.15 | 0.943 | 0.397 | 0.638 |
| lh-bankssts | 1.60±0.28 | 1.62±0.21 | 1.66±0.24 | 0.435 | 0.650 | 0.712 |
| lh-caudalanteriorcingulate | 1.36±0.19 | 1.38±0.14 | 1.38±0.25 | 0.183 | 0.834 | 0.853 |
| lh-caudalmiddlefrontal | 1.44±0.23 | 1.54±0.18 | 1.58±0.28 | 1.747 | 0.186 | 0.408 |
| lh-cuneus | 1.13±0.16 | 1.20±0.39 | 1.29±0.26 | 2.701 | 0.078 | 0.379 |
| lh-entorhinal | 0.79±0.10 | 0.81±0.12 | 0.83±0.12 | 1.044 | 0.360 | 0.627 |
| lh-frontalpole | 1.56±0.35 | 1.48±0.20 | 1.53±0.29 | 0.070 | 0.933 | 0.933 |
| lh-fusiform | 1.26±0.10 | 1.36±0.21 | 1.37±0.24 | 2.362 | 0.106 | 0.379 |
| lh-inferiorparietal | 1.43±0.17 | 1.54±0.25 | 1.56±0.24 | 2.242 | 0.118 | 0.379 |
| lh-inferiortemporal | 1.34±0.14 | 1.42±0.18 | 1.43±0.24 | 2.123 | 0.131 | 0.379 |
| lh-insula | 1.17±0.16 | 1.20±0.11 | 1.25±0.19 | 1.108 | 0.339 | 0.614 |
| lh-isthmuscingulate | 1.29±0.21 | 1.35±0.16 | 1.39±0.26 | 0.857 | 0.431 | 0.638 |
| lh-lateraloccipital | 1.33±0.17 | 1.49±0.35 | 1.48±0.28 | 2.723 | 0.076 | 0.379 |
| lh-lateralorbitofrontal | 1.38±0.20 | 1.37±0.16 | 1.44±0.20 | 0.427 | 0.655 | 0.712 |
| lh-lingual | 1.15±0.17 | 1.22±0.31 | 1.28±0.27 | 1.816 | 0.174 | 0.408 |
| lh-medialorbitofrontal | 1.34±0.23 | 1.38±0.14 | 1.40±0.23 | 0.452 | 0.639 | 0.712 |
| lh-middletemporal | 1.34±0.22 | 1.36±0.21 | 1.38±0.25 | 0.491 | 0.615 | 0.704 |
| lh-paracentral | 1.28±0.18 | 1.38±0.17 | 1.41±0.21 | 2.891 | 0.066 | 0.379 |
| lh-parahippocampal | 0.98±0.17 | 1.05±0.09 | 1.07±0.22 | 1.863 | 0.167 | 0.408 |
| lh-parsopercularis | 1.37±0.17 | 1.39±0.12 | 1.43±0.23 | 0.571 | 0.569 | 0.688 |
| lh-parsorbitalis | 1.37±0.23 | 1.38±0.17 | 1.45±0.25 | 0.510 | 0.604 | 0.701 |
| lh-parstriangularis | 1.40±0.21 | 1.39±0.12 | 1.47±0.24 | 0.432 | 0.652 | 0.712 |
| lh-pericalcarine | 1.35±0.25 | 1.44±0.43 | 1.57±0.35 | 2.579 | 0.087 | 0.379 |
| lh-postcentral | 1.27±0.16 | 1.34±0.19 | 1.39±0.23 | 2.363 | 0.105 | 0.379 |
| lh-posteriorcingulate | 1.34±0.22 | 1.45±0.21 | 1.49±0.21 | 2.722 | 0.076 | 0.379 |
| lh-precentral | 1.29±0.16 | 1.34±0.16 | 1.40±0.21 | 2.047 | 0.141 | 0.379 |
| lh-precuneus | 1.44±0.21 | 1.53±0.21 | 1.59±0.27 | 2.574 | 0.087 | 0.379 |
| lh-rostralanteriorcingulate | 1.35±0.25 | 1.34±0.17 | 1.39±0.26 | 0.148 | 0.863 | 0.873 |
| lh-rostralmiddlefrontal | 1.59±0.27 | 1.60±0.15 | 1.64±0.27 | 0.246 | 0.783 | 0.811 |
| lh-superiorfrontal | 1.40±0.22 | 1.48±0.15 | 1.49±0.25 | 1.044 | 0.360 | 0.627 |
| lh-superiorparietal | 1.35±0.14 | 1.49±0.24 | 1.53±0.22 | 4.253 | 0.020 | 0.379 |
| lh-superiortemporal | 1.28±0.18 | 1.26±0.14 | 1.32±0.21 | 0.358 | 0.701 | 0.753 |
| lh-supramarginal | 1.41±0.16 | 1.41±0.13 | 1.48±0.22 | 0.734 | 0.486 | 0.680 |
| lh-temporalpole | 1.01±0.12 | 1.08±0.17 | 1.11±0.19 | 2.305 | 0.111 | 0.379 |
| lh-transversetemporal | 1.23±0.19 | 1.22±0.09 | 1.31±0.20 | 1.283 | 0.287 | 0.581 |
| rh-bankssts | 1.61±0.22 | 1.66±0.21 | 1.70±0.29 | 0.876 | 0.423 | 0.638 |
| rh-caudalanteriorcingulate | 1.41±0.21 | 1.49±0.12 | 1.51±0.25 | 1.217 | 0.306 | 0.591 |
| rh-caudalmiddlefrontal | 1.44±0.21 | 1.47±0.20 | 1.55±0.26 | 1.584 | 0.216 | 0.448 |
| rh-cuneus | 1.17±0.17 | 1.24±0.48 | 1.33±0.26 | 2.174 | 0.125 | 0.379 |
| rh-entorh-inal | 0.83±0.11 | 0.86±0.11 | 0.88±0.25 | 0.874 | 0.424 | 0.638 |
| rh-frontalpole | 1.42±0.25 | 1.49±0.20 | 1.55±0.29 | 1.752 | 0.185 | 0.408 |
| rh-fusiform | 1.28±0.11 | 1.35±0.23 | 1.42±0.21 | 4.044 | 0.024 | 0.379 |
| rh-inferiorparietal | 1.46±0.19 | 1.53±0.20 | 1.59±0.23 | 2.285 | 0.113 | 0.379 |
| rh-inferiortemporal | 1.36±0.15 | 1.48±0.26 | 1.50±0.24 | 3.770 | 0.030 | 0.379 |
| rh-insula | 1.15±0.16 | 1.17±0.16 | 1.24±0.17 | 1.737 | 0.187 | 0.408 |
| rh-isthmuscingulate | 1.34±0.23 | 1.48±0.16 | 1.50±0.23 | 3.133 | 0.053 | 0.379 |
| rh-lateraloccipital | 1.40±0.17 | 1.51±0.47 | 1.53±0.27 | 2.149 | 0.128 | 0.379 |
| rh-lateralorbitofrontal | 1.37±0.18 | 1.39±0.14 | 1.47±0.20 | 1.755 | 0.184 | 0.408 |
| rh-lingual | 1.16±0.16 | 1.25±0.44 | 1.32±0.22 | 2.949 | 0.062 | 0.379 |
| rh-medialorbitofrontal | 1.35±0.22 | 1.39±0.12 | 1.42±0.26 | 0.739 | 0.483 | 0.680 |
| rh-middletemporal | 1.41±0.15 | 1.47±0.23 | 1.49±0.24 | 1.764 | 0.183 | 0.408 |
| rh-paracentral | 1.36±0.17 | 1.42±0.22 | 1.50±0.20 | 3.552 | 0.037 | 0.379 |
| rh-parahippocampal | 1.06±0.12 | 1.14±0.16 | 1.18±0.22 | 3.356 | 0.044 | 0.379 |
| rh-parsopercularis | 1.37±0.19 | 1.36±0.20 | 1.46±0.22 | 1.163 | 0.322 | 0.601 |
| rh-parsorbitalis | 1.36±0.22 | 1.35±0.21 | 1.42±0.28 | 0.282 | 0.756 | 0.792 |
| rh-parstriangularis | 1.41±0.18 | 1.39±0.19 | 1.50±0.26 | 0.978 | 0.384 | 0.638 |
| rh-pericalcarine | 1.42±0.26 | 1.48±0.43 | 1.58±0.31 | 1.633 | 0.206 | 0.438 |
| rh-postcentral | 1.29±0.15 | 1.34±0.23 | 1.41±0.19 | 2.516 | 0.092 | 0.379 |
| rh-posteriorcingulate | 1.42±0.21 | 1.57±0.15 | 1.59±0.25 | 3.729 | 0.032 | 0.379 |
| rh-precentral | 1.28±0.13 | 1.31±0.20 | 1.40±0.19 | 2.674 | 0.080 | 0.379 |
| rh-precuneus | 1.45±0.20 | 1.57±0.21 | 1.62±0.24 | 3.555 | 0.037 | 0.379 |
| rh-rostralanteriorcingulate | 1.38±0.21 | 1.43±0.14 | 1.48±0.31 | 0.853 | 0.433 | 0.638 |
| rh-rostralmiddlefrontal | 1.54±0.22 | 1.60±0.19 | 1.63±0.27 | 0.671 | 0.516 | 0.680 |
| rh-superiorfrontal | 1.39±0.20 | 1.48±0.16 | 1.52±0.25 | 2.057 | 0.139 | 0.379 |
| rh-superiorparietal | 1.38±0.17 | 1.47±0.27 | 1.52±0.23 | 2.787 | 0.072 | 0.379 |
| rh-superiortemporal | 1.29±0.17 | 1.27±0.15 | 1.35±0.24 | 0.622 | 0.541 | 0.680 |
| rh-supramarginal | 1.40±0.17 | 1.42±0.20 | 1.51±0.21 | 2.024 | 0.144 | 0.379 |
| rh-temporalpole | 1.01±0.13 | 1.06±0.23 | 1.14±0.25 | 2.903 | 0.065 | 0.379 |
| rh-transversetemporal | 1.10±0.22 | 1.16±0.17 | 1.22±0.23 | 2.052 | 0.140 | 0.379 |

**Table S11. Comparison of ^18^F-FDG PET SUVR Values among ApoE ε4 Homozygous, Heterozygous, and Non-Carrier in AD group**

| **Brain region** | **Heterozygous** | **Homozygous** | **Non-carrier** | **F** | **P** | **FDR-P** |
| --- | --- | --- | --- | --- | --- | --- |
| Left-Amygdala | 0.9677 ± 0.0900 | 0.9612 ± 0.1126 | 0.9827 ± 0.1254 | 0.857 | 0.431 | 0.997 |
| Left-Caudate | 1.4439 ± 0.1890 | 1.4543 ± 0.3692 | 1.4840 ± 0.3171 | 1.202 | 0.310 | 0.997 |
| Left-Hippocampus | 0.9921 ± 0.0960 | 0.9253 ± 0.1271 | 1.0348 ± 0.1467 | 1.382 | 0.261 | 0.997 |
| Left-Pallidum | 1.2130 ± 0.0918 | 1.2015 ± 0.1475 | 1.2726 ± 0.2310 | 1.387 | 0.260 | 0.997 |
| Left-Putamen | 1.9403 ± 0.1955 | 1.8701 ± 0.3871 | 2.0875 ± 0.5203 | 0.097 | 0.908 | 0.997 |
| Left-Thalamus | 1.4526 ± 0.1392 | 1.4136 ± 0.2285 | 1.5051 ± 0.2127 | 1.722 | 0.190 | 0.997 |
| Right-Amygdala | 1.0260 ± 0.1290 | 1.0111 ± 0.1487 | 1.0369 ± 0.1255 | 1.157 | 0.323 | 0.997 |
| Right-Caudate | 1.4770 ± 0.1981 | 1.4923 ± 0.3705 | 1.5541 ± 0.3050 | 0.673 | 0.515 | 0.997 |
| Right-Hippocampus | 1.0264 ± 0.1009 | 0.9618 ± 0.1348 | 1.0537 ± 0.1317 | 1.312 | 0.279 | 0.997 |
| Right-Pallidum | 1.2960 ± 0.1329 | 1.2858 ± 0.1707 | 1.3694 ± 0.2236 | 0.627 | 0.539 | 0.997 |
| Right-Putamen | 1.9664 ± 0.1871 | 1.8720 ± 0.4021 | 2.1389 ± 0.4608 | 0.140 | 0.870 | 0.997 |
| Right-Thalamus | 1.4680 ± 0.1204 | 1.4312 ± 0.2531 | 1.5253 ± 0.1841 | 3.139 | 0.053 | 0.997 |
| lh-bankssts | 1.5093 ± 0.3558 | 1.7254 ± 0.3762 | 1.5417 ± 0.4515 | 1.815 | 0.174 | 0.997 |
| lh-caudalanteriorcingulate | 1.3722 ± 0.1804 | 1.3473 ± 0.2260 | 1.4098 ± 0.2006 | 0.199 | 0.820 | 0.997 |
| lh-caudalmiddlefrontal | 1.8048 ± 0.2860 | 1.8506 ± 0.4590 | 1.8283 ± 0.4459 | 0.316 | 0.731 | 0.997 |
| lh-cuneus | 1.8959 ± 0.3467 | 1.9002 ± 0.5443 | 1.9646 ± 0.4186 | 0.282 | 0.756 | 0.997 |
| lh-entorhinal | 0.8978 ± 0.1414 | 0.8673 ± 0.1866 | 0.9086 ± 0.1726 | 0.049 | 0.952 | 0.997 |
| lh-frontalpole | 1.8791 ± 0.2854 | 1.7470 ± 0.4099 | 1.7745 ± 0.3539 | 0.994 | 0.378 | 0.997 |
| lh-fusiform | 1.4514 ± 0.1961 | 1.5530 ± 0.3865 | 1.5121 ± 0.3401 | 0.845 | 0.436 | 0.997 |
| lh-inferiorparietal | 1.5782 ± 0.3809 | 1.6638 ± 0.4136 | 1.5729 ± 0.4449 | 1.696 | 0.195 | 0.997 |
| lh-inferiortemporal | 1.3538 ± 0.2944 | 1.5285 ± 0.3670 | 1.3838 ± 0.3597 | 0.124 | 0.883 | 0.997 |
| lh-insula | 1.2789 ± 0.1583 | 1.3502 ± 0.3670 | 1.4081 ± 0.3370 | 0.248 | 0.781 | 0.997 |
| lh-isthmuscingulate | 1.4894 ± 0.2827 | 1.4020 ± 0.3009 | 1.5130 ± 0.2784 | 0.068 | 0.934 | 0.997 |
| lh-lateraloccipital | 1.7939 ± 0.3268 | 1.8136 ± 0.5389 | 1.7758 ± 0.4952 | 0.613 | 0.546 | 0.997 |
| lh-lateralorbitofrontal | 1.6912 ± 0.2121 | 1.5924 ± 0.3303 | 1.6728 ± 0.2900 | 0.478 | 0.623 | 0.997 |
| lh-lingual | 1.7219 ± 0.3016 | 1.7804 ± 0.4797 | 1.8378 ± 0.3910 | 1.282 | 0.287 | 0.997 |
| lh-medialorbitofrontal | 1.4891 ± 0.1638 | 1.5000 ± 0.3382 | 1.5485 ± 0.2065 | 0.477 | 0.624 | 0.997 |
| lh-middletemporal | 1.4380 ± 0.3009 | 1.5302 ± 0.3676 | 1.4601 ± 0.4244 | 0.384 | 0.683 | 0.997 |
| lh-paracentral | 1.6796 ± 0.3046 | 1.6181 ± 0.3747 | 1.7221 ± 0.2886 | 0.410 | 0.666 | 0.997 |
| lh-parahippocampal | 1.1254 ± 0.1519 | 1.1455 ± 0.2624 | 1.1731 ± 0.2404 | 0.003 | 0.997 | 0.997 |
| lh-parsopercularis | 1.7471 ± 0.2555 | 1.7789 ± 0.4392 | 1.7934 ± 0.3850 | 0.517 | 0.600 | 0.997 |
| lh-parsorbitalis | 1.7636 ± 0.4938 | 1.7008 ± 0.4870 | 1.7365 ± 0.4477 | 0.007 | 0.993 | 0.997 |
| lh-parstriangularis | 1.7911 ± 0.2611 | 1.8550 ± 0.5133 | 1.8391 ± 0.3564 | 0.572 | 0.568 | 0.997 |
| lh-pericalcarine | 2.1724 ± 0.4439 | 2.1282 ± 0.6220 | 2.1740 ± 0.5092 | 0.923 | 0.405 | 0.997 |
| lh-postcentral | 1.6963 ± 0.2679 | 1.7260 ± 0.4515 | 1.7989 ± 0.3459 | 0.622 | 0.542 | 0.997 |
| lh-posteriorcingulate | 1.6006 ± 0.2544 | 1.6585 ± 0.4132 | 1.7409 ± 0.3782 | 0.154 | 0.858 | 0.997 |
| lh-precentral | 1.7510 ± 0.2548 | 1.7549 ± 0.4175 | 1.8512 ± 0.3377 | 1.350 | 0.269 | 0.997 |
| lh-precuneus | 1.7169 ± 0.3357 | 1.6872 ± 0.3916 | 1.7603 ± 0.4412 | 0.028 | 0.973 | 0.997 |
| lh-rostralanteriorcingulate | 1.3321 ± 0.1613 | 1.3567 ± 0.3287 | 1.4428 ± 0.2248 | 0.026 | 0.974 | 0.997 |
| lh-rostralmiddlefrontal | 1.8483 ± 0.3199 | 1.7973 ± 0.4316 | 1.8578 ± 0.4349 | 0.932 | 0.401 | 0.997 |
| lh-superiorfrontal | 1.6958 ± 0.2309 | 1.6353 ± 0.4197 | 1.6902 ± 0.3531 | 1.605 | 0.212 | 0.997 |
| lh-superiorparietal | 1.6185 ± 0.2349 | 1.7126 ± 0.4706 | 1.7129 ± 0.4192 | 0.604 | 0.551 | 0.997 |
| lh-superiortemporal | 1.3985 ± 0.2393 | 1.4764 ± 0.3529 | 1.4900 ± 0.3341 | 0.420 | 0.659 | 0.997 |
| lh-supramarginal | 1.5463 ± 0.3335 | 1.6092 ± 0.3700 | 1.5767 ± 0.4033 | 2.019 | 0.144 | 0.997 |
| lh-temporalpole | 1.0644 ± 0.1475 | 1.2198 ± 0.3585 | 1.1722 ± 0.2629 | 0.208 | 0.813 | 0.997 |
| lh-transversetemporal | 1.9256 ± 0.4399 | 1.9236 ± 0.6148 | 1.9781 ± 0.5698 | 1.092 | 0.344 | 0.997 |
| rh-bankssts | 1.5468 ± 0.3424 | 1.6699 ± 0.4440 | 1.5489 ± 0.3340 | 1.775 | 0.181 | 0.997 |
| rh-caudalanteriorcingulate | 1.4284 ± 0.1534 | 1.4203 ± 0.2929 | 1.4954 ± 0.1647 | 0.710 | 0.497 | 0.997 |
| rh-caudalmiddlefrontal | 1.7908 ± 0.3099 | 1.8398 ± 0.5182 | 1.8354 ± 0.4030 | 0.348 | 0.708 | 0.997 |
| rh-cuneus | 1.8804 ± 0.3085 | 1.8007 ± 0.5151 | 1.9841 ± 0.3962 | 0.426 | 0.655 | 0.997 |
| rh-entorhinal | 0.9398 ± 0.1647 | 0.8986 ± 0.1799 | 0.9377 ± 0.1884 | 0.015 | 0.985 | 0.997 |
| rh-frontalpole | 1.8218 ± 0.3616 | 1.7618 ± 0.4440 | 1.7828 ± 0.3232 | 1.266 | 0.292 | 0.997 |
| rh-fusiform | 1.4575 ± 0.1624 | 1.5396 ± 0.3980 | 1.5492 ± 0.2894 | 0.772 | 0.468 | 0.997 |
| rh-inferiorparietal | 1.5410 ± 0.3726 | 1.5909 ± 0.4356 | 1.5584 ± 0.3471 | 1.435 | 0.248 | 0.997 |
| rh-inferiortemporal | 1.4024 ± 0.2510 | 1.5211 ± 0.3844 | 1.4125 ± 0.2875 | 0.156 | 0.856 | 0.997 |
| rh-insula | 1.2717 ± 0.2091 | 1.3000 ± 0.3125 | 1.4181 ± 0.3457 | 0.406 | 0.669 | 0.997 |
| rh-isthmuscingulate | 1.5679 ± 0.2232 | 1.4817 ± 0.2929 | 1.5807 ± 0.2060 | 0.046 | 0.955 | 0.997 |
| rh-lateraloccipital | 1.7595 ± 0.3087 | 1.7789 ± 0.5065 | 1.7797 ± 0.4076 | 1.073 | 0.350 | 0.997 |
| rh-lateralorbitofrontal | 1.6440 ± 0.2249 | 1.5964 ± 0.3332 | 1.6569 ± 0.2170 | 0.430 | 0.653 | 0.997 |
| rh-lingual | 1.7375 ± 0.2748 | 1.7366 ± 0.4497 | 1.8788 ± 0.3338 | 1.259 | 0.293 | 0.997 |
| rh-medialorbitofrontal | 1.5212 ± 0.1929 | 1.5099 ± 0.3219 | 1.5735 ± 0.1784 | 0.937 | 0.399 | 0.997 |
| rh-middletemporal | 1.4794 ± 0.3330 | 1.5438 ± 0.3741 | 1.4531 ± 0.3832 | 0.235 | 0.792 | 0.997 |
| rh-paracentral | 1.7144 ± 0.2925 | 1.7097 ± 0.3987 | 1.7607 ± 0.2615 | 0.271 | 0.764 | 0.997 |
| rh-parahippocampal | 1.1849 ± 0.1508 | 1.2463 ± 0.2903 | 1.2283 ± 0.1927 | 0.236 | 0.791 | 0.997 |
| rh-parsopercularis | 1.7219 ± 0.2746 | 1.7171 ± 0.4369 | 1.7698 ± 0.3142 | 0.196 | 0.823 | 0.997 |
| rh-parsorbitalis | 1.7768 ± 0.3398 | 1.6644 ± 0.4593 | 1.6652 ± 0.4196 | 0.094 | 0.911 | 0.997 |
| rh-parstriangularis | 1.8256 ± 0.2741 | 1.8087 ± 0.4676 | 1.8473 ± 0.3430 | 0.799 | 0.456 | 0.997 |
| rh-pericalcarine | 2.1161 ± 0.4097 | 2.0311 ± 0.5609 | 2.1607 ± 0.4715 | 0.585 | 0.561 | 0.997 |
| rh-postcentral | 1.6902 ± 0.2636 | 1.7448 ± 0.4748 | 1.8002 ± 0.2952 | 0.619 | 0.543 | 0.997 |
| rh-posteriorcingulate | 1.6817 ± 0.2662 | 1.6573 ± 0.3809 | 1.7925 ± 0.3732 | 0.144 | 0.866 | 0.997 |
| rh-precentral | 1.7630 ± 0.2282 | 1.7663 ± 0.4698 | 1.8589 ± 0.3230 | 1.267 | 0.291 | 0.997 |
| rh-precuneus | 1.7206 ± 0.2967 | 1.6605 ± 0.3710 | 1.7504 ± 0.2912 | 0.022 | 0.979 | 0.997 |
| rh-rostralanteriorcingulate | 1.3865 ± 0.1874 | 1.4101 ± 0.3522 | 1.5136 ± 0.2607 | 0.061 | 0.941 | 0.997 |
| rh-rostralmiddlefrontal | 1.8098 ± 0.3343 | 1.7598 ± 0.4224 | 1.8141 ± 0.3560 | 2.034 | 0.142 | 0.997 |
| rh-superiorfrontal | 1.7201 ± 0.2147 | 1.6862 ± 0.4474 | 1.7259 ± 0.3281 | 1.482 | 0.238 | 0.997 |
| rh-superiorparietal | 1.5824 ± 0.2608 | 1.7075 ± 0.5082 | 1.7158 ± 0.3363 | 0.799 | 0.456 | 0.997 |
| rh-superiortemporal | 1.4166 ± 0.2000 | 1.4620 ± 0.3630 | 1.5037 ± 0.2568 | 0.115 | 0.892 | 0.997 |
| rh-supramarginal | 1.5227 ± 0.2783 | 1.5693 ± 0.4391 | 1.5875 ± 0.3055 | 0.741 | 0.482 | 0.997 |
| rh-temporalpole | 1.0954 ± 0.2097 | 1.1631 ± 0.3374 | 1.1697 ± 0.2411 | 0.198 | 0.821 | 0.997 |
| rh-transversetemporal | 1.8262 ± 0.4252 | 1.8333 ± 0.6011 | 1.8800 ± 0.5374 | 1.183 | 0.315 | 0.997 |

**Table S12.** Comparison of clinical features and cognitive scale scores between genotyped and non-genotyped subgroups

| **Indicators** | Genotyped group **(n=71)** | genotyped group **(n=63)** | **P value** |
| --- | --- | --- | --- |
| Age (years, x±s) | 69.63±7.11 | 69.17±7.88 | 0.723 |
| Male [n (%)] | 26（36.62%） | 28（44.44%） | 0.456 |
| Disease duration (years) | 3.0（2.0,6.0） | 3.0（2.0,5.0） | 0.931 |
| Educational years (years) | 12.0（9.0,15.0） | 9.0（9.0,15.0） | 0.496 |
| MMSE (points) | 18.0（13.0,23.0） | 17.0（11.0,23.0） | 0.492 |
| MoCA (points, x±s) | 13.18±6.13 | 12.32±5.99 | 0.466 |
| HAMA (points) | 10.0（5.0,16.0） | 10.0（6.0,13.0） | 0.835 |
| HAMD (points) | 9.0（6.0,14.0） | 10.0（7.0,13.0） | 0.387 |
| ADL (points) | 24.0（18.0,34.0） | 24.0（18.0,31.0） | 0.911 |
|  |  |  |  |

**Table S13.** Comparison of regional **Gray Matter Volume** between genotyped and non-genotyped subgroups

| **Brain region** | **Genotyped group (n=71)** | **genotyped group (n=63)** | **F** | **P** | **FDR-P** |
| --- | --- | --- | --- | --- | --- |
| lh-bankssts | 1741.23±395.63 | 1854.10±382.29 | 3.675 | .096 | 0.950 |
| lh-caudalanteriorcingulate | 1369.51±419.25 | 1493.14±411.32 | 2.719 | .088 | 0.950 |
| lh-caudalmiddlefrontal | 4885.56±955.92 | 4716.79±919.41 | 1.038 | .301 | 0.950 |
| lh-cuneus | 2711.63±472.33 | 2690.00±402.85 | 0.283 | .777 | 0.950 |
| lh-entorhinal | 1389.54±441.30 | 1489.11±431.07 | 1.318 | .190 | 0.950 |
| lh-fusiform | 8035.72±1586.76 | 8199.43±1361.18 | 0.637 | .525 | 0.950 |
| lh-inferiorparietal | 9786.21±1714.82 | 10120.40±1882.16 | 1.075 | .284 | 0.950 |
| lh-inferiortemporal | 8893.63±1742.96 | 8917.03±1823.71 | 4.076 | .940 | 0.950 |
| lh-isthmuscingulate | 2325.21±498.45 | 2339.92±442.38 | 2.180 | .858 | 0.950 |
| lh-lateraloccipital | 10435.55±1671.98 | 10416.37±1716.84 | 1.065 | .948 | 0.950 |
| lh-lateralorbitofrontal | 6401.17±855.67 | 6419.79±750.40 | 1.133 | .894 | 0.950 |
| lh-lingual | 5625.08±956.24 | 5697.06±901.07 | 1.447 | .656 | 0.950 |
| lh-medialorbitofrontal | 4590.56±652.47 | 4496.63±671.29 | 1.821 | .413 | 0.950 |
| lh-middletemporal | 8696.38±1782.87 | 8846.22±1640.16 | 2.504 | .615 | 0.950 |
| lh-parahippocampal | 1648.48±389.84 | 1793.14±340.59 | 2.274 | .025 | 0.950 |
| lh-paracentral | 3221.44±398.29 | 3165.94±496.76 | 0.717 | .475 | 0.950 |
| lh-parsopercularis | 3732.24±662.13 | 3822.02±719.99 | 1.752 | .453 | 0.950 |
| lh-parsorbitalis | 1901.37±260.73 | 1891.59±252.83 | 0.220 | .826 | 0.950 |
| lh-parstriangularis | 2975.92±521.23 | 2949.48±482.81 | 0.303 | .762 | 0.950 |
| lh-pericalcarine | 1920.62±436.37 | 1848.86±394.84 | 0.993 | .322 | 0.950 |
| lh-postcentral | 8538.07±1090.90 | 8487.48±1091.83 | 0.268 | .789 | 0.950 |
| lh-posteriorcingulate | 2633.30±539.86 | 2606.57±504.98 | 0.295 | .769 | 0.950 |
| lh-precentral | 12071.00±1594.30 | 11930.98±1527.13 | 0.518 | .606 | 0.950 |
| lh-precuneus | 7920.80±1327.29 | 8027.62±1102.99 | -0.503 | .616 | 0.950 |
| lh-rostralanteriorcingulate | 2136.62±521.13 | 2211.65±450.93 | -0.886 | .377 | 0.950 |
| lh-rostralmiddlefrontal | 12120.61±1756.71 | 12029.56±1816.31 | 0.295 | .769 | 0.950 |
| lh-superiorfrontal | 18819.80±2397.35 | 18476.90±2463.60 | 0.816 | .416 | 0.950 |
| lh-superiorparietal | 11057.86±1677.58 | 11096.95±1745.58 | -0.132 | .895 | 0.950 |
| lh-superiortemporal | 10174.42±1666.32 | 10299.13±1605.95 | -0.440 | .661 | 0.950 |
| lh-supramarginal | 8958.13±1618.94 | 9043.16±1708.71 | -0.296 | .768 | 0.950 |
| lh-frontalpole | 920.73±166.96 | 911.03±165.50 | 0.337 | .737 | 0.950 |
| lh-temporalpole | 2231.99±464.89 | 2289.89±506.63 | -0.690 | .491 | 0.950 |
| lh-transversetemporal | 1041.48±190.49 | 1036.48±215.63 | 0.143 | .887 | 0.950 |
| lh-insula | 6118.65±773.37 | 6223.17±807.29 | -0.765 | .446 | 0.950 |
| rh-bankssts | 1604.11±359.61 | 1616.62±292.55 | -0.222 | .825 | 0.950 |
| rh-caudalanteriorcingulate | 1708.42±391.61 | 1720.56±472.34 | -0.162 | .873 | 0.950 |
| rh-caudalmiddlefrontal | 4758.83±975.93 | 4709.16±990.04 | 0.292 | .771 | 0.950 |
| rh-cuneus | 3081.20±469.37 | 3048.22±496.48 | 0.395 | .693 | 0.950 |
| rh-entorhinal | 1423.32±447.45 | 1507.14±389.00 | -1.160 | .248 | 0.950 |
| rh-fusiform | 7891.54±1586.46 | 8178.79±1467.84 | -1.088 | .278 | 0.950 |
| rh-inferiorparietal | 11529.62±2166.51 | 11703.81±2042.64 | -0.479 | .633 | 0.950 |
| rh-inferiortemporal | 8504.15±1651.36 | 8936.41±1788.13 | -1.448 | .150 | 0.950 |
| rh-isthmuscingulate | 2180.03±412.55 | 2142.59±347.19 | 0.570 | .569 | 0.950 |
| rh-lateraloccipital | 10199.68±1678.60 | 10582.05±1680.49 | -1.315 | .191 | 0.950 |
| rh-lateralorbitofrontal | 6372.70±863.75 | 6363.67±867.66 | 0.060 | .952 | 0.950 |
| rh-lingual | 6257.30±923.21 | 6351.29±951.94 | 0.579 | .564 | 0.950 |
| rh-medialorbitofrontal | 4816.38±661.81 | 4736.89±531.11 | 0.770 | .442 | 0.950 |
| rh-middletemporal | 9452.35±1950.89 | 9736.37±1441.24 | 0.965 | .336 | 0.950 |
| rh-parahippocampal | 1577.13±291.04 | 1648.17±295.12 | 1.400 | .164 | 0.950 |
| rh-paracentral | 3593.82±453.74 | 3644.44±574.24 | 0.561 | .576 | 0.950 |
| rh-parsopercularis | 3232.03±601.97 | 3272.44±531.82 | 0.413 | .681 | 0.950 |
| rh-parsorbitalis | 2376.61±360.04 | 2286.16±286.80 | 1.616 | .108 | 0.950 |
| rh-parstriangularis | 3576.48±628.58 | 3616.48±611.73 | 0.373 | .710 | 0.950 |
| rh-pericalcarine | 2276.08±436.54 | 2224.25±436.95 | 0.686 | .494 | 0.950 |
| rh-postcentral | 8246.42±1213.10 | 8270.30±1193.07 | 0.115 | .909 | 0.950 |
| rh-posteriorcingulate | 2585.31±511.59 | 2665.30±562.14 | 0.857 | .393 | 0.950 |
| rh-precentral | 11777.66±1589.44 | 11616.30±1834.38 | 0.541 | .590 | 0.950 |
| rh-precuneus | 8515.15±1313.99 | 8490.67±1302.68 | 0.108 | .914 | 0.950 |
| rh-rostralanteriorcingulate | 1643.73±394.55 | 1607.98±464.22 | 0.477 | .634 | 0.950 |
| rh-rostralmiddlefrontal | 12639.24±2200.95 | 12841.46±2171.77 | 0.535 | .594 | 0.950 |
| rh-superiorfrontal | 18106.79±2613.11 | 18209.37±2375.04 | 0.238 | .812 | 0.950 |
| rh-superiorparietal | 10581.25±1498.17 | 10471.56±1752.64 | 0.387 | .699 | 0.950 |
| rh-superiortemporal | 9691.25±1565.22 | 10010.95±1237.50 | 1.318 | .190 | 0.950 |
| rh-supramarginal | 7882.72±1351.88 | 8217.30±1270.13 | 1.477 | .142 | 0.950 |
| rh-frontalpole | 1048.10±187.47 | 1037.24±200.02 | 0.323 | .747 | 0.950 |
| rh-temporalpole | 2372.18±460.67 | 2425.75±541.25 | 0.613 | .541 | 0.950 |
| rh-transversetemporal | 783.61±180.36 | 787.41±131.65 | 0.141 | .888 | 0.950 |
| rh-insula | 5985.46±814.48 | 6145.92±812.60 | 1.140 | .257 | 0.950 |
| Left-Thalamus | 6160.59±925.46 | 6077.19±797.25 | 0.560 | .576 | 0.950 |
| Left-Caudate | 3083.51±565.79 | 3034.14±700.20 | 0.445 | .657 | 0.950 |
| Left-Putamen | 4187.50±678.79 | 4206.76±744.08 | 0.156 | .876 | 0.950 |
| Left-Pallidum | 1861.75±249.58 | 1873.62±245.45 | 0.277 | .782 | 0.950 |
| Left-Hippocampus | 3095.45±590.06 | 3187.08±606.59 | 0.884 | .378 | 0.950 |
| Left-Amygdala | 1329.83±365.43 | 1375.93±351.94 | 0.743 | .459 | 0.950 |
| Right-Thalamus | 6482.81±938.20 | 6405.29±801.17 | 0.516 | .607 | 0.950 |
| Right-Caudate | 3202.24±515.68 | 3111.85±574.05 | 0.954 | .342 | 0.950 |
| Right-Putamen | 4374.24±740.81 | 4495.94±830.88 | 0.890 | .375 | 0.950 |
| Right-Pallidum | 1778.76±234.70 | 1822.40±266.52 | 1.000 | .319 | 0.950 |
| Right-Hippocampus | 3232.98±625.04 | 3416.76±547.23 | 1.815 | .072 | 0.950 |
| Right-Amygdala | 1413.09±349.67 | 1529.40±339.06 | 1.953 | .053 | 0.950 |

**Table S14.** Comparison of regional FDG Metabolism between genotyped and non-genotyped subgroups

| **Brain region** | **Genotyped group (n=71)** | **genotyped group (n=63)** | **F** | **P** | **FDR-P** |
| --- | --- | --- | --- | --- | --- |
| Left-Thalamus | 1.501±0.184 | 1.517±0.192 | 0.482 | .631 | .820 |
| Left-Caudate | 1.490±0.299 | 1.506±0.306 | 0.294 | .769 | .888 |
| Left-Putamen | 2.028±0.376 | 2.026±0.303 | 0.026 | .979 | .999 |
| Left-Pallidum | 1.247±0.159 | 1.262±0.167 | 0.529 | .598 | .820 |
| Left-Hippocampus | 1.036±0.157 | 1.069±0.169 | 1.168 | .245 | .808 |
| Left-Amygdala | 0.984±0.113 | 0.972±0.137 | 0.572 | .568 | .808 |
| Right-Thalamus | 1.514±0.176 | 1.528±0.180 | 0.435 | .664 | .820 |
| Right-Caudate | 1.545±0.286 | 1.560±0.341 | 0.270 | .787 | .888 |
| Right-Putamen | 2.070±0.365 | 2.078±0.337 | 0.139 | .890 | .950 |
| Right-Pallidum | 1.341±0.171 | 1.376±0.205 | 1.069 | .287 | .808 |
| Right-Hippocampus | 1.073±0.166 | 1.146±0.176 | 2.471 | .015 | .648 |
| Right-Amygdala | 1.046±0.132 | 1.050±0.140 | 0.163 | .871 | .943 |
| lh-bankssts | 1.644±0.409 | 1.780±0.345 | 2.061 | .041 | .648 |
| lh-caudalanteriorcingulate | 1.422±0.208 | 1.459±0.207 | 1.038 | .301 | .808 |
| lh-caudalmiddlefrontal | 1.887±0.409 | 1.928±0.398 | 0.590 | .556 | .808 |
| lh-cuneus | 1.957±0.398 | 1.983±0.335 | 0.404 | .687 | .820 |
| lh-entorhinal | 0.927±0.198 | 0.929±0.215 | 0.063 | .950 | .992 |
| lh-fusiform | 1.544±0.320 | 1.581±0.315 | 0.677 | .500 | .808 |
| lh-inferiorparietal | 1.663±0.438 | 1.712±0.426 | 0.656 | .513 | .808 |
| lh-inferiortemporal | 1.456±0.358 | 1.544±0.325 | 1.488 | .139 | .808 |
| lh-isthmuscingulate | 1.552±0.335 | 1.602±0.345 | 0.838 | .404 | .808 |
| lh-lateraloccipital | 1.812±0.430 | 1.812±0.368 | 0.001 | .999 | .999 |
| lh-lateralorbitofrontal | 1.699±0.302 | 1.736±0.278 | 0.737 | .462 | .808 |
| lh-lingual | 1.810±0.352 | 1.833±0.275 | 0.416 | .678 | .820 |
| lh-medialorbitofrontal | 1.551±0.264 | 1.608±0.293 | 1.185 | .238 | .808 |
| lh-middletemporal | 1.515±0.375 | 1.595±0.304 | 1.342 | .182 | .808 |
| lh-parahippocampal | 1.181±0.241 | 1.209±0.296 | 0.601 | .549 | .808 |
| lh-paracentral | 1.721±0.281 | 1.742±0.237 | 0.476 | .635 | .820 |
| lh-parsopercularis | 1.810±0.364 | 1.858±0.343 | 1.789 | .432 | .808 |
| lh-parsorbitalis | 1.748±0.458 | 1.752±0.409 | 1.056 | .955 | .992 |
| lh-parstriangularis | 1.855±0.364 | 1.906±0.366 | 0.805 | .422 | .808 |
| lh-pericalcarine | 2.201±0.483 | 2.250±0.438 | 0.617 | .538 | .808 |
| lh-postcentral | 1.793±0.325 | 1.862±0.299 | 1.274 | .205 | .808 |
| lh-posteriorcingulate | 1.765±0.391 | 1.860±0.413 | 1.370 | .173 | .808 |
| lh-precentral | 1.822±0.301 | 1.856±0.255 | 0.691 | .491 | .808 |
| lh-precuneus | 1.798±0.409 | 1.831±0.385 | 0.474 | .637 | .820 |
| lh-rostralanteriorcingulate | 1.418±0.263 | 1.473±0.309 | 1.130 | .261 | .808 |
| lh-rostralmiddlefrontal | 1.897±0.406 | 1.967±0.397 | 1.003 | .318 | .808 |
| lh-superiorfrontal | 1.714±0.327 | 1.735±0.303 | 0.396 | .693 | .820 |
| lh-superiorparietal | 1.733±0.373 | 1.774±0.410 | 0.611 | .542 | .808 |
| lh-superiortemporal | 1.490±0.319 | 1.602±0.268 | 2.194 | .030 | .648 |
| lh-supramarginal | 1.635±0.377 | 1.705±0.351 | 1.101 | .273 | .808 |
| lh-frontalpole | 1.824±0.367 | 1.903±0.369 | 1.246 | .215 | .808 |
| lh-temporalpole | 1.165±0.293 | 1.205±0.366 | 0.698 | .487 | .808 |
| lh-transversetemporal | 1.980±0.536 | 2.035±0.506 | 0.614 | .540 | .808 |
| lh-insula | 1.392±0.320 | 1.489±0.419 | 1.507 | .134 | .808 |
| rh-bankssts | 1.650±0.379 | 1.800±0.339 | 2.403 | .018 | .648 |
| rh-caudalanteriorcingulate | 1.481±0.201 | 1.504±0.257 | 0.586 | .559 | .808 |
| rh-caudalmiddlefrontal | 1.882±0.423 | 1.915±0.421 | 0.459 | .647 | .820 |
| rh-cuneus | 1.938±0.382 | 1.975±0.324 | 0.593 | .554 | .808 |
| rh-entorhinal | 0.976±0.205 | 1.024±0.205 | 1.338 | .183 | .808 |
| rh-fusiform | 1.548±0.294 | 1.614±0.296 | 1.289 | .199 | .808 |
| rh-inferiorparietal | 1.624±0.412 | 1.710±0.420 | 1.185 | .238 | .808 |
| rh-inferiortemporal | 1.480±0.321 | 1.575±0.334 | 1.683 | .095 | .808 |
| rh-isthmuscingulate | 1.625±0.310 | 1.693±0.349 | 1.208 | .229 | .808 |
| rh-lateraloccipital | 1.790±0.390 | 1.827±0.362 | 0.568 | .571 | .808 |
| rh-lateralorbitofrontal | 1.681±0.268 | 1.706±0.281 | 0.520 | .604 | .820 |
| rh-lingual | 1.811±0.330 | 1.821±0.268 | 0.190 | .850 | .943 |
| rh-medialorbitofrontal | 1.579±0.245 | 1.620±0.293 | 0.875 | .383 | .808 |
| rh-middletemporal | 1.537±0.373 | 1.645±0.333 | 1.751 | .082 | .808 |
| rh-parahippocampal | 1.243±0.231 | 1.284±0.247 | 0.994 | .322 | .808 |
| rh-paracentral | 1.755±0.274 | 1.768±0.242 | 0.272 | .786 | .888 |
| rh-parsopercularis | 1.784±0.347 | 1.836±0.336 | 0.872 | .385 | .808 |
| rh-parsorbitalis | 1.734±0.418 | 1.733±0.397 | 0.011 | .992 | .999 |
| rh-parstriangularis | 1.858±0.347 | 1.904±0.381 | 0.734 | .464 | .808 |
| rh-pericalcarine | 2.153±0.459 | 2.183±0.443 | 0.393 | .695 | .820 |
| rh-postcentral | 1.786±0.315 | 1.838±0.308 | 0.966 | .336 | .808 |
| rh-posteriorcingulate | 1.802±0.382 | 1.884±0.397 | 1.224 | .223 | .808 |
| rh-precentral | 1.837±0.309 | 1.865±0.256 | 0.565 | .573 | .808 |
| rh-precuneus | 1.791±0.357 | 1.840±0.376 | 0.785 | .434 | .808 |
| rh-rostralanteriorcingulate | 1.467±0.275 | 1.511±0.314 | 0.874 | .384 | .808 |
| rh-rostralmiddlefrontal | 1.864±0.390 | 1.924±0.421 | 0.861 | .391 | .808 |
| rh-superiorfrontal | 1.747±0.327 | 1.757±0.335 | 0.165 | .870 | .943 |
| rh-superiorparietal | 1.708±0.360 | 1.766±0.384 | 0.911 | .364 | .808 |
| rh-superiortemporal | 1.506±0.289 | 1.619±0.292 | 2.262 | .025 | .648 |
| rh-supramarginal | 1.615±0.337 | 1.683±0.355 | 1.135 | .258 | .808 |
| rh-frontalpole | 1.814±0.389 | 1.869±0.405 | 0.802 | .424 | .808 |
| rh-temporalpole | 1.157±0.289 | 1.211±0.327 | 1.014 | .312 | .808 |
| rh-transversetemporal | 1.882±0.518 | 1.946±0.474 | 0.738 | .462 | .808 |

**Table S15.** Comparison of regional AV45 SUVR between genotyped and non-genotyped subgroups

| **Brain region** | **Genotyped group (n=71)** | **genotyped group (n=63)** | **F** | **P** | **FDR-P** |
| --- | --- | --- | --- | --- | --- |
| Left-Amygdala | 1.0222±0.14823 | 0.9348±0.15313 | 3.354 | .001 | .080 |
| Left-Caudate | 1.0200±0.18440 | 0.9837±0.16076 | 1.207 | .230 | .290 |
| Left-Hippocampus | 0.9164±0.11177 | 0.8883±0.10220 | 1.511 | .133 | .236 |
| Left-Pallidum | 1.3675±0.18237 | 1.3803±0.17451 | 0.417 | .677 | .685 |
| Left-Putamen | 1.2731±0.18148 | 1.2315±0.18856 | 1.302 | .195 | .290 |
| Left-Thalamus | 1.2010±0.12668 | 1.1965±0.10785 | 0.218 | .828 | .828 |
| Right-Amygdala | 1.0806±0.14405 | 1.0090±0.15602 | 2.761 | .007 | .140 |
| Right-Caudate | 1.0802±0.18958 | 1.0117±0.18511 | 2.108 | .037 | .202 |
| Right-Hippocampus | 0.9458±0.10569 | 0.9363±0.10238 | 0.528 | .599 | .614 |
| Right-Pallidum | 1.3881±0.18973 | 1.4282±0.16757 | 1.289 | .200 | .290 |
| Right-Putamen | 1.3279±0.18655 | 1.2713±0.20222 | 1.684 | .095 | .217 |
| Right-Thalamus | 1.2453±0.13147 | 1.2327±0.11084 | 0.596 | .553 | .574 |
| lh-bankssts | 1.4964±0.31656 | 1.4263±0.33186 | 1.250 | .213 | .290 |
| lh-caudalanteriorcingulate | 1.2641±0.26723 | 1.1639±0.27431 | 2.140 | .034 | .202 |
| lh-caudalmiddlefrontal | 1.3957±0.31379 | 1.2914±0.30267 | 1.952 | .053 | .202 |
| lh-cuneus | 1.1449±0.25908 | 1.1172±0.24101 | 0.638 | .525 | .553 |
| lh-entorhinal | 0.7793±0.14541 | 0.7583±0.13978 | 0.848 | .398 | .435 |
| lh-frontalpole | 1.3742±0.38263 | 1.2795±0.35451 | 1.480 | .141 | .240 |
| lh-fusiform | 1.2217±0.24870 | 1.1456±0.24398 | 1.784 | .077 | .217 |
| lh-inferiorparietal | 1.3849±0.28500 | 1.2952±0.30245 | 1.768 | .079 | .217 |
| lh-inferiortemporal | 1.2738±0.27259 | 1.1973±0.28535 | 1.587 | .115 | .232 |
| lh-insula | 1.1205±0.21564 | 1.0568±0.20148 | 1.759 | .081 | .217 |
| lh-isthmuscingulate | 1.2363±0.27689 | 1.1584±0.25872 | 1.677 | .096 | .217 |
| lh-lateraloccipital | 1.3130±0.29990 | 1.2749±0.28758 | 0.748 | .456 | .486 |
| lh-lateralorbitofrontal | 1.2926±0.26223 | 1.2196±0.27095 | 1.584 | .116 | .232 |
| lh-lingual | 1.1379±0.26390 | 1.0859±0.24351 | 1.181 | .240 | .290 |
| lh-medialorbitofrontal | 1.2561±0.27738 | 1.1932±0.28331 | 1.297 | .197 | .290 |
| lh-middletemporal | 1.2471±0.28227 | 1.1847±0.27555 | 1.291 | .199 | .290 |
| lh-paracentral | 1.2500±0.26149 | 1.1845±0.23509 | 1.517 | .132 | .236 |
| lh-parahippocampal | 0.9608±0.20604 | 0.9051±0.18372 | 1.643 | .103 | .217 |
| lh-parsopercularis | 1.2853±0.26068 | 1.2357±0.27634 | 1.070 | .287 | .359 |
| lh-parsorbitalis | 1.2815±0.30345 | 1.2296±0.30222 | 0.990 | .324 | .381 |
| lh-parstriangularis | 1.3154±0.27288 | 1.2552±0.28303 | 1.251 | .213 | .290 |
| lh-pericalcarine | 1.3604±0.34980 | 1.3031±0.32793 | 0.975 | .331 | .384 |
| lh-postcentral | 1.2536±0.22645 | 1.1728±0.21857 | 2.097 | .038 | .202 |
| lh-posteriorcingulate | 1.3100±0.29033 | 1.2214±0.28984 | 1.765 | .080 | .217 |
| lh-precentral | 1.2638±0.22660 | 1.2121±0.20776 | 1.369 | .173 | .280 |
| lh-precuneus | 1.3841±0.32497 | 1.2501±0.32625 | 2.378 | .019 | .202 |
| lh-rostralanteriorcingulate | 1.2590±0.28437 | 1.1851±0.29092 | 1.486 | .140 | .240 |
| lh-rostralmiddlefrontal | 1.4671±0.34097 | 1.3665±0.34478 | 1.696 | .092 | .217 |
| lh-superiorfrontal | 1.3248±0.30405 | 1.2241±0.29182 | 1.951 | .053 | .202 |
| lh-superiorparietal | 1.3380±0.27672 | 1.2575±0.27988 | 1.670 | .097 | .217 |
| lh-superiortemporal | 1.1934±0.24252 | 1.1511±0.23705 | 1.019 | .310 | .375 |
| lh-supramarginal | 1.3271±0.25349 | 1.2525±0.26190 | 1.675 | .096 | .217 |
| lh-temporalpole | 0.9895±0.20984 | 0.9445±0.20658 | 1.248 | .214 | .290 |
| lh-transversetemporal | 1.1830±0.21329 | 1.1438±0.25526 | 0.958 | .340* | .389 |
| rh-bankssts | 1.5239±0.32072 | 1.4759±0.34135 | 0.840 | .402 | .435 |
| rh-caudalanteriorcingulate | 1.3560±0.28329 | 1.2094±0.28102 | 3.001 | .003 | .120 |
| rh-caudalmiddlefrontal | 1.3793±0.28736 | 1.2729±0.28558 | 2.147 | .034 | .202 |
| rh-cuneus | 1.1745±0.28692 | 1.1257±0.25252 | 1.040 | .300 | .369 |
| rh-entorhinal | 0.8296±0.17612 | 0.7915±0.15418 | 1.326 | .187 | .290 |
| rh-frontalpole | 1.3476±0.34664 | 1.2734±0.33793 | 1.252 | .213 | .290 |
| rh-fusiform | 1.2470±0.25165 | 1.1597±0.23514 | 2.065 | .041 | .202 |
| rh-inferiorparietal | 1.4028±0.29196 | 1.3327±0.30290 | 1.362 | .175 | .280 |
| rh-inferiortemporal | 1.3211±0.28201 | 1.2348±0.26418 | 1.822 | .071 | .217 |
| rh-insula | 1.1233±0.19908 | 1.0562±0.18721 | 2.003 | .047 | .202 |
| rh-isthmuscingulate | 1.3194±0.28493 | 1.2107±0.26750 | 2.266 | .025 | .202 |
| rh-lateraloccipital | 1.3628±0.31847 | 1.3149±0.27110 | 0.930 | .354 | .397 |
| rh-lateralorbitofrontal | 1.3050±0.25461 | 1.2185±0.25731 | 1.951 | .053 | .202 |
| rh-lingual | 1.1590±0.27337 | 1.0839±0.24872 | 1.656 | .100 | .217 |
| rh-medialorbitofrontal | 1.2664±0.28699 | 1.1840±0.29229 | 1.646 | .102 | .217 |
| rh-middletemporal | 1.3360±0.27247 | 1.2667±0.25586 | 1.512 | .133 | .236 |
| rh-paracentral | 1.3148±0.26881 | 1.2249±0.24893 | 2.001 | .047 | .202 |
| rh-parahippocampal | 1.0505±0.21498 | 0.9860±0.21974 | 1.715 | .089 | .217 |
| rh-parsopercularis | 1.3068±0.25445 | 1.2341±0.24319 | 1.687 | .094 | .217 |
| rh-parsorbitalis | 1.2722±0.29027 | 1.2231±0.26919 | 1.010 | .314 | .375 |
| rh-parstriangularis | 1.3383±0.27571 | 1.2942±0.27591 | 0.924 | .357 | .397 |
| rh-pericalcarine | 1.3843±0.34389 | 1.3011±0.33112 | 1.422 | .157 | .262 |
| rh-postcentral | 1.2621±0.23256 | 1.1834±0.20507 | 2.066 | .041 | .202 |
| rh-posteriorcingulate | 1.3895±0.31028 | 1.2440±0.29639 | 2.768 | .006 | .140 |
| rh-precentral | 1.2607±0.21096 | 1.2044±0.20620 | 1.556 | .122 | .232 |
| rh-precuneus | 1.4049±0.31702 | 1.2614±0.31483 | 2.623 | .010 | .160 |
| rh-rostralanteriorcingulate | 1.3091±0.30835 | 1.2022±0.30861 | 2.003 | .047 | .202 |
| rh-rostralmiddlefrontal | 1.4454±0.32649 | 1.3447±0.32836 | 1.777 | .078 | .217 |
| rh-superiorfrontal | 1.3366±0.29885 | 1.2350±0.28249 | 2.016 | .046 | .202 |
| rh-superiorparietal | 1.3347±0.29147 | 1.2381±0.27230 | 1.976 | .050 | .202 |
| rh-superiortemporal | 1.2157±0.24600 | 1.1673±0.20671 | 1.225 | .223 | .290 |
| rh-supramarginal | 1.3487±0.25083 | 1.2826±0.25286 | 1.517 | .132 | .236 |
| rh-temporalpole | 1.0071±0.22234 | 0.9428±0.17127 | 1.857 | .066 | .217 |
| rh-transversetemporal | 1.0998±0.22913 | 1.0554±0.20864 | 1.167 | .245 | .290 |

**Table S16.** Correlation between Cognitive Domains and Regional Gray Matter Volume in AD group

| **Cognitive domain** | **Brain region** | **Partial r** | **P** | **FDR-P** |
| --- | --- | --- | --- | --- |
| visuospatial | rh-inferiorparietal | 0.541 | 6.28E-08 | 2.70E-05 |
| visuospatial | lh-inferiorparietal | 0.499 | 8.67E-07 | 0.000186512 |
| visuospatial | lh-middletemporal | 0.489 | 1.50E-06 | 0.000215714 |
| visuospatial | Left-Amygdala | 0.444 | 1.66E-05 | 0.001207423 |
| visuospatial | Right-Accumbens-area | 0.439 | 2.07E-05 | 0.001207423 |
| visuospatial | Right-Amygdala | 0.436 | 2.40E-05 | 0.001207423 |
| visuospatial | rh-precuneus | 0.434 | 2.71E-05 | 0.001207423 |
| visuospatial | lh-precuneus | 0.418 | 5.56E-05 | 0.001960184 |
| visuospatial | Left-Hippocampus | 0.417 | 5.93E-05 | 0.001960184 |
| visuospatial | lh-posteriorcingulate | 0.411 | 7.65E-05 | 0.002349956 |
| visuospatial | rh-middletemporal | 0.408 | 8.88E-05 | 0.002547014 |
| visuospatial | lh-superiortemporal | 0.403 | 0.000108897 | 0.002812695 |
| visuospatial | lh-bankssts | 0.402 | 0.000115015 | 0.002812695 |
| visuospatial | lh-inferiortemporal | 0.401 | 0.000117741 | 0.002812695 |
| visuospatial | Right-Hippocampus | 0.393 | 0.000165662 | 0.003561729 |
| visuospatial | rh-superiorparietal | 0.390 | 0.000188226 | 0.00366811 |
| visuospatial | Left-Accumbens-area | 0.377 | 0.00032265 | 0.005468183 |
| visuospatial | lh-caudalmiddlefrontal | 0.376 | 0.000333564 | 0.005468183 |
| visuospatial | lh-supramarginal | 0.375 | 0.000341745 | 0.005468183 |
| visuospatial | lh-rostralmiddlefrontal | 0.375 | 0.000343351 | 0.005468183 |
| visuospatial | rh-superiortemporal | 0.371 | 0.00041051 | 0.005694176 |
| visuospatial | lh-superiorparietal | 0.363 | 0.000547039 | 0.006720767 |
| visuospatial | rh-supramarginal | 0.356 | 0.000707768 | 0.007906677 |
| visuospatial | lh-rostralanteriorcingulate | 0.351 | 0.000869499 | 0.009347112 |
| visuospatial | rh-bankssts | 0.345 | 0.001067334 | 0.010198968 |
| visuospatial | rh-rostralmiddlefrontal | 0.341 | 0.001247079 | 0.010943752 |
| visuospatial | lh-precentral | 0.337 | 0.001414153 | 0.011260845 |
| visuospatial | lh-lateralorbitofrontal | 0.336 | 0.001468274 | 0.011373465 |
| visuospatial | lh-parsorbitalis | 0.336 | 0.001481195 | 0.011373465 |
| visuospatial | rh-caudalmiddlefrontal | 0.334 | 0.001555839 | 0.011534671 |
| visuospatial | lh-fusiform | 0.326 | 0.002047616 | 0.013183494 |
| visuospatial | rh-medialorbitofrontal | 0.318 | 0.002684139 | 0.015186573 |
| visuospatial | rh-superiorfrontal | 0.314 | 0.003022433 | 0.016045016 |
| visuospatial | rh-insula | 0.312 | 0.003265627 | 0.01691831 |
| visuospatial | rh-precentral | 0.302 | 0.00441233 | 0.020401095 |
| visuospatial | lh-medialorbitofrontal | 0.301 | 0.004673988 | 0.021155946 |
| visuospatial | rh-inferiortemporal | 0.299 | 0.004909569 | 0.021764069 |
| visuospatial | rh-transversetemporal | 0.287 | 0.007123897 | 0.027597078 |
| visuospatial | lh-isthmuscingulate | 0.284 | 0.007702813 | 0.02931159 |
| visuospatial | lh-superiorfrontal | 0.269 | 0.011660819 | 0.039794858 |
| visuospatial | Right-Caudate | 0.266 | 0.012694905 | 0.04231635 |
| visuospatial | lh-insula | 0.264 | 0.013443645 | 0.044433037 |
| visuospatial | rh-parsopercularis | 0.264 | 0.013579005 | 0.044433037 |
| visuospatial | rh-parsorbitalis | 0.263 | 0.013715443 | 0.044433037 |
| visuospatial | lh-postcentral | 0.258 | 0.015709984 | 0.047909881 |
| visuospatial | Left-Putamen | 0.277 | 0.009363166 | 0.034411637 |
| executive fuction | Right-Amygdala | 0.474 | 3.45E-06 | 0.00037062 |
| executive fuction | Left-Amygdala | 0.440 | 1.98E-05 | 0.001207423 |
| executive fuction | lh-precuneus | 0.400 | 0.000124847 | 0.002825478 |
| executive fuction | lh-fusiform | 0.371 | 0.000405713 | 0.005694176 |
| executive fuction | Right-Hippocampus | 0.369 | 0.000430405 | 0.005783567 |
| executive fuction | lh-cuneus | 0.368 | 0.000448475 | 0.005843763 |
| executive fuction | lh-inferiorparietal | 0.360 | 0.000626032 | 0.007275504 |
| executive fuction | lh-middletemporal | 0.345 | 0.001058254 | 0.010198968 |
| executive fuction | rh-middletemporal | 0.344 | 0.001102588 | 0.010306802 |
| executive fuction | rh-caudalmiddlefrontal | 0.339 | 0.001313924 | 0.010952896 |
| executive fuction | rh-inferiortemporal | 0.339 | 0.001324536 | 0.010952896 |
| executive fuction | lh-isthmuscingulate | 0.337 | 0.001392733 | 0.011260845 |
| executive fuction | Right-Putamen | 0.332 | 0.001699293 | 0.011978625 |
| executive fuction | rh-inferiorparietal | 0.328 | 0.001900824 | 0.012973876 |
| executive fuction | lh-bankssts | 0.324 | 0.002226434 | 0.013676669 |
| executive fuction | Left-Accumbens-area | 0.323 | 0.002284123 | 0.013833419 |
| executive fuction | lh-inferiortemporal | 0.317 | 0.002752788 | 0.015363499 |
| executive fuction | rh-pericalcarine | 0.316 | 0.002877833 | 0.015664153 |
| executive fuction | rh-precuneus | 0.308 | 0.003739379 | 0.01871364 |
| executive fuction | rh-fusiform | 0.307 | 0.00379066 | 0.01871364 |
| executive fuction | Left-Hippocampus | 0.305 | 0.004036742 | 0.019503359 |
| executive fuction | rh-lateraloccipital | 0.304 | 0.004231705 | 0.019778623 |
| executive fuction | lh-rostralanteriorcingulate | 0.294 | 0.005722768 | 0.024364259 |
| executive fuction | lh-pericalcarine | 0.290 | 0.006379955 | 0.026378658 |
| executive fuction | rh-superiortemporal | 0.288 | 0.006908617 | 0.027254179 |
| executive fuction | rh-superiorparietal | 0.283 | 0.007953458 | 0.029999886 |
| executive fuction | lh-superiortemporal | 0.282 | 0.008071638 | 0.030180908 |
| executive fuction | lh-supramarginal | 0.277 | 0.009359919 | 0.034411637 |
| executive fuction | lh-temporalpole | 0.276 | 0.009666943 | 0.035226994 |
| executive fuction | lh-medialorbitofrontal | 0.271 | 0.010978028 | 0.038693051 |
| executive fuction | rh-bankssts | 0.270 | 0.011508347 | 0.039588713 |
| executive fuction | lh-posteriorcingulate | 0.261 | 0.014591068 | 0.045464923 |
| executive fuction | rh-insula | 0.260 | 0.014840373 | 0.045909069 |
| executive fuction | Left-Putamen | 0.258 | 0.015933861 | 0.048225558 |
| executive fuction | rh-isthmuscingulate | 0.258 | 0.016037802 | 0.048225558 |
| attention | Right-Accumbens-area | 0.433 | 2.81E-05 | 0.001207423 |
| attention | rh-precentral | 0.391 | 0.000181635 | 0.00366811 |
| attention | rh-supramarginal | 0.365 | 0.000499569 | 0.00631808 |
| attention | Right-Amygdala | 0.356 | 0.000717117 | 0.007906677 |
| attention | Right-Putamen | 0.343 | 0.001156983 | 0.010585164 |
| attention | Left-Accumbens-area | 0.341 | 0.001244673 | 0.010943752 |
| attention | lh-precuneus | 0.340 | 0.001289009 | 0.010952896 |
| attention | rh-caudalmiddlefrontal | 0.333 | 0.001645048 | 0.011978625 |
| attention | lh-fusiform | 0.328 | 0.001900152 | 0.012973876 |
| attention | lh-inferiorparietal | 0.327 | 0.002008293 | 0.013183494 |
| attention | lh-inferiortemporal | 0.327 | 0.002010907 | 0.013183494 |
| attention | Left-Amygdala | 0.326 | 0.002093054 | 0.013183494 |
| attention | lh-rostralanteriorcingulate | 0.325 | 0.002113974 | 0.013183494 |
| attention | rh-superiorparietal | 0.320 | 0.00251851 | 0.014601567 |
| attention | Left-Putamen | 0.317 | 0.002786867 | 0.015363499 |
| attention | lh-bankssts | 0.315 | 0.00296447 | 0.015934025 |
| attention | rh-superiortemporal | 0.310 | 0.003487937 | 0.017854915 |
| attention | rh-bankssts | 0.304 | 0.004192074 | 0.019778623 |
| attention | rh-postcentral | 0.302 | 0.004479429 | 0.020491004 |
| attention | rh-insula | 0.299 | 0.00488705 | 0.021764069 |
| attention | lh-supramarginal | 0.299 | 0.004969308 | 0.021804105 |
| attention | lh-superiortemporal | 0.295 | 0.005492377 | 0.02385578 |
| attention | rh-inferiorparietal | 0.293 | 0.005920614 | 0.024959453 |
| attention | lh-insula | 0.289 | 0.006540886 | 0.026786487 |
| attention | rh-middletemporal | 0.288 | 0.006776457 | 0.02723249 |
| attention | lh-precentral | 0.288 | 0.0068924 | 0.027254179 |
| attention | Left-Hippocampus | 0.274 | 0.010138553 | 0.036635108 |
| attention | lh-posteriorcingulate | 0.268 | 0.012052184 | 0.040706394 |
| attention | lh-postcentral | 0.262 | 0.014292181 | 0.045188513 |
| attention | Right-Hippocampus | 0.261 | 0.014556582 | 0.045464923 |
| language | lh-lateralorbitofrontal | 0.389 | 0.000196201 | 0.00366811 |
| language | rh-caudalmiddlefrontal | 0.371 | 0.000400862 | 0.005694176 |
| language | lh-superiortemporal | 0.360 | 0.000609038 | 0.00727462 |
| language | lh-precuneus | 0.347 | 0.000975707 | 0.010198968 |
| language | Left-Amygdala | 0.346 | 0.001026442 | 0.010198968 |
| language | rh-supramarginal | 0.335 | 0.001511384 | 0.011401672 |
| language | rh-rostralmiddlefrontal | 0.332 | 0.001675386 | 0.011978625 |
| language | lh-fusiform | 0.325 | 0.002115491 | 0.013183494 |
| language | Right-Accumbens-area | 0.321 | 0.00240935 | 0.014192059 |
| language | Left-Accumbens-area | 0.320 | 0.002546785 | 0.014601567 |
| language | lh-superiorparietal | 0.312 | 0.003236497 | 0.01691831 |
| language | lh-medialorbitofrontal | 0.294 | 0.005633943 | 0.024225955 |
| language | lh-rostralmiddlefrontal | 0.289 | 0.006654388 | 0.026994215 |
| language | rh-superiorfrontal | 0.287 | 0.006984628 | 0.027303546 |
| language | lh-temporalpole | 0.285 | 0.007505655 | 0.028816354 |
| language | rh-middletemporal | 0.273 | 0.010505369 | 0.03764424 |
| language | rh-superiorparietal | 0.272 | 0.010816184 | 0.038437678 |
| language | lh-posteriorcingulate | 0.271 | 0.011198026 | 0.039147569 |
| language | lh-inferiortemporal | 0.270 | 0.011481543 | 0.039588713 |
| language | lh-caudalmiddlefrontal | 0.268 | 0.012117252 | 0.040706394 |
| language | rh-medialorbitofrontal | 0.263 | 0.013790622 | 0.044433037 |
| language | lh-precentral | 0.263 | 0.013846574 | 0.044433037 |
| language | rh-parsopercularis | 0.262 | 0.01412038 | 0.044976025 |
| language | rh-precuneus | 0.259 | 0.015474272 | 0.047528121 |

### Table S17. Correlation between Cognitive Domains and Regional FDG Metabolism in AD group

| **Cognitive domain** | **Brain region** | **Partial r** | **P** | **FDR-P** |
| --- | --- | --- | --- | --- |
| visuospatial | rh-superiortemporal | 0.4430 | 0.0000 | 0.0015 |
| visuospatial | lh-middletemporal | 0.4395 | 0.097E-07 | 0.0019 |
| visuospatial | lh-inferiorparietal | 0.4378 | 0.00002 | 0.0015 |
| visuospatial | lh-parsopercularis | 0.4376 | 0.00001 | 0.0015 |
| visuospatial | rh-inferiortemporal | 0.4313 | 0.00001 | 0.0017 |
| visuospatial | lh-bankssts | 0.4296 | 0.00001 | 0.0017 |
| visuospatial | rh-inferiorparietal | 0.4250 | 0.00001 | 0.0019 |
| visuospatial | rh-supramarginal | 0.3965 | 0.0001 | 0.0049 |
| visuospatial | lh-lateralorbitofrontal | 0.3952 | 0.0001 | 0.0049 |
| visuospatial | rh-middletemporal | 0.3798 | 0.0003 | 0.0086 |
| visuospatial | rh-superiortemporal | 0.3730 | 0.0003 | 0.0099 |
| visuospatial | lh-parstriangularis | 0.3665 | 0.0004 | 0.0104 |
| visuospatial | lh-precuneus | 0.3554 | 0.0007 | 0.0136 |
| visuospatial | rh-precuneus | 0.3455 | 0.0010 | 0.0145 |
| visuospatial | lh-rostralmiddlefrontal | 0.3437 | 0.0010 | 0.0145 |
| visuospatial | rh-parsopercularis | 0.2972 | 0.0049 | 0.0412 |
| visuospatial | lh-isthmuscingulate | 0.3334 | 0.0015 | 0.0177 |
| visuospatial | rh-caudalmiddlefrontal | 0.3321 | 0.0016 | 0.0181 |
| visuospatial | lh-caudalmiddlefrontal | 0.3301 | 0.0017 | 0.0189 |
| visuospatial | rh-parstriangularis | 0.3033 | 0.0041 | 0.0370 |
| visuospatial | lh-transversetemporal | 0.2955 | 0.0052 | 0.0419 |
| visuospatial | rh-isthmuscingulate | 0.2886 | 0.0064 | 0.0464 |
| visuospatial | lh-frontalpole | 0.2883 | 0.0065 | 0.0464 |
| visuospatial | rh-transversetemporal | 0.2869 | 0.0067 | 0.0468 |
| visuospatial | rh-lateralorbitofrontal | 0.2865 | 0.0068 | 0.0468 |
| language | lh-superiortemporal | 0.4121 | 0.0001 | 0.0030 |
| language | lh-parsopercularis | 0.4030 | 0.0001 | 0.0033 |
| language | rh-caudalmiddlefrontal | 0.3511 | 0.0008 | 0.0145 |
| language | lh-inferiorparietal | 0.3502 | 0.0008 | 0.0145 |
| language | lh-caudalmiddlefrontal | 0.3485 | 0.0009 | 0.0145 |
| language | lh-rostralmiddlefrontal | 0.3780 | 0.0003 | 0.0087 |
| language | lh-supramarginal | 0.3715 | 0.0004 | 0.0099 |
| language | Left-Caudate | 0.3407 | 0.0012 | 0.0157 |
| language | lh-parsorbitalis | 0.3698 | 0.0004 | 0.0100 |
| language | rh-parsorbitalis | 0.3660 | 0.0005 | 0.0104 |
| language | lh-parstriangularis | 0.3172 | 0.0026 | 0.0283 |
| language | rh-inferiorparietal | 0.3634 | 0.0005 | 0.0110 |
| language | rh-supramarginal | 0.3563 | 0.0007 | 0.0136 |
| language | rh-rostralmiddlefrontal | 0.3464 | 0.0009 | 0.0145 |
| language | lh-transversetemporal | 0.3446 | 0.0010 | 0.0145 |
| language | lh-lateralorbitofrontal | 0.3437 | 0.0010 | 0.0145 |
| language | lh-frontalpole | 0.3362 | 0.0014 | 0.0174 |
| language | rh-bankssts | 0.3354 | 0.0014 | 0.0174 |
| language | lh-inferiortemporal | 0.3341 | 0.0015 | 0.0177 |
| language | rh-parstriangularis | 0.3004 | 0.0045 | 0.0390 |
| language | rh-lateraloccipital | 0.2856 | 0.0070 | 0.0469 |
| language | rh-parsopercularis | 0.3085 | 0.0035 | 0.0343 |
| language | rh-inferiortemporal | 0.3080 | 0.0035 | 0.0343 |
| language | rh-transversetemporal | 0.3073 | 0.0036 | 0.0344 |
| language | lh-superiorfrontal | 0.3056 | 0.0038 | 0.0356 |
| language | rh-precuneus | 0.3031 | 0.0041 | 0.0370 |
| language | rh-frontalpole | 0.2926 | 0.0057 | 0.0433 |
| language | lh-precuneus | 0.2921 | 0.0057 | 0.0433 |
| memory | lh-posteriorcingulate | 0.2959 | 0.0051 | 0.0419 |
| memory | rh-posteriorcingulate | 0.3446 | 0.0010 | 0.0145 |
| executive fuction | lh-lateralorbitofrontal | 0.3001 | 0.0045 | 0.0390 |
| executive fuction | lh-transversetemporal | 0.3381 | 0.0013 | 0.0167 |
| executive fuction | lh-inferiorparietal | 0.3476 | 0.0009 | 0.0145 |
| executive fuction | rh-bankssts | 0.3166 | 0.0026 | 0.0283 |
| executive fuction | rh-parsorbitalis | 0.3084 | 0.0035 | 0.0343 |
| executive fuction | rh-transversetemporal | 0.2979 | 0.0048 | 0.0411 |
| executive fuction | lh-inferiorparietal | 0.2944 | 0.0054 | 0.0425 |
| executive fuction | lh-parsopercularis | 0.2899 | 0.0062 | 0.0456 |
| executive fuction | lh-precuneus | 0.2868 | 0.0067 | 0.0468 |
| executive fuction | rh-inferiorparietal | 0.2854 | 0.0070 | 0.0469 |

**Table S18.** Correlation between Cognitive Domains and Regional Gray Matter Volume in non-AD group

| **Cognitive domain** | **Brain region** | **Partial r** | **P** | **FDR-p** |
| --- | --- | --- | --- | --- |
| visuospatial | lh-bankssts | 0.074726 | 0.655683 | 0.966672 |
| visuospatial | lh-caudalanteriorcingulate | 0.267891 | 0.103928 | 0.663823 |
| visuospatial | lh-caudalmiddlefrontal | 0.35241 | 0.030008 | 0.663823 |
| visuospatial | lh-cuneus | -0.032561 | 0.846127 | 0.98315 |
| visuospatial | lh-entorhinal | 0.044996 | 0.788509 | 0.98315 |
| visuospatial | lh-fusiform | 0.145669 | 0.382861 | 0.884456 |
| visuospatial | lh-inferiorparietal | 0.19231 | 0.247389 | 0.749137 |
| visuospatial | lh-inferiortemporal | 0.159829 | 0.337801 | 0.853848 |
| visuospatial | lh-isthmuscingulate | 0.261899 | 0.112198 | 0.663823 |
| visuospatial | lh-lateraloccipital | 0.016096 | 0.923591 | 0.98315 |
| visuospatial | lh-lateralorbitofrontal | 0.157482 | 0.345035 | 0.853848 |
| visuospatial | lh-lingual | 0.082267 | 0.623412 | 0.966672 |
| visuospatial | lh-medialorbitofrontal | -0.007563 | 0.964058 | 0.989368 |
| visuospatial | lh-middletemporal | 0.242224 | 0.142863 | 0.663823 |
| visuospatial | lh-parahippocampal | -0.047901 | 0.775197 | 0.98315 |
| visuospatial | lh-paracentral | 0.251642 | 0.127496 | 0.663823 |
| visuospatial | lh-parsopercularis | 0.247145 | 0.134672 | 0.663823 |
| visuospatial | lh-parsorbitalis | -0.021516 | 0.897976 | 0.98315 |
| visuospatial | lh-parstriangularis | 0.234842 | 0.155829 | 0.663823 |
| visuospatial | lh-pericalcarine | 0.127252 | 0.446459 | 0.890771 |
| visuospatial | lh-postcentral | 0.144512 | 0.386692 | 0.884456 |
| visuospatial | lh-posteriorcingulate | 0.126856 | 0.447887 | 0.890771 |
| visuospatial | lh-precentral | 0.304353 | 0.063192 | 0.663823 |
| visuospatial | lh-precuneus | 0.361535 | 0.025731 | 0.663823 |
| visuospatial | lh-rostralanteriorcingulate | 0.372206 | 0.021383 | 0.652511 |
| visuospatial | lh-rostralmiddlefrontal | 0.159162 | 0.339848 | 0.853848 |
| visuospatial | lh-superiorfrontal | 0.240013 | 0.14666 | 0.663823 |
| visuospatial | lh-superiorparietal | 0.38342 | 0.017491 | 0.652511 |
| visuospatial | lh-superiortemporal | 0.171205 | 0.304074 | 0.830048 |
| visuospatial | lh-supramarginal | 0.090481 | 0.589032 | 0.947557 |
| visuospatial | lh-frontalpole | -0.099263 | 0.553232 | 0.936574 |
| visuospatial | lh-temporalpole | 0.257004 | 0.119316 | 0.663823 |
| visuospatial | lh-transversetemporal | -0.130201 | 0.435905 | 0.890771 |
| visuospatial | lh-insula | 0.142046 | 0.394934 | 0.886319 |
| visuospatial | rh-bankssts | 0.150967 | 0.365605 | 0.884456 |
| visuospatial | rh-caudalanteriorcingulate | -0.055144 | 0.742286 | 0.980556 |
| visuospatial | rh-caudalmiddlefrontal | 0.259567 | 0.115548 | 0.663823 |
| visuospatial | rh-cuneus | -0.187365 | 0.259987 | 0.759409 |
| visuospatial | rh-entorhinal | 0.056761 | 0.735001 | 0.980556 |
| visuospatial | rh-fusiform | 0.248886 | 0.13186 | 0.663823 |
| visuospatial | rh-inferiorparietal | 0.038655 | 0.817772 | 0.98315 |
| visuospatial | rh-inferiortemporal | 0.253808 | 0.124142 | 0.663823 |
| visuospatial | rh-isthmuscingulate | 0.21198 | 0.20137 | 0.6983 |
| visuospatial | rh-lateraloccipital | -0.018965 | 0.910021 | 0.98315 |
| visuospatial | rh-lateralorbitofrontal | 0.080566 | 0.630636 | 0.966672 |
| visuospatial | rh-lingual | 0.094453 | 0.572712 | 0.94582 |
| visuospatial | rh-medialorbitofrontal | -0.020696 | 0.901848 | 0.98315 |
| visuospatial | rh-middletemporal | 0.209159 | 0.207573 | 0.708384 |
| visuospatial | rh-parahippocampal | 0.027649 | 0.869121 | 0.98315 |
| visuospatial | rh-paracentral | 0.194254 | 0.242552 | 0.749137 |
| visuospatial | rh-parsopercularis | 0.378393 | 0.019155 | 0.652511 |
| visuospatial | rh-parsorbitalis | -0.049925 | 0.76596 | 0.980556 |
| visuospatial | rh-parstriangularis | 0.276654 | 0.092677 | 0.663823 |
| visuospatial | rh-pericalcarine | 0.034241 | 0.838287 | 0.98315 |
| visuospatial | rh-postcentral | 0.204988 | 0.216987 | 0.712246 |
| visuospatial | rh-posteriorcingulate | 0.02143 | 0.89838 | 0.98315 |
| visuospatial | rh-precentral | 0.206924 | 0.212581 | 0.712246 |
| visuospatial | rh-precuneus | 0.316261 | 0.053062 | 0.663823 |
| visuospatial | rh-rostralanteriorcingulate | -0.161445 | 0.332875 | 0.853848 |
| visuospatial | rh-rostralmiddlefrontal | 0.160008 | 0.337253 | 0.853848 |
| visuospatial | rh-superiorfrontal | 0.382077 | 0.017923 | 0.652511 |
| visuospatial | rh-superiorparietal | 0.090218 | 0.59012 | 0.947557 |
| visuospatial | rh-superiortemporal | 0.170919 | 0.304894 | 0.830048 |
| visuospatial | rh-supramarginal | 0.030559 | 0.855481 | 0.98315 |
| visuospatial | rh-frontalpole | -0.268916 | 0.102561 | 0.663823 |
| visuospatial | rh-temporalpole | 0.015329 | 0.927218 | 0.98315 |
| visuospatial | rh-transversetemporal | 0.169959 | 0.30766 | 0.830048 |
| visuospatial | rh-insula | 0.181699 | 0.274934 | 0.772691 |
| visuospatial | Left-Thalamus | 0.31384 | 0.055009 | 0.663823 |
| visuospatial | Left-Caudate | 0.327583 | 0.044678 | 0.663823 |
| visuospatial | Left-Putamen | 0.311643 | 0.056825 | 0.663823 |
| visuospatial | Left-Pallidum | 0.073865 | 0.659412 | 0.966672 |
| visuospatial | Left-Hippocampus | 0.277869 | 0.091196 | 0.663823 |
| visuospatial | Left-Amygdala | 0.100916 | 0.54661 | 0.936156 |
| visuospatial | Right-Thalamus | 0.255016 | 0.122302 | 0.663823 |
| visuospatial | Right-Caudate | 0.281608 | 0.086747 | 0.663823 |
| visuospatial | Right-Putamen | 0.235887 | 0.153944 | 0.663823 |
| visuospatial | Right-Pallidum | 0.169579 | 0.308758 | 0.830048 |
| visuospatial | Right-Hippocampus | 0.146129 | 0.381346 | 0.884456 |
| visuospatial | Right-Amygdala | -0.003647 | 0.982661 | 0.995431 |
| memory | lh-bankssts | 0.032088 | 0.848333 | 0.98315 |
| memory | lh-caudalanteriorcingulate | 0.018715 | 0.9112 | 0.98315 |
| memory | lh-caudalmiddlefrontal | 0.238604 | 0.149119 | 0.663823 |
| memory | lh-cuneus | 0.256029 | 0.120774 | 0.663823 |
| memory | lh-entorhinal | -0.146753 | 0.379293 | 0.884456 |
| memory | lh-fusiform | -0.073816 | 0.659622 | 0.966672 |
| memory | lh-inferiorparietal | 0.020489 | 0.902821 | 0.98315 |
| memory | lh-inferiortemporal | -0.092386 | 0.581179 | 0.947557 |
| memory | lh-isthmuscingulate | 0.082059 | 0.624294 | 0.966672 |
| memory | lh-lateraloccipital | -0.003975 | 0.981102 | 0.995431 |
| memory | lh-lateralorbitofrontal | 0.12482 | 0.455263 | 0.890771 |
| memory | lh-lingual | 0.058742 | 0.726103 | 0.980556 |
| memory | lh-medialorbitofrontal | -0.047092 | 0.778899 | 0.98315 |
| memory | lh-middletemporal | 0.031572 | 0.850746 | 0.98315 |
| memory | lh-parahippocampal | 0.107987 | 0.518713 | 0.914701 |
| memory | lh-paracentral | 0.076318 | 0.648816 | 0.966672 |
| memory | lh-parsopercularis | 0.236002 | 0.153737 | 0.663823 |
| memory | lh-parsorbitalis | -0.044704 | 0.789854 | 0.98315 |
| memory | lh-parstriangularis | 0.119893 | 0.473392 | 0.908968 |
| memory | lh-pericalcarine | 0.229515 | 0.165702 | 0.663823 |
| memory | lh-postcentral | 0.042762 | 0.798792 | 0.98315 |
| memory | lh-posteriorcingulate | -0.036687 | 0.826906 | 0.98315 |
| memory | lh-precentral | 0.186195 | 0.263027 | 0.759409 |
| memory | lh-precuneus | 0.262451 | 0.111416 | 0.663823 |
| memory | lh-rostralanteriorcingulate | 0.035214 | 0.833755 | 0.98315 |
| memory | lh-rostralmiddlefrontal | 0.125489 | 0.452832 | 0.890771 |
| memory | lh-superiorfrontal | 0.253687 | 0.124328 | 0.663823 |
| memory | lh-superiorparietal | 0.222577 | 0.179226 | 0.663823 |
| memory | lh-superiortemporal | 0.074797 | 0.655378 | 0.966672 |
| memory | lh-supramarginal | 0.124689 | 0.455743 | 0.890771 |
| memory | lh-frontalpole | -0.106553 | 0.524313 | 0.917522 |
| memory | lh-temporalpole | 0.085118 | 0.611383 | 0.966524 |
| memory | lh-transversetemporal | 0.036019 | 0.830012 | 0.98315 |
| memory | lh-insula | -0.031531 | 0.850937 | 0.98315 |
| memory | rh-bankssts | -0.026804 | 0.873087 | 0.98315 |
| memory | rh-caudalanteriorcingulate | -0.09836 | 0.556866 | 0.939029 |
| memory | rh-caudalmiddlefrontal | -0.105354 | 0.529019 | 0.917991 |
| memory | rh-cuneus | 0.11024 | 0.509977 | 0.914701 |
| memory | rh-entorhinal | -0.129093 | 0.439855 | 0.890771 |
| memory | rh-fusiform | 0.145694 | 0.382779 | 0.884456 |
| memory | rh-inferiorparietal | -0.228355 | 0.167911 | 0.663823 |
| memory | rh-inferiortemporal | 0.117735 | 0.481448 | 0.908968 |
| memory | rh-isthmuscingulate | -0.022351 | 0.894041 | 0.98315 |
| memory | rh-lateraloccipital | -0.023209 | 0.889995 | 0.98315 |
| memory | rh-lateralorbitofrontal | 0.00261 | 0.98759 | 0.995431 |
| memory | rh-lingual | 0.166168 | 0.318735 | 0.842904 |
| memory | rh-medialorbitofrontal | -0.013436 | 0.936187 | 0.98315 |
| memory | rh-middletemporal | 0.058581 | 0.726821 | 0.980556 |
| memory | rh-parahippocampal | 0.058447 | 0.727426 | 0.980556 |
| memory | rh-paracentral | -0.03613 | 0.829493 | 0.98315 |
| memory | rh-parsopercularis | 0.220108 | 0.184222 | 0.671318 |
| memory | rh-parsorbitalis | -0.026863 | 0.872808 | 0.98315 |
| memory | rh-parstriangularis | 0.200265 | 0.227997 | 0.729684 |
| memory | rh-pericalcarine | 0.30243 | 0.064964 | 0.663823 |
| memory | rh-postcentral | -0.007694 | 0.963432 | 0.989368 |
| memory | rh-posteriorcingulate | 0.108168 | 0.51801 | 0.914701 |
| memory | rh-precentral | 0.066324 | 0.692379 | 0.972349 |
| memory | rh-precuneus | 0.214206 | 0.196567 | 0.692819 |
| memory | rh-rostralanteriorcingulate | -0.243664 | 0.14043 | 0.663823 |
| memory | rh-rostralmiddlefrontal | 0.130494 | 0.434863 | 0.890771 |
| memory | rh-superiorfrontal | 0.255339 | 0.121813 | 0.663823 |
| memory | rh-superiorparietal | 0.012545 | 0.94041 | 0.98315 |
| memory | rh-superiortemporal | -0.023708 | 0.887648 | 0.98315 |
| memory | rh-supramarginal | -0.170596 | 0.305824 | 0.830048 |
| memory | rh-frontalpole | -0.136365 | 0.414298 | 0.890771 |
| memory | rh-temporalpole | -0.122298 | 0.464494 | 0.903767 |
| memory | rh-transversetemporal | 0.011867 | 0.943628 | 0.98315 |
| memory | rh-insula | -0.008227 | 0.960901 | 0.989368 |
| memory | Left-Thalamus | 0.042021 | 0.802206 | 0.98315 |
| memory | Left-Caudate | 0.2421 | 0.143074 | 0.663823 |
| memory | Left-Putamen | 0.134164 | 0.421944 | 0.890771 |
| memory | Left-Pallidum | -0.078414 | 0.639819 | 0.966672 |
| memory | Left-Hippocampus | 0.169546 | 0.308855 | 0.830048 |
| memory | Left-Amygdala | 0.031068 | 0.853101 | 0.98315 |
| memory | Right-Thalamus | 0.103878 | 0.534836 | 0.919918 |
| memory | Right-Caudate | 0.217532 | 0.18954 | 0.673573 |
| memory | Right-Putamen | 0.093578 | 0.57629 | 0.94582 |
| memory | Right-Pallidum | -0.011897 | 0.943484 | 0.98315 |
| memory | Right-Hippocampus | 0.13231 | 0.428445 | 0.890771 |
| memory | Right-Amygdala | -0.0794 | 0.635605 | 0.966672 |
| attention | lh-bankssts | 0.233078 | 0.15905 | 0.663823 |
| attention | lh-caudalanteriorcingulate | -0.097708 | 0.559495 | 0.939778 |
| attention | lh-caudalmiddlefrontal | -0.034389 | 0.837598 | 0.98315 |
| attention | lh-cuneus | 0.040825 | 0.807729 | 0.98315 |
| attention | lh-entorhinal | 0.011247 | 0.946568 | 0.98315 |
| attention | lh-fusiform | 0.129339 | 0.438977 | 0.890771 |
| attention | lh-inferiorparietal | 0.104703 | 0.531581 | 0.917991 |
| attention | lh-inferiortemporal | 0.169912 | 0.307795 | 0.830048 |
| attention | lh-isthmuscingulate | 0.230762 | 0.16335 | 0.663823 |
| attention | lh-lateraloccipital | 0.049987 | 0.765677 | 0.980556 |
| attention | lh-lateralorbitofrontal | 0.138898 | 0.405597 | 0.888827 |
| attention | lh-lingual | 0.318489 | 0.051319 | 0.663823 |
| attention | lh-medialorbitofrontal | 0.02956 | 0.86016 | 0.98315 |
| attention | lh-middletemporal | 0.117034 | 0.484078 | 0.908968 |
| attention | lh-parahippocampal | 0.106401 | 0.524908 | 0.917522 |
| attention | lh-paracentral | 0.075924 | 0.650516 | 0.966672 |
| attention | lh-parsopercularis | 0.245947 | 0.136634 | 0.663823 |
| attention | lh-parsorbitalis | 0.029935 | 0.858402 | 0.98315 |
| attention | lh-parstriangularis | 0.265723 | 0.106865 | 0.663823 |
| attention | lh-pericalcarine | 0.207254 | 0.211837 | 0.712246 |
| attention | lh-postcentral | -0.001342 | 0.993618 | 0.995431 |
| attention | lh-posteriorcingulate | 0.001612 | 0.992338 | 0.995431 |
| attention | lh-precentral | 0.045207 | 0.787544 | 0.98315 |
| attention | lh-precuneus | -0.012181 | 0.942136 | 0.98315 |
| attention | lh-rostralanteriorcingulate | 0.374251 | 0.020623 | 0.652511 |
| attention | lh-rostralmiddlefrontal | 0.236195 | 0.15339 | 0.663823 |
| attention | lh-superiorfrontal | 0.320781 | 0.049576 | 0.663823 |
| attention | lh-superiorparietal | 0.141803 | 0.395752 | 0.886319 |
| attention | lh-superiortemporal | 0.072545 | 0.66514 | 0.966672 |
| attention | lh-supramarginal | 0.203788 | 0.219749 | 0.71585 |
| attention | lh-frontalpole | 0.273839 | 0.096185 | 0.663823 |
| attention | lh-temporalpole | 0.112537 | 0.501143 | 0.914701 |
| attention | lh-transversetemporal | 0.15197 | 0.362393 | 0.884456 |
| attention | lh-insula | 0.081918 | 0.62489 | 0.966672 |
| attention | rh-bankssts | 0.002094 | 0.990045 | 0.995431 |
| attention | rh-caudalanteriorcingulate | 0.144623 | 0.386326 | 0.884456 |
| attention | rh-caudalmiddlefrontal | 0.09696 | 0.562518 | 0.941178 |
| attention | rh-cuneus | -0.295782 | 0.071388 | 0.663823 |
| attention | rh-entorhinal | 0.281896 | 0.08641 | 0.663823 |
| attention | rh-fusiform | 0.199806 | 0.229087 | 0.729684 |
| attention | rh-inferiorparietal | 0.095 | 0.570481 | 0.94582 |
| attention | rh-inferiortemporal | 0.157884 | 0.343787 | 0.853848 |
| attention | rh-isthmuscingulate | 0.139992 | 0.401873 | 0.888827 |
| attention | rh-lateraloccipital | 0.074537 | 0.656504 | 0.966672 |
| attention | rh-lateralorbitofrontal | 0.03393 | 0.839739 | 0.98315 |
| attention | rh-lingual | 0.11751 | 0.482293 | 0.908968 |
| attention | rh-medialorbitofrontal | 0.354772 | 0.028849 | 0.663823 |
| attention | rh-middletemporal | 0.144983 | 0.38513 | 0.884456 |
| attention | rh-parahippocampal | 0.003628 | 0.982753 | 0.995431 |
| attention | rh-paracentral | 0.084235 | 0.615097 | 0.966672 |
| attention | rh-parsopercularis | 0.113633 | 0.496958 | 0.914701 |
| attention | rh-parsorbitalis | 0.305556 | 0.062105 | 0.663823 |
| attention | rh-parstriangularis | 0.210504 | 0.204599 | 0.703819 |
| attention | rh-pericalcarine | -0.160561 | 0.335562 | 0.853848 |
| attention | rh-postcentral | -0.080234 | 0.632049 | 0.966672 |
| attention | rh-posteriorcingulate | -0.003263 | 0.984488 | 0.995431 |
| attention | rh-precentral | -0.00636 | 0.969768 | 0.992784 |
| attention | rh-precuneus | 0.244247 | 0.139452 | 0.663823 |
| attention | rh-rostralanteriorcingulate | -0.026032 | 0.876712 | 0.98315 |
| attention | rh-rostralmiddlefrontal | 0.212513 | 0.200211 | 0.6983 |
| attention | rh-superiorfrontal | 0.336424 | 0.038903 | 0.663823 |
| attention | rh-superiorparietal | 0.13916 | 0.404704 | 0.888827 |
| attention | rh-superiortemporal | -0.052041 | 0.756337 | 0.980556 |
| attention | rh-supramarginal | 0.045184 | 0.787648 | 0.98315 |
| attention | rh-frontalpole | 0.217837 | 0.188906 | 0.673573 |
| attention | rh-temporalpole | -0.029799 | 0.859038 | 0.98315 |
| attention | rh-transversetemporal | -0.065909 | 0.694212 | 0.972349 |
| attention | rh-insula | 0.052271 | 0.755292 | 0.980556 |
| attention | Left-Thalamus | 0.184179 | 0.268324 | 0.759409 |
| attention | Left-Caudate | 0.067053 | 0.689169 | 0.972349 |
| attention | Left-Putamen | 0.133373 | 0.424711 | 0.890771 |
| attention | Left-Pallidum | -0.042754 | 0.798828 | 0.98315 |
| attention | Left-Hippocampus | 0.14534 | 0.383948 | 0.884456 |
| attention | Left-Amygdala | 0.010511 | 0.95006 | 0.9844 |
| attention | Right-Thalamus | 0.204993 | 0.216975 | 0.712246 |
| attention | Right-Caudate | 0.071029 | 0.671738 | 0.969286 |
| attention | Right-Putamen | 0.079363 | 0.635762 | 0.966672 |
| attention | Right-Pallidum | -0.129133 | 0.439711 | 0.890771 |
| attention | Right-Hippocampus | 0.011627 | 0.944764 | 0.98315 |
| attention | Right-Amygdala | -0.099867 | 0.550808 | 0.936156 |
| language | lh-bankssts | 0.058029 | 0.729298 | 0.980556 |
| language | lh-caudalanteriorcingulate | 0.05214 | 0.755886 | 0.980556 |
| language | lh-caudalmiddlefrontal | 0.244133 | 0.139643 | 0.663823 |
| language | lh-cuneus | 0.117462 | 0.482473 | 0.908968 |
| language | lh-entorhinal | 0.028171 | 0.866668 | 0.98315 |
| language | lh-fusiform | 0.053282 | 0.750707 | 0.980556 |
| language | lh-inferiorparietal | -0.013519 | 0.935794 | 0.98315 |
| language | lh-inferiortemporal | 0.143858 | 0.388868 | 0.884726 |
| language | lh-isthmuscingulate | 0.109572 | 0.512556 | 0.914701 |
| language | lh-lateraloccipital | -0.070318 | 0.674843 | 0.97051 |
| language | lh-lateralorbitofrontal | 0.225709 | 0.173026 | 0.663823 |
| language | lh-lingual | -0.117345 | 0.482911 | 0.908968 |
| language | lh-medialorbitofrontal | 0.046909 | 0.779739 | 0.98315 |
| language | lh-middletemporal | 0.225624 | 0.173193 | 0.663823 |
| language | lh-parahippocampal | -0.138427 | 0.407207 | 0.888827 |
| language | lh-paracentral | 0.066461 | 0.691776 | 0.972349 |
| language | lh-parsopercularis | 0.206106 | 0.214436 | 0.712246 |
| language | lh-parsorbitalis | 0.066677 | 0.690825 | 0.972349 |
| language | lh-parstriangularis | 0.192461 | 0.247012 | 0.749137 |
| language | lh-pericalcarine | 0.088109 | 0.598872 | 0.953759 |
| language | lh-postcentral | 0.066716 | 0.69065 | 0.972349 |
| language | lh-posteriorcingulate | -0.049872 | 0.766202 | 0.980556 |
| language | lh-precentral | 0.249454 | 0.130952 | 0.663823 |
| language | lh-precuneus | 0.20277 | 0.222111 | 0.718104 |
| language | lh-rostralanteriorcingulate | 0.235338 | 0.154932 | 0.663823 |
| language | lh-rostralmiddlefrontal | -0.013154 | 0.937523 | 0.98315 |
| language | lh-superiorfrontal | 0.236811 | 0.15229 | 0.663823 |
| language | lh-superiorparietal | 0.241455 | 0.144175 | 0.663823 |
| language | lh-superiortemporal | 0.217979 | 0.188611 | 0.673573 |
| language | lh-supramarginal | -0.040479 | 0.809327 | 0.98315 |
| language | lh-frontalpole | 0.011758 | 0.944144 | 0.98315 |
| language | lh-temporalpole | 0.224006 | 0.176377 | 0.663823 |
| language | lh-transversetemporal | -0.128356 | 0.442493 | 0.890771 |
| language | lh-insula | 0.112764 | 0.500276 | 0.914701 |
| language | rh-bankssts | -0.090109 | 0.590571 | 0.947557 |
| language | rh-caudalanteriorcingulate | -0.232166 | 0.160734 | 0.663823 |
| language | rh-caudalmiddlefrontal | 0.030385 | 0.856298 | 0.98315 |
| language | rh-cuneus | -0.189803 | 0.253724 | 0.759409 |
| language | rh-entorhinal | -0.054857 | 0.743586 | 0.980556 |
| language | rh-fusiform | 0.039295 | 0.814808 | 0.98315 |
| language | rh-inferiorparietal | -0.184146 | 0.268411 | 0.759409 |
| language | rh-inferiortemporal | 0.160545 | 0.335611 | 0.853848 |
| language | rh-isthmuscingulate | 0.130254 | 0.435718 | 0.890771 |
| language | rh-lateraloccipital | -0.165902 | 0.31952 | 0.842904 |
| language | rh-lateralorbitofrontal | 0.100328 | 0.548961 | 0.936156 |
| language | rh-lingual | -0.117399 | 0.482709 | 0.908968 |
| language | rh-medialorbitofrontal | -0.059621 | 0.722165 | 0.980556 |
| language | rh-middletemporal | 0.09367 | 0.575913 | 0.94582 |
| language | rh-parahippocampal | -0.071704 | 0.668798 | 0.968294 |
| language | rh-paracentral | 0.021906 | 0.89614 | 0.98315 |
| language | rh-parsopercularis | 0.125681 | 0.452136 | 0.890771 |
| language | rh-parsorbitalis | 0.056317 | 0.736998 | 0.980556 |
| language | rh-parstriangularis | 0.259966 | 0.114969 | 0.663823 |
| language | rh-pericalcarine | 0.055981 | 0.738511 | 0.980556 |
| language | rh-postcentral | 0.095063 | 0.570225 | 0.94582 |
| language | rh-posteriorcingulate | -0.063692 | 0.704023 | 0.976548 |
| language | rh-precentral | 0.020255 | 0.903927 | 0.98315 |
| language | rh-precuneus | 0.05153 | 0.758656 | 0.980556 |
| language | rh-rostralanteriorcingulate | -0.241427 | 0.144224 | 0.663823 |
| language | rh-rostralmiddlefrontal | 0.077418 | 0.644089 | 0.966672 |
| language | rh-superiorfrontal | 0.32546 | 0.046162 | 0.663823 |
| language | rh-superiorparietal | -0.000961 | 0.995431 | 0.995431 |
| language | rh-superiortemporal | 0.014821 | 0.929624 | 0.98315 |
| language | rh-supramarginal | -0.225229 | 0.173967 | 0.663823 |
| language | rh-frontalpole | -0.23024 | 0.164331 | 0.663823 |
| language | rh-temporalpole | -0.02156 | 0.897767 | 0.98315 |
| language | rh-transversetemporal | -0.039026 | 0.816053 | 0.98315 |
| language | rh-insula | 0.055094 | 0.742513 | 0.980556 |
| language | Left-Thalamus | 0.250951 | 0.128579 | 0.663823 |
| language | Left-Caudate | 0.246266 | 0.13611 | 0.663823 |
| language | Left-Putamen | 0.238113 | 0.149982 | 0.663823 |
| language | Left-Pallidum | -0.04333 | 0.796173 | 0.98315 |
| language | Left-Hippocampus | 0.238362 | 0.149544 | 0.663823 |
| language | Left-Amygdala | 0.11616 | 0.487372 | 0.911174 |
| language | Right-Thalamus | 0.118059 | 0.480233 | 0.908968 |
| language | Right-Caudate | 0.193028 | 0.245595 | 0.749137 |
| language | Right-Putamen | 0.036754 | 0.826595 | 0.98315 |
| language | Right-Pallidum | -0.056904 | 0.734355 | 0.980556 |
| language | Right-Hippocampus | -0.01448 | 0.93124 | 0.98315 |
| language | Right-Amygdala | -0.142256 | 0.394226 | 0.886319 |
| language | Right-Accumbens-area | 0.019556 | 0.907228 | 0.98315 |
| language | Right-VentralDC | 0.075965 | 0.65034 | 0.966672 |
| language | Right-choroid-plexus | 0.157329 | 0.34551 | 0.853848 |
| executive fuction | lh-bankssts | 0.273631 | 0.096447 | 0.663823 |
| executive fuction | lh-caudalanteriorcingulate | 0.268356 | 0.103306 | 0.663823 |
| executive fuction | lh-caudalmiddlefrontal | 0.164582 | 0.323439 | 0.848041 |
| executive fuction | lh-cuneus | -0.091121 | 0.586388 | 0.947557 |
| executive fuction | lh-entorhinal | 0.236599 | 0.152669 | 0.663823 |
| executive fuction | lh-fusiform | 0.456901 | 0.003931 | 0.377601 |
| executive fuction | lh-inferiorparietal | 0.233441 | 0.158383 | 0.663823 |
| executive fuction | lh-inferiortemporal | 0.445339 | 0.005081 | 0.377601 |
| executive fuction | lh-isthmuscingulate | 0.36864 | 0.022762 | 0.652511 |
| executive fuction | lh-lateraloccipital | 0.25885 | 0.116594 | 0.663823 |
| executive fuction | lh-lateralorbitofrontal | 0.221883 | 0.180622 | 0.663823 |
| executive fuction | lh-lingual | 0.31296 | 0.05573 | 0.663823 |
| executive fuction | lh-medialorbitofrontal | 0.109483 | 0.512901 | 0.914701 |
| executive fuction | lh-middletemporal | 0.460738 | 0.003603 | 0.377601 |
| executive fuction | lh-parahippocampal | 0.223403 | 0.177576 | 0.663823 |
| executive fuction | lh-paracentral | 0.11002 | 0.510824 | 0.914701 |
| executive fuction | lh-parsopercularis | 0.107903 | 0.519039 | 0.914701 |
| executive fuction | lh-parsorbitalis | -0.022469 | 0.893484 | 0.98315 |
| executive fuction | lh-parstriangularis | 0.275816 | 0.093711 | 0.663823 |
| executive fuction | lh-pericalcarine | 0.188837 | 0.256192 | 0.759409 |
| executive fuction | lh-postcentral | -0.031203 | 0.852469 | 0.98315 |
| executive fuction | lh-posteriorcingulate | 0.260203 | 0.114627 | 0.663823 |
| executive fuction | lh-precentral | 0.109075 | 0.514482 | 0.914701 |
| executive fuction | lh-precuneus | 0.158571 | 0.341665 | 0.853848 |
| executive fuction | lh-rostralanteriorcingulate | 0.445077 | 0.00511 | 0.377601 |
| executive fuction | lh-rostralmiddlefrontal | 0.238053 | 0.150088 | 0.663823 |
| executive fuction | lh-superiorfrontal | 0.267014 | 0.105109 | 0.663823 |
| executive fuction | lh-superiorparietal | 0.073509 | 0.660952 | 0.966672 |
| executive fuction | lh-superiortemporal | 0.369258 | 0.022518 | 0.652511 |
| executive fuction | lh-supramarginal | 0.00589 | 0.972005 | 0.992784 |
| executive fuction | lh-frontalpole | 0.127049 | 0.447191 | 0.890771 |
| executive fuction | lh-temporalpole | 0.452606 | 0.004329 | 0.377601 |
| executive fuction | lh-transversetemporal | 0.105137 | 0.529872 | 0.917991 |
| executive fuction | lh-insula | 0.332718 | 0.041245 | 0.663823 |
| executive fuction | rh-bankssts | 0.285115 | 0.082728 | 0.663823 |
| executive fuction | rh-caudalanteriorcingulate | 0.042793 | 0.798645 | 0.98315 |
| executive fuction | rh-caudalmiddlefrontal | 0.225627 | 0.173188 | 0.663823 |
| executive fuction | rh-cuneus | -0.13915 | 0.404738 | 0.888827 |
| executive fuction | rh-entorhinal | 0.264368 | 0.108732 | 0.663823 |
| executive fuction | rh-fusiform | 0.288771 | 0.078694 | 0.663823 |
| executive fuction | rh-inferiorparietal | 0.146167 | 0.381219 | 0.884456 |
| executive fuction | rh-inferiortemporal | 0.420307 | 0.008606 | 0.528635 |
| executive fuction | rh-isthmuscingulate | 0.399843 | 0.012876 | 0.652511 |
| executive fuction | rh-lateraloccipital | 0.149527 | 0.370249 | 0.884456 |
| executive fuction | rh-lateralorbitofrontal | 0.091112 | 0.586426 | 0.947557 |
| executive fuction | rh-lingual | 0.23855 | 0.149213 | 0.663823 |
| executive fuction | rh-medialorbitofrontal | 0.285186 | 0.082649 | 0.663823 |
| executive fuction | rh-middletemporal | 0.443675 | 0.005269 | 0.377601 |
| executive fuction | rh-parahippocampal | 0.235017 | 0.155512 | 0.663823 |
| executive fuction | rh-paracentral | 0.135807 | 0.416228 | 0.890771 |
| executive fuction | rh-parsopercularis | 0.166074 | 0.31901 | 0.842904 |
| executive fuction | rh-parsorbitalis | 0.088506 | 0.59722 | 0.953759 |
| executive fuction | rh-parstriangularis | 0.355764 | 0.028373 | 0.663823 |
| executive fuction | rh-pericalcarine | -0.009669 | 0.954055 | 0.986162 |
| executive fuction | rh-postcentral | 0.072478 | 0.66543 | 0.966672 |
| executive fuction | rh-posteriorcingulate | 0.035242 | 0.833626 | 0.98315 |
| executive fuction | rh-precentral | 0.060724 | 0.717234 | 0.980556 |
| executive fuction | rh-precuneus | 0.226891 | 0.170728 | 0.663823 |
| executive fuction | rh-rostralanteriorcingulate | -0.067943 | 0.685254 | 0.972349 |
| executive fuction | rh-rostralmiddlefrontal | 0.1966 | 0.236798 | 0.748699 |
| executive fuction | rh-superiorfrontal | 0.244819 | 0.1385 | 0.663823 |
| executive fuction | rh-superiorparietal | -0.064412 | 0.700831 | 0.976548 |
| executive fuction | rh-superiortemporal | 0.244655 | 0.138773 | 0.663823 |
| executive fuction | rh-supramarginal | -0.02324 | 0.889848 | 0.98315 |
| executive fuction | rh-frontalpole | -0.073825 | 0.659584 | 0.966672 |
| executive fuction | rh-temporalpole | 0.086591 | 0.60521 | 0.960297 |
| executive fuction | rh-transversetemporal | -0.028308 | 0.866027 | 0.98315 |
| executive fuction | rh-insula | 0.281933 | 0.086367 | 0.663823 |
| executive fuction | Left-Thalamus | 0.330347 | 0.042803 | 0.663823 |
| executive fuction | Left-Caudate | 0.271744 | 0.09886 | 0.663823 |
| executive fuction | Left-Putamen | 0.317116 | 0.052388 | 0.663823 |
| executive fuction | Left-Pallidum | 0.114568 | 0.493398 | 0.914701 |
| executive fuction | Left-Hippocampus | 0.314205 | 0.054712 | 0.663823 |
| executive fuction | Left-Amygdala | 0.25743 | 0.118684 | 0.663823 |
| executive fuction | Right-Thalamus | 0.147397 | 0.377183 | 0.884456 |
| executive fuction | Right-Caudate | 0.184134 | 0.268442 | 0.759409 |
| executive fuction | Right-Putamen | 0.231858 | 0.161305 | 0.663823 |
| executive fuction | Right-Pallidum | 0.13141 | 0.43162 | 0.890771 |
| executive fuction | Right-Hippocampus | 0.149653 | 0.369842 | 0.884456 |
| executive fuction | Right-Amygdala | 0.13127 | 0.432113 | 0.890771 |

### Table S19. Correlation between Cognitive Domains and Regional FDG Metabolism in non-AD group

| **Cognitive domain** | **Brain region** | **Partial r** | **P** | **FDR-P** |
| --- | --- | --- | --- | --- |
| visuospatial | Left-Thalamus | 0.217802 | 0.188978 | 0.560837 |
| visuospatial | Left-Caudate | 0.245904 | 0.136705 | 0.509451 |
| visuospatial | Left-Putamen | 0.160097 | 0.33698 | 0.635291 |
| visuospatial | Left-Pallidum | 0.199585 | 0.229613 | 0.563903 |
| visuospatial | Left-Hippocampus | 0.273065 | 0.097167 | 0.493425 |
| visuospatial | Left-Amygdala | 0.226637 | 0.171219 | 0.535786 |
| visuospatial | Right-Thalamus | 0.290162 | 0.077201 | 0.480893 |
| visuospatial | Right-Caudate | 0.149975 | 0.368799 | 0.663433 |
| visuospatial | Right-Putamen | 0.241618 | 0.143897 | 0.509451 |
| visuospatial | Right-Pallidum | 0.149401 | 0.370657 | 0.663433 |
| visuospatial | Right-Hippocampus | 0.122624 | 0.463298 | 0.737433 |
| visuospatial | Right-Amygdala | 0.068436 | 0.683088 | 0.858757 |
| visuospatial | lh-bankssts | 0.140852 | 0.398959 | 0.684864 |
| visuospatial | lh-caudalanteriorcingulate | 0.362674 | 0.025234 | 0.451068 |
| visuospatial | lh-caudalmiddlefrontal | 0.238224 | 0.149786 | 0.509451 |
| visuospatial | lh-cuneus | 0.302776 | 0.064642 | 0.451068 |
| visuospatial | lh-entorhinal | 0.189815 | 0.253692 | 0.569261 |
| visuospatial | lh-fusiform | -0.02783 | 0.868271 | 0.948999 |
| visuospatial | lh-inferiorparietal | 0.309676 | 0.05849 | 0.451068 |
| visuospatial | lh-inferiortemporal | 0.078124 | 0.641063 | 0.84014 |
| visuospatial | lh-isthmuscingulate | 0.218613 | 0.187296 | 0.560837 |
| visuospatial | lh-lateraloccipital | 0.314191 | 0.054723 | 0.451068 |
| visuospatial | lh-lateralorbitofrontal | 0.344891 | 0.033957 | 0.451068 |
| visuospatial | lh-lingual | 0.074961 | 0.654668 | 0.849241 |
| visuospatial | lh-medialorbitofrontal | 0.275494 | 0.094111 | 0.493425 |
| visuospatial | lh-middletemporal | 0.288506 | 0.078981 | 0.480893 |
| visuospatial | lh-parahippocampal | -0.015122 | 0.928202 | 0.960609 |
| visuospatial | lh-paracentral | 0.175663 | 0.291463 | 0.614871 |
| visuospatial | lh-parsopercularis | 0.410576 | 0.010455 | 0.451068 |
| visuospatial | lh-parsorbitalis | 0.371991 | 0.021464 | 0.451068 |
| visuospatial | lh-parstriangularis | 0.232831 | 0.159505 | 0.509451 |
| visuospatial | lh-pericalcarine | 0.196976 | 0.235884 | 0.563903 |
| visuospatial | lh-postcentral | 0.23462 | 0.156232 | 0.509451 |
| visuospatial | lh-posteriorcingulate | 0.15446 | 0.354486 | 0.657515 |
| visuospatial | lh-precentral | 0.412004 | 0.010164 | 0.451068 |
| visuospatial | lh-precuneus | 0.28429 | 0.08366 | 0.480893 |
| visuospatial | lh-rostralanteriorcingulate | 0.2121 | 0.201109 | 0.561545 |
| visuospatial | lh-rostralmiddlefrontal | 0.353215 | 0.029609 | 0.451068 |
| visuospatial | lh-superiorfrontal | 0.351105 | 0.030665 | 0.451068 |
| visuospatial | lh-superiorparietal | 0.10053 | 0.548151 | 0.785669 |
| visuospatial | lh-superiortemporal | 0.25129 | 0.128047 | 0.509451 |
| visuospatial | lh-supramarginal | 0.313955 | 0.054915 | 0.451068 |
| visuospatial | lh-frontalpole | 0.337543 | 0.038218 | 0.451068 |
| visuospatial | lh-temporalpole | -0.027772 | 0.86854 | 0.948999 |
| visuospatial | lh-transversetemporal | 0.409036 | 0.010776 | 0.451068 |
| visuospatial | lh-insula | -0.049248 | 0.769046 | 0.909499 |
| visuospatial | rh-bankssts | 0.197777 | 0.233946 | 0.563903 |
| visuospatial | rh-caudalanteriorcingulate | 0.179096 | 0.281984 | 0.614148 |
| visuospatial | rh-caudalmiddlefrontal | 0.21518 | 0.194491 | 0.561545 |
| visuospatial | rh-cuneus | 0.394925 | 0.014135 | 0.451068 |
| visuospatial | rh-entorhinal | 0.046102 | 0.783437 | 0.911894 |
| visuospatial | rh-fusiform | -0.020309 | 0.903671 | 0.95376 |
| visuospatial | rh-inferiorparietal | 0.330838 | 0.042476 | 0.451068 |
| visuospatial | rh-inferiortemporal | 0.053279 | 0.750721 | 0.896966 |
| visuospatial | rh-isthmuscingulate | 0.232245 | 0.160588 | 0.509451 |
| visuospatial | rh-lateraloccipital | 0.316385 | 0.052964 | 0.451068 |
| visuospatial | rh-lateralorbitofrontal | 0.312123 | 0.056424 | 0.451068 |
| visuospatial | rh-lingual | 0.074794 | 0.655392 | 0.849241 |
| visuospatial | rh-medialorbitofrontal | 0.321191 | 0.049268 | 0.451068 |
| visuospatial | rh-middletemporal | 0.292916 | 0.074308 | 0.480893 |
| visuospatial | rh-parahippocampal | 0.000228 | 0.998915 | 0.998915 |
| visuospatial | rh-paracentral | 0.176044 | 0.290401 | 0.614871 |
| visuospatial | rh-parsopercularis | 0.348705 | 0.031904 | 0.451068 |
| visuospatial | rh-parsorbitalis | 0.330861 | 0.042461 | 0.451068 |
| visuospatial | rh-parstriangularis | 0.266818 | 0.105374 | 0.506526 |
| visuospatial | rh-pericalcarine | 0.283953 | 0.084043 | 0.480893 |
| visuospatial | rh-postcentral | 0.194698 | 0.241456 | 0.563903 |
| visuospatial | rh-posteriorcingulate | 0.122966 | 0.462041 | 0.737433 |
| visuospatial | rh-precentral | 0.305468 | 0.062183 | 0.451068 |
| visuospatial | rh-precuneus | 0.2456 | 0.137206 | 0.509451 |
| visuospatial | rh-rostralanteriorcingulate | 0.132221 | 0.428757 | 0.706911 |
| visuospatial | rh-rostralmiddlefrontal | 0.349157 | 0.031667 | 0.451068 |
| visuospatial | rh-superiorfrontal | 0.268894 | 0.10259 | 0.504721 |
| visuospatial | rh-superiorparietal | 0.150416 | 0.367378 | 0.663433 |
| visuospatial | rh-superiortemporal | 0.20256 | 0.222601 | 0.563903 |
| visuospatial | rh-supramarginal | 0.327613 | 0.044657 | 0.451068 |
| visuospatial | rh-frontalpole | 0.378412 | 0.019149 | 0.451068 |
| visuospatial | rh-temporalpole | -0.083777 | 0.617028 | 0.822705 |
| visuospatial | rh-transversetemporal | 0.359899 | 0.026459 | 0.451068 |
| visuospatial | rh-insula | -0.052172 | 0.755743 | 0.898299 |
| memory | Left-Thalamus | 0.317904 | 0.051773 | 0.451068 |
| memory | Left-Caudate | 0.085282 | 0.610696 | 0.8212 |
| memory | Left-Putamen | 0.06823 | 0.683989 | 0.858757 |
| memory | Left-Pallidum | 0.235479 | 0.154677 | 0.509451 |
| memory | Left-Hippocampus | 0.077365 | 0.644315 | 0.842002 |
| memory | Left-Amygdala | -0.047515 | 0.776964 | 0.91119 |
| memory | Right-Thalamus | 0.311403 | 0.057026 | 0.451068 |
| memory | Right-Caudate | 0.039087 | 0.815769 | 0.935795 |
| memory | Right-Putamen | 0.178592 | 0.283365 | 0.614148 |
| memory | Right-Pallidum | 0.199524 | 0.229758 | 0.563903 |
| memory | Right-Hippocampus | 0.100356 | 0.548849 | 0.785669 |
| memory | Right-Amygdala | -0.01485 | 0.929488 | 0.960609 |
| memory | lh-bankssts | 0.0873 | 0.602245 | 0.8212 |
| memory | lh-caudalanteriorcingulate | 0.24026 | 0.146233 | 0.509451 |
| memory | lh-caudalmiddlefrontal | 0.153638 | 0.357084 | 0.659673 |
| memory | lh-cuneus | 0.444798 | 0.005142 | 0.451068 |
| memory | lh-entorhinal | -0.044392 | 0.791287 | 0.914553 |
| memory | lh-fusiform | 0.121625 | 0.466975 | 0.738173 |
| memory | lh-inferiorparietal | 0.137185 | 0.41147 | 0.698436 |
| memory | lh-inferiortemporal | 0.079031 | 0.637181 | 0.83989 |
| memory | lh-isthmuscingulate | 0.11452 | 0.493583 | 0.749334 |
| memory | lh-lateraloccipital | 0.152549 | 0.360543 | 0.663399 |
| memory | lh-lateralorbitofrontal | 0.206486 | 0.213573 | 0.563903 |
| memory | lh-lingual | 0.300605 | 0.06668 | 0.451068 |
| memory | lh-medialorbitofrontal | 0.263364 | 0.110132 | 0.506609 |
| memory | lh-middletemporal | 0.168768 | 0.311111 | 0.617813 |
| memory | lh-parahippocampal | -0.02211 | 0.895175 | 0.95376 |
| memory | lh-paracentral | 0.297454 | 0.069728 | 0.464851 |
| memory | lh-parsopercularis | 0.234365 | 0.156694 | 0.509451 |
| memory | lh-parsorbitalis | 0.133833 | 0.4231 | 0.704225 |
| memory | lh-parstriangularis | 0.220788 | 0.182836 | 0.560698 |
| memory | lh-pericalcarine | 0.248422 | 0.132605 | 0.509451 |
| memory | lh-postcentral | 0.353885 | 0.029279 | 0.451068 |
| memory | lh-posteriorcingulate | 0.332319 | 0.041504 | 0.451068 |
| memory | lh-precentral | 0.357938 | 0.027352 | 0.451068 |
| memory | lh-precuneus | 0.143327 | 0.390642 | 0.683251 |
| memory | lh-rostralanteriorcingulate | 0.220951 | 0.182506 | 0.560698 |
| memory | lh-rostralmiddlefrontal | 0.283911 | 0.08409 | 0.480893 |
| memory | lh-superiorfrontal | 0.141191 | 0.397813 | 0.684864 |
| memory | lh-superiorparietal | 0.241126 | 0.14474 | 0.509451 |
| memory | lh-superiortemporal | 0.256147 | 0.120596 | 0.509451 |
| memory | lh-supramarginal | 0.266056 | 0.10641 | 0.506526 |
| memory | lh-frontalpole | 0.151663 | 0.363373 | 0.663433 |
| memory | lh-temporalpole | 0.031213 | 0.852424 | 0.948999 |
| memory | lh-transversetemporal | 0.084557 | 0.613745 | 0.8212 |
| memory | lh-insula | 0.159453 | 0.338952 | 0.6364 |
| memory | rh-bankssts | 0.235268 | 0.155058 | 0.509451 |
| memory | rh-caudalanteriorcingulate | 0.111116 | 0.506599 | 0.76405 |
| memory | rh-caudalmiddlefrontal | 0.168692 | 0.311332 | 0.617813 |
| memory | rh-cuneus | 0.499109 | 0.001424 | 0.39074 |
| memory | rh-entorhinal | 0.018619 | 0.911653 | 0.955263 |
| memory | rh-fusiform | 0.09965 | 0.551676 | 0.785669 |
| memory | rh-inferiorparietal | 0.16713 | 0.315899 | 0.618356 |
| memory | rh-inferiortemporal | 0.106001 | 0.526474 | 0.773732 |
| memory | rh-isthmuscingulate | 0.119354 | 0.475396 | 0.743817 |
| memory | rh-lateraloccipital | 0.16423 | 0.324489 | 0.621937 |
| memory | rh-lateralorbitofrontal | 0.168926 | 0.310652 | 0.617813 |
| memory | rh-lingual | 0.334258 | 0.040258 | 0.451068 |
| memory | rh-medialorbitofrontal | 0.247541 | 0.134028 | 0.509451 |
| memory | rh-middletemporal | 0.237801 | 0.150533 | 0.509451 |
| memory | rh-parahippocampal | 0.131589 | 0.430986 | 0.708049 |
| memory | rh-paracentral | 0.280209 | 0.088391 | 0.480893 |
| memory | rh-parsopercularis | 0.16538 | 0.321067 | 0.621937 |
| memory | rh-parsorbitalis | 0.026075 | 0.876512 | 0.953181 |
| memory | rh-parstriangularis | 0.219229 | 0.186025 | 0.560837 |
| memory | rh-pericalcarine | 0.189134 | 0.255432 | 0.570382 |
| memory | rh-postcentral | 0.306364 | 0.061382 | 0.451068 |
| memory | rh-posteriorcingulate | 0.375801 | 0.020063 | 0.451068 |
| memory | rh-precentral | 0.328028 | 0.044371 | 0.451068 |
| memory | rh-precuneus | 0.130753 | 0.433946 | 0.709137 |
| memory | rh-rostralanteriorcingulate | 0.255165 | 0.122076 | 0.509451 |
| memory | rh-rostralmiddlefrontal | 0.258739 | 0.116756 | 0.509451 |
| memory | rh-superiorfrontal | 0.098749 | 0.5553 | 0.785798 |
| memory | rh-superiorparietal | 0.214548 | 0.195836 | 0.561545 |
| memory | rh-superiortemporal | 0.244676 | 0.138737 | 0.509451 |
| memory | rh-supramarginal | 0.324236 | 0.047036 | 0.451068 |
| memory | rh-frontalpole | 0.242358 | 0.142636 | 0.509451 |
| memory | rh-temporalpole | 0.072405 | 0.665747 | 0.856375 |
| memory | rh-transversetemporal | 0.093903 | 0.574959 | 0.803894 |
| memory | rh-insula | 0.215735 | 0.193314 | 0.561545 |
| attention | Left-Thalamus | -0.357043 | 0.027769 | 0.451068 |
| attention | Left-Caudate | -0.064964 | 0.698387 | 0.865668 |
| attention | Left-Putamen | -0.140838 | 0.399008 | 0.684864 |
| attention | Left-Pallidum | -0.186242 | 0.262905 | 0.582924 |
| attention | Left-Hippocampus | 0.008413 | 0.960021 | 0.977013 |
| attention | Left-Amygdala | 0.065232 | 0.697201 | 0.865668 |
| attention | Right-Thalamus | -0.346392 | 0.033137 | 0.451068 |
| attention | Right-Caudate | -0.177253 | 0.287047 | 0.614148 |
| attention | Right-Putamen | -0.147245 | 0.377678 | 0.67078 |
| attention | Right-Pallidum | -0.344775 | 0.034021 | 0.451068 |
| attention | Right-Hippocampus | -0.210838 | 0.203865 | 0.561545 |
| attention | Right-Amygdala | -0.241854 | 0.143494 | 0.509451 |
| attention | lh-bankssts | -0.031926 | 0.849092 | 0.948999 |
| attention | lh-caudalanteriorcingulate | -0.106734 | 0.523604 | 0.77198 |
| attention | lh-caudalmiddlefrontal | -0.122498 | 0.463758 | 0.737433 |
| attention | lh-cuneus | -0.024751 | 0.882735 | 0.95376 |
| attention | lh-entorhinal | 0.235695 | 0.154289 | 0.509451 |
| attention | lh-fusiform | -0.174948 | 0.293462 | 0.614871 |
| attention | lh-inferiorparietal | 0.160521 | 0.335686 | 0.635291 |
| attention | lh-inferiortemporal | -0.095555 | 0.568221 | 0.796896 |
| attention | lh-isthmuscingulate | -0.012396 | 0.94112 | 0.964176 |
| attention | lh-lateraloccipital | 0.237854 | 0.150439 | 0.509451 |
| attention | lh-lateralorbitofrontal | -0.030266 | 0.856855 | 0.948999 |
| attention | lh-lingual | -0.158514 | 0.341843 | 0.639219 |
| attention | lh-medialorbitofrontal | 0.049232 | 0.76912 | 0.909499 |
| attention | lh-middletemporal | 0.123108 | 0.461519 | 0.737433 |
| attention | lh-parahippocampal | -0.023029 | 0.890842 | 0.95376 |
| attention | lh-paracentral | -0.198563 | 0.232055 | 0.563903 |
| attention | lh-parsopercularis | 0.09874 | 0.555334 | 0.785798 |
| attention | lh-parsorbitalis | 0.173249 | 0.298249 | 0.615221 |
| attention | lh-parstriangularis | -0.055782 | 0.739408 | 0.895073 |
| attention | lh-pericalcarine | -0.061257 | 0.714855 | 0.872394 |
| attention | lh-postcentral | -0.0997 | 0.551477 | 0.785669 |
| attention | lh-posteriorcingulate | -0.196105 | 0.238005 | 0.563903 |
| attention | lh-precentral | -0.005598 | 0.973392 | 0.984089 |
| attention | lh-precuneus | 0.045756 | 0.785022 | 0.911894 |
| attention | lh-rostralanteriorcingulate | -0.117479 | 0.482407 | 0.745428 |
| attention | lh-rostralmiddlefrontal | 0.024267 | 0.885012 | 0.95376 |
| attention | lh-superiorfrontal | 0.168937 | 0.31062 | 0.617813 |
| attention | lh-superiorparietal | -0.116849 | 0.484774 | 0.745428 |
| attention | lh-superiortemporal | -0.007646 | 0.963661 | 0.978552 |
| attention | lh-supramarginal | 0.086483 | 0.605659 | 0.8212 |
| attention | lh-frontalpole | 0.161906 | 0.331478 | 0.630082 |
| attention | lh-temporalpole | -0.128099 | 0.443414 | 0.718206 |
| attention | lh-transversetemporal | 0.250083 | 0.129951 | 0.509451 |
| attention | lh-insula | -0.220075 | 0.184291 | 0.560837 |
| attention | rh-bankssts | -0.120705 | 0.470377 | 0.741005 |
| attention | rh-caudalanteriorcingulate | -0.096059 | 0.566175 | 0.796454 |
| attention | rh-caudalmiddlefrontal | -0.130531 | 0.434732 | 0.709137 |
| attention | rh-cuneus | -0.02052 | 0.902676 | 0.95376 |
| attention | rh-entorhinal | -0.147573 | 0.376606 | 0.67078 |
| attention | rh-fusiform | -0.239368 | 0.147782 | 0.509451 |
| attention | rh-inferiorparietal | 0.116484 | 0.486149 | 0.745428 |
| attention | rh-inferiortemporal | -0.199116 | 0.230731 | 0.563903 |
| attention | rh-isthmuscingulate | 0.035741 | 0.831304 | 0.944197 |
| attention | rh-lateraloccipital | 0.235983 | 0.153772 | 0.509451 |
| attention | rh-lateralorbitofrontal | -0.068937 | 0.680887 | 0.858757 |
| attention | rh-lingual | -0.202662 | 0.222362 | 0.563903 |
| attention | rh-medialorbitofrontal | 0.020095 | 0.904685 | 0.95376 |
| attention | rh-middletemporal | -0.032388 | 0.846931 | 0.948999 |
| attention | rh-parahippocampal | -0.034875 | 0.835334 | 0.946438 |
| attention | rh-paracentral | -0.236176 | 0.153424 | 0.509451 |
| attention | rh-parsopercularis | 0.032552 | 0.846168 | 0.948999 |
| attention | rh-parsorbitalis | 0.174014 | 0.296087 | 0.614871 |
| attention | rh-parstriangularis | -0.038251 | 0.819647 | 0.937128 |
| attention | rh-pericalcarine | -0.066337 | 0.692323 | 0.865404 |
| attention | rh-postcentral | -0.137584 | 0.410098 | 0.698436 |
| attention | rh-posteriorcingulate | -0.2344 | 0.156631 | 0.509451 |
| attention | rh-precentral | -0.088335 | 0.597934 | 0.8212 |
| attention | rh-precuneus | -0.014283 | 0.932177 | 0.960609 |
| attention | rh-rostralanteriorcingulate | -0.237809 | 0.150519 | 0.509451 |
| attention | rh-rostralmiddlefrontal | -0.009953 | 0.95271 | 0.973881 |
| attention | rh-superiorfrontal | 0.070171 | 0.675486 | 0.858757 |
| attention | rh-superiorparietal | -0.108157 | 0.518052 | 0.771887 |
| attention | rh-superiortemporal | -0.113525 | 0.49737 | 0.752599 |
| attention | rh-supramarginal | 0.000693 | 0.996704 | 0.998876 |
| attention | rh-frontalpole | 0.146751 | 0.3793 | 0.67107 |
| attention | rh-temporalpole | -0.217916 | 0.188741 | 0.560837 |
| attention | rh-transversetemporal | 0.201939 | 0.224051 | 0.563903 |
| attention | rh-insula | -0.167832 | 0.313844 | 0.617813 |
| language | Left-Thalamus | 0.185496 | 0.264856 | 0.582937 |
| language | Left-Caudate | 0.193315 | 0.244881 | 0.563903 |
| language | Left-Putamen | 0.244253 | 0.139442 | 0.509451 |
| language | Left-Pallidum | 0.242543 | 0.142322 | 0.509451 |
| language | Left-Hippocampus | 0.247167 | 0.134637 | 0.509451 |
| language | Left-Amygdala | 0.285798 | 0.081963 | 0.480893 |
| language | Right-Thalamus | 0.176556 | 0.288979 | 0.614871 |
| language | Right-Caudate | 0.019362 | 0.908145 | 0.95376 |
| language | Right-Putamen | 0.23327 | 0.158697 | 0.509451 |
| language | Right-Pallidum | 0.119424 | 0.475134 | 0.743817 |
| language | Right-Hippocampus | 0.004605 | 0.978109 | 0.986689 |
| language | Right-Amygdala | 0.005766 | 0.972593 | 0.984089 |
| language | lh-bankssts | 0.046772 | 0.780365 | 0.91119 |
| language | lh-caudalanteriorcingulate | 0.301153 | 0.066161 | 0.451068 |
| language | lh-caudalmiddlefrontal | 0.150662 | 0.366586 | 0.663433 |
| language | lh-cuneus | 0.20157 | 0.224916 | 0.563903 |
| language | lh-entorhinal | 0.196333 | 0.237449 | 0.563903 |
| language | lh-fusiform | -0.115838 | 0.488588 | 0.745917 |
| language | lh-inferiorparietal | 0.21164 | 0.20211 | 0.561545 |
| language | lh-inferiortemporal | -0.033457 | 0.841944 | 0.948999 |
| language | lh-isthmuscingulate | 0.070202 | 0.675351 | 0.858757 |
| language | lh-lateraloccipital | 0.211951 | 0.201433 | 0.561545 |
| language | lh-lateralorbitofrontal | 0.28095 | 0.087517 | 0.480893 |
| language | lh-lingual | 0.069141 | 0.679997 | 0.858757 |
| language | lh-medialorbitofrontal | 0.174467 | 0.294813 | 0.614871 |
| language | lh-middletemporal | 0.134871 | 0.419477 | 0.704225 |
| language | lh-parahippocampal | -0.087351 | 0.602034 | 0.8212 |
| language | lh-paracentral | 0.169085 | 0.310191 | 0.617813 |
| language | lh-parsopercularis | 0.317279 | 0.05226 | 0.451068 |
| language | lh-parsorbitalis | 0.305013 | 0.062594 | 0.451068 |
| language | lh-parstriangularis | 0.190785 | 0.25123 | 0.569261 |
| language | lh-pericalcarine | 0.177296 | 0.286931 | 0.614148 |
| language | lh-postcentral | 0.107609 | 0.520185 | 0.771887 |
| language | lh-posteriorcingulate | 0.143608 | 0.389701 | 0.683251 |
| language | lh-precentral | 0.325126 | 0.046399 | 0.451068 |
| language | lh-precuneus | 0.167917 | 0.313595 | 0.617813 |
| language | lh-rostralanteriorcingulate | 0.193021 | 0.245612 | 0.563903 |
| language | lh-rostralmiddlefrontal | 0.264453 | 0.108615 | 0.506526 |
| language | lh-superiorfrontal | 0.24235 | 0.142649 | 0.509451 |
| language | lh-superiorparietal | 0.021605 | 0.897557 | 0.95376 |
| language | lh-superiortemporal | 0.076803 | 0.646731 | 0.842766 |
| language | lh-supramarginal | 0.200219 | 0.228106 | 0.563903 |
| language | lh-frontalpole | 0.234129 | 0.157124 | 0.509451 |
| language | lh-temporalpole | -0.078641 | 0.638847 | 0.83989 |
| language | lh-transversetemporal | 0.36265 | 0.025245 | 0.451068 |
| language | lh-insula | -0.078595 | 0.639047 | 0.83989 |
| language | rh-bankssts | 0.134476 | 0.420855 | 0.704225 |
| language | rh-caudalanteriorcingulate | 0.084614 | 0.613502 | 0.8212 |
| language | rh-caudalmiddlefrontal | 0.115541 | 0.489711 | 0.745917 |
| language | rh-cuneus | 0.304505 | 0.063055 | 0.451068 |
| language | rh-entorhinal | -0.016415 | 0.922079 | 0.957464 |
| language | rh-fusiform | -0.133557 | 0.424066 | 0.704225 |
| language | rh-inferiorparietal | 0.190482 | 0.251997 | 0.569261 |
| language | rh-inferiortemporal | -0.086659 | 0.604924 | 0.8212 |
| language | rh-isthmuscingulate | 0.056804 | 0.734807 | 0.89421 |
| language | rh-lateraloccipital | 0.177555 | 0.286214 | 0.614148 |
| language | rh-lateralorbitofrontal | 0.165267 | 0.3214 | 0.621937 |
| language | rh-lingual | 0.027923 | 0.867835 | 0.948999 |
| language | rh-medialorbitofrontal | 0.167683 | 0.314279 | 0.617813 |
| language | rh-middletemporal | 0.098353 | 0.556891 | 0.785798 |
| language | rh-parahippocampal | -0.173782 | 0.296742 | 0.614871 |
| language | rh-paracentral | 0.065371 | 0.696587 | 0.865668 |
| language | rh-parsopercularis | 0.192705 | 0.246401 | 0.563903 |
| language | rh-parsorbitalis | 0.211772 | 0.201823 | 0.561545 |
| language | rh-parstriangularis | 0.164476 | 0.323755 | 0.621937 |
| language | rh-pericalcarine | 0.20902 | 0.207881 | 0.563903 |
| language | rh-postcentral | 0.103473 | 0.536439 | 0.779687 |
| language | rh-posteriorcingulate | 0.055032 | 0.742792 | 0.896809 |
| language | rh-precentral | 0.208786 | 0.208404 | 0.563903 |
| language | rh-precuneus | 0.109454 | 0.513014 | 0.768685 |
| language | rh-rostralanteriorcingulate | 0.072236 | 0.666483 | 0.856375 |
| language | rh-rostralmiddlefrontal | 0.205309 | 0.216251 | 0.563903 |
| language | rh-superiorfrontal | 0.142057 | 0.394897 | 0.684864 |
| language | rh-superiorparietal | 0.061228 | 0.714984 | 0.872394 |
| language | rh-superiortemporal | 0.026393 | 0.875018 | 0.953181 |
| language | rh-supramarginal | 0.233665 | 0.157972 | 0.509451 |
| language | rh-frontalpole | 0.213978 | 0.197054 | 0.561545 |
| language | rh-temporalpole | -0.185982 | 0.263583 | 0.582924 |
| language | rh-transversetemporal | 0.287521 | 0.080055 | 0.480893 |
| language | rh-insula | -0.138239 | 0.407851 | 0.69744 |
| executive fuction | Left-Thalamus | -0.053308 | 0.750591 | 0.896966 |
| executive fuction | Left-Caudate | 0.093216 | 0.577773 | 0.80538 |
| executive fuction | Left-Putamen | 0.03119 | 0.85253 | 0.948999 |
| executive fuction | Left-Pallidum | -0.017829 | 0.915388 | 0.956492 |
| executive fuction | Left-Hippocampus | 0.38304 | 0.017613 | 0.451068 |
| executive fuction | Left-Amygdala | 0.432703 | 0.006662 | 0.451068 |
| executive fuction | Right-Thalamus | 0.036717 | 0.826767 | 0.941368 |
| executive fuction | Right-Caudate | 0.063213 | 0.70615 | 0.868527 |
| executive fuction | Right-Putamen | 0.053735 | 0.748659 | 0.896966 |
| executive fuction | Right-Pallidum | -0.080074 | 0.632728 | 0.838935 |
| executive fuction | Right-Hippocampus | 0.171267 | 0.303896 | 0.617813 |
| executive fuction | Right-Amygdala | 0.253839 | 0.124096 | 0.509451 |
| executive fuction | lh-bankssts | 0.274123 | 0.095827 | 0.493425 |
| executive fuction | lh-caudalanteriorcingulate | 0.149501 | 0.370331 | 0.663433 |
| executive fuction | lh-caudalmiddlefrontal | 0.122187 | 0.464904 | 0.737433 |
| executive fuction | lh-cuneus | 0.086471 | 0.605711 | 0.8212 |
| executive fuction | lh-entorhinal | 0.492134 | 0.001699 | 0.39074 |
| executive fuction | lh-fusiform | -0.028325 | 0.865947 | 0.948999 |
| executive fuction | lh-inferiorparietal | 0.342345 | 0.035387 | 0.451068 |
| executive fuction | lh-inferiortemporal | 0.132708 | 0.427044 | 0.70662 |
| executive fuction | lh-isthmuscingulate | 0.282402 | 0.085824 | 0.480893 |
| executive fuction | lh-lateraloccipital | 0.283674 | 0.084361 | 0.480893 |
| executive fuction | lh-lateralorbitofrontal | 0.195799 | 0.238752 | 0.563903 |
| executive fuction | lh-lingual | -0.047385 | 0.777557 | 0.91119 |
| executive fuction | lh-medialorbitofrontal | 0.192765 | 0.246251 | 0.563903 |
| executive fuction | lh-middletemporal | 0.371338 | 0.021712 | 0.451068 |
| executive fuction | lh-parahippocampal | 0.128179 | 0.443126 | 0.718206 |
| executive fuction | lh-paracentral | 0.02238 | 0.893901 | 0.95376 |
| executive fuction | lh-parsopercularis | 0.382634 | 0.017743 | 0.451068 |
| executive fuction | lh-parsorbitalis | 0.327618 | 0.044654 | 0.451068 |
| executive fuction | lh-parstriangularis | 0.155967 | 0.349752 | 0.65136 |
| executive fuction | lh-pericalcarine | 0.041915 | 0.802698 | 0.923102 |
| executive fuction | lh-postcentral | 0.062616 | 0.708803 | 0.869465 |
| executive fuction | lh-posteriorcingulate | 0.027781 | 0.8685 | 0.948999 |
| executive fuction | lh-precentral | 0.203748 | 0.219842 | 0.563903 |
| executive fuction | lh-precuneus | 0.272985 | 0.097268 | 0.493425 |
| executive fuction | lh-rostralanteriorcingulate | 0.029325 | 0.861261 | 0.948999 |
| executive fuction | lh-rostralmiddlefrontal | 0.254031 | 0.123802 | 0.509451 |
| executive fuction | lh-superiorfrontal | 0.354039 | 0.029205 | 0.451068 |
| executive fuction | lh-superiorparietal | -0.013559 | 0.935604 | 0.960665 |
| executive fuction | lh-superiortemporal | 0.189929 | 0.253402 | 0.569261 |
| executive fuction | lh-supramarginal | 0.281074 | 0.087371 | 0.480893 |
| executive fuction | lh-frontalpole | 0.264166 | 0.109013 | 0.506526 |
| executive fuction | lh-temporalpole | 0.023814 | 0.887147 | 0.95376 |
| executive fuction | lh-transversetemporal | 0.392294 | 0.01485 | 0.451068 |
| executive fuction | lh-insula | -0.10992 | 0.511211 | 0.768488 |
| executive fuction | rh-bankssts | 0.16958 | 0.308756 | 0.617813 |
| executive fuction | rh-caudalanteriorcingulate | 0.064161 | 0.701944 | 0.865668 |
| executive fuction | rh-caudalmiddlefrontal | 0.084469 | 0.614115 | 0.8212 |
| executive fuction | rh-cuneus | 0.103254 | 0.537306 | 0.779687 |
| executive fuction | rh-entorhinal | 0.245467 | 0.137425 | 0.509451 |
| executive fuction | rh-fusiform | -0.085581 | 0.609441 | 0.8212 |
| executive fuction | rh-inferiorparietal | 0.318011 | 0.05169 | 0.451068 |
| executive fuction | rh-inferiortemporal | 0.017052 | 0.919064 | 0.956492 |
| executive fuction | rh-isthmuscingulate | 0.260212 | 0.114614 | 0.509451 |
| executive fuction | rh-lateraloccipital | 0.279813 | 0.088861 | 0.480893 |
| executive fuction | rh-lateralorbitofrontal | 0.234562 | 0.156336 | 0.509451 |
| executive fuction | rh-lingual | -0.104595 | 0.532006 | 0.778982 |
| executive fuction | rh-medialorbitofrontal | 0.212153 | 0.200994 | 0.561545 |
| executive fuction | rh-middletemporal | 0.272715 | 0.097612 | 0.493425 |
| executive fuction | rh-parahippocampal | 0.048321 | 0.773278 | 0.91119 |
| executive fuction | rh-paracentral | 0.045201 | 0.787572 | 0.912552 |
| executive fuction | rh-parsopercularis | 0.330087 | 0.042976 | 0.451068 |
| executive fuction | rh-parsorbitalis | 0.420979 | 0.008489 | 0.451068 |
| executive fuction | rh-parstriangularis | 0.142255 | 0.39423 | 0.684864 |
| executive fuction | rh-pericalcarine | 0.086997 | 0.603511 | 0.8212 |
| executive fuction | rh-postcentral | 0.042447 | 0.800242 | 0.922585 |
| executive fuction | rh-posteriorcingulate | -0.014011 | 0.933461 | 0.960609 |
| executive fuction | rh-precentral | 0.210839 | 0.203863 | 0.561545 |
| executive fuction | rh-precuneus | 0.278445 | 0.090498 | 0.484062 |
| executive fuction | rh-rostralanteriorcingulate | -0.008792 | 0.958218 | 0.977013 |
| executive fuction | rh-rostralmiddlefrontal | 0.252389 | 0.126332 | 0.509451 |
| executive fuction | rh-superiorfrontal | 0.268481 | 0.103139 | 0.504721 |
| executive fuction | rh-superiorparietal | 0.037958 | 0.821005 | 0.937128 |
| executive fuction | rh-superiortemporal | 0.106766 | 0.523479 | 0.77198 |
| executive fuction | rh-supramarginal | 0.225365 | 0.173699 | 0.539875 |
| executive fuction | rh-frontalpole | 0.319522 | 0.050527 | 0.451068 |
| executive fuction | rh-temporalpole | -0.080046 | 0.632849 | 0.838935 |
| executive fuction | rh-transversetemporal | 0.386876 | 0.016419 | 0.451068 |
| executive fuction | rh-insula | -0.067969 | 0.685139 | 0.858757 |

**Table S20. Screening Results of Target Brain Regions for Cognitive Domains**

| **Cognitive domain**  **(Y)** | **Verified M-Y associated brain regions**  **(gray matter volume - cognitive domain)** | **Verified X-Y associated brain regions**  **(FDG metabolism - cognitive domain)** | **Target brain regions**  **(M) and description of the mediation model** | **Model priority** |
| --- | --- | --- | --- | --- |
| **Executive function** | Right amygdala (r = 0.47, FDR-P = 0.004), left amygdala (r = 0.44, FDR-P = 0.001), | Left inferior parietal lobule (r=0.35, FDR-p = 0.015), left transverse temporal cortex (r = 0.34, FDR-P = 0.017) | No naturally co-occurring brain regions. Target brain regions: FDG metabolic value of the left inferior parietal lobule + gray matter volume of the right amygdala | High (both X-Y and M-Y association strengths meet statistical requirements) |
| **Visuospatial ability** | Right inferior parietal lobule (r=0.54, FDR-P < 0.0001), left inferior parietal lobule (r=0.50, FDR-P = 0.0002) | Right posterior superior temporal gyrus (r = 0.443, FDR-P＜0.0001), left middle temporal cortex (r=0.439, FDR-P＜0.0001) | No naturally co-occurring brain regions. Target brain regions: FDG metabolic value of the right posterior superior temporal gyrus + left inferior parietal lobule | Highest (both X-Y and M-Y correlation coefficients >0.3, with high association strength) |
| **Language ability** | left lateral orbitofrontal cortex (r = 0.39, FDR-P = 0.007) , right caudal middle frontal (r = 0.37, FDR-P = 0.006), left superior temporal cortex (r = 0.36, FDR-P = 0.007) | left superior temporal cortex (r = 0.41, FDR-P = 0.003), left pars opercularis cortex (r = 0.40, FDR-P = 0.009) | Naturally co-occurring brain region: left superior temporal cortex (participates in both M-Y and X-Y associations, both FDR-P < 0.01) | Highest (presence of naturally co-occurring brain regions, strong model persuasiveness) |
| **Memory** | No significant M-Y associated brain regions (all P>0.05) | right posterior cingulate cortex（r = 0.319, FDR-P = 0.045）, left posterior cingulate cortex（r = 0.329, FDR-P = 0.042） | No valid target brain regions (no M-Y association, failing to meet the prerequisites of mediation effect) | model construction not recommended) |
| **Attention** | Among 33 candidate brain regions, right supramarginal gyrus (r = 0.38, FDR-P = 0.006) , precentral gyrus (r = 0.38, FDR-P = 0.006) | No significant X-Y associated brain regions (all P>0.05) | No valid target brain regions (no X-Y association, failing to meet the prerequisites of mediation effect) | Model construction not recommended |

Based on the above screening results, the pairwise correlations between the independent variable (brain region FDG metabolism), candidate mediating variable (brain region gray matter volume), and dependent variable (cognitive function score) were verified to ensure that all three had significant correlations (P<0.05). Brain regions with no clinical association were excluded, and the mediating variables and path relationships of the three models were finally determined.

**Table S21.** ApoE-Adjusted Mediation Analysis and Multiple Imputation Sensitivity Analysis for Visuospatial Ability

| **Cognitive function** | **Path relationship** | **Analysis** | **X→M path** | **M→Y path** | **Total effect** | **Direct effect** | **Mediation effect** | **Proportion of mediation** | **Model fit** |
| --- | --- | --- | --- | --- | --- | --- | --- | --- | --- |
| Visuospatial ability | Right posterior superior temporal gyrus FDG (X) → right inferior parietal lobule volume (M) → visuospatial score (Y) | Original complete-case analysis | β = 2527.05, P = 0.001 | β = 0.0001, P = 0.100 | β = 0.87, P< 0.001 | β = 0.73, P< 0.001 | Effect = 0.135, 95% CI [0.006, 0.328] | 15.59% | R² = 0.488, P < 0.001 |
|  |  | Multiple-imputation mediation analysis | β = 2131.73, 95% CI [863.89, 3399.577], P = 0.001 | β = 0.0002, 95% CI [−0.000020, 0.000325], P= 0.086 | β = 1.93, 95% CI [0.771, 3.09], P = 0.001 | β = 1.61, 95% CI [0.35, 2.86], P= 0.012 | Effect = 0.321618, 95% CI [−0.079, 0.72], P= 0.115 | 16.67%* | — |

β, standardized regression coefficient; CI, 95% confidence interval. * p < 0.05, ** p < 0.01, *** p < 0.001. The proportion of mediation was calculated as the ratio of indirect effect to total effect.

**Table S22.** ApoE-Adjusted Mediation Analysis and Multiple Imputation Sensitivity Analysis for Executive function

| **Cognitive function** | **Path relationship** | **Analysis** | **X→M path** | **M→Y path** | **Total effect** | **Direct effect** | **Mediation effect** | **Proportion of mediation** | **Model fit** |
| --- | --- | --- | --- | --- | --- | --- | --- | --- | --- |
| Executive function | lIPL FDG (X) → rAmygdala GMV (M) → executive function (Y) | Original complete-case mediation model | β = 0.143, 95% CI [−0.159, 0.438], P = 0.356 | β = 0.420, 95% CI [0.210, 0.665], P < 0.001 | β = 0.406 95% CI [0.146, 0.646], P= 0.002 | β = 0.346, 95% CI [0.078, 0.587], P= 0.008 | Effect = 0.060, 95% CI [−0.070, 0.213], P= 0.403 | 14.75%* | R² = 0.365, P＜0.001 |
|  |  | Multiple-imputation mediation analysis | β = 0.0003, 95% CI [0.00007, 0.0006], P= 0.014 | β = 0.911, 95% CI [0.077, 1.746],P = 0.033 | β = 0.002618, 95% CI [0.001573, 0.003663], *P* < 0.001 | β = 0.002329, 95% CI [0.001275, 0.003382], *P* < 0.001 | Effect = 0.000289, 95% CI [−0.000060, 0.000638], *P* = 0.106 | 11.04%* | — |

**Table S23.** ApoE-Adjusted Mediation Analysis and Multiple Imputation Sensitivity Analysis for Language ability

| **Cognitive function** | **Path relationship** | **Analysis** | **X→M path** | **M→Y path** | **Total effect** | **Direct effect** | **Mediation effect** | **Proportion of mediation** | **Model fit** |
| --- | --- | --- | --- | --- | --- | --- | --- | --- | --- |
| Languageability | Left superior temporal gyrus FDG (X) → left superior temporal gyrus volume (M) | Original complete-case mediation model | β = 0.553，95% CI [1547.251, 4422.608], P < 0.001 | β = 0.196，95% CI [−0.00001, 0.0005], P= 0.215 | β = 0.430，95% CI [0.665, 3.540], P = 0.002 | β = 0.322，95% CI [−0.599, 3.252], P = 0.074 | β = 0.108, 95% CI [−0.289, 2.006],P = 0.302 | 25.2% | R² = 0.288, P＜0.001 |
|  |  | Multiple-imputation mediation analysis | β = 0.492，95% CI [0.316, 0.668], P< 0.001, | β = 0.251，95% CI [0.004, 0.497], P= 0.046 | β = 0.240，95% CI [0.046, 0.435], P= 0.015 | β = 0.117, 95% CI [−0.120, 0.354], P= 0.332 | β = 0.123，95% CI [−0.013, 0.259], P = 0.076 | 51.2%* | — |

β, standardized regression coefficient; CI, 95% confidence interval. * p < 0.05, ** p < 0.01, *** p < 0.001. The proportion of mediation was calculated as the ratio of indirect effect to total effect.

**Table S24.** Sex-related differences in regional Aβ deposition in the non-AD group

| **Group** | **Brain region** | **Male(n)** | **Female(n)** | **Male adjusted mean** | **Female adjusted mean** | **Female minus Male** | **P value** | **R squared** | **FDR -P** |
| --- | --- | --- | --- | --- | --- | --- | --- | --- | --- |
| Non-AD | lh-cuneus | 19 | 23 | 0.980 | 0.895 | -0.085 | 0.000 | 0.434 | 0.018* |
| Non-AD | lh-pericalcarine | 19 | 23 | 1.091 | 0.978 | -0.113 | 0.000 | 0.380 | 0.018* |
| Non-AD | Left-Putamen | 19 | 23 | 1.156 | 1.045 | -0.110 | 0.001 | 0.293 | 0.024* |
| Non-AD | rh-isthmuscingulate | 19 | 23 | 1.008 | 0.923 | -0.085 | 0.001 | 0.394 | 0.024* |
| Non-AD | rh-precuneus | 19 | 23 | 1.014 | 0.919 | -0.095 | 0.001 | 0.339 | 0.024* |
| Non-AD | lh-precuneus | 19 | 23 | 0.992 | 0.892 | -0.100 | 0.002 | 0.345 | 0.027* |
| Non-AD | rh-posteriorcingulate | 19 | 23 | 1.019 | 0.920 | -0.100 | 0.002 | 0.297 | 0.029* |
| Non-AD | lh-posteriorcingulate | 19 | 23 | 0.991 | 0.893 | -0.098 | 0.004 | 0.374 | 0.049* |

*FDR-P≤0.05, statistically significant difference.
